# Semi-Quantitatively Designing Two-Photon High-Performance Fluorescent Probes for Glutathione S-Transferases

**DOI:** 10.34133/2020/7043124

**Published:** 2020-03-18

**Authors:** Xue-Xiang Zhang, Huan Qi, Mei-Heng Lu, Song-Qiu Yang, Peng Li, Hai-long Piao, Ke-Li Han

**Affiliations:** ^1^State Key Laboratory of Molecular Reaction Dynamics, Dalian Institute of Chemical Physics, Chinese Academy of Sciences, Dalian 116023, China; ^2^CAS Key Laboratory of Separation Science for Analytical Chemistry, Dalian Institute of Chemical Physics, Chinese Academy of Sciences, Dalian 116023, China; ^3^Institute of Molecular Sciences and Engineering, Shandong University, Qingdao 266237, China

## Abstract

Glutathione S-transferases (GSTs), detoxification enzymes that catalyze the addition of glutathione (GSH) to diverse electrophilic molecules, are often overexpressed in various tumor cells. While fluorescent probes for GSTs have often adopted the 2,4-dinitrobenzenesulfonyl (DNs) group as the receptor unit, they usually suffer from considerable background reaction noise with GSH due to excessive electron deficiency. However, weakening this reactivity is generally accompanied by loss of sensitivity for GSTs, and therefore, finely turning down the reactivity while maintaining certain sensitivity is critical for developing a practical probe. Here, we report a rational semiquantitative strategy for designing such a practical two-photon probe by introducing a parameter adopted from the conceptual density functional theory (CDFT), the local electrophilicity *ω*_*k*_, to characterize this reactivity. As expected, kinetic studies established *ω*_*k*_ as efficient to predict the reactivity with GSH, and probe NI3 showing the best performance was successfully applied to detecting GST activities in live cells and tissue sections with high sensitivity and signal-to-noise ratio. Photoinduced electron transfer of naphthalimide-based probes, captured by femtosecond transient absorption for the first time and unraveled by theoretical calculations, also contributes to the negligible background noise.

## 1. Introduction

Glutathione S-transferases (GSTs, EC 2.5.1.18), mainly known as phase II detoxifying enzymes [1], are a family of dimeric enzymes that catalyze the nucleophilic attack of the sulfhydryl of glutathione (GSH) on an electrophilic center of diverse substrates of endogenous or exogenous origin [2]. The expression level of GSTs plays a crucial role in determining the susceptibility to cancer chemotherapy [3]. Among varieties of GST isoenzymes, alpha (GSTA), mu (GSTM), and pi (GSTP) are frequently found overexpressed in various tumor cell lines, particularly in anticancer drug-resistant ones [4–8]. Hence, sensitively and specifically monitoring GST activities in biological systems without background noise, namely, false-positive error usually introduced by GSH, is urgently needed.

Recently, small-molecule fluorescent probes have been rapidly emerging as a powerful tool for enzyme detection in biological samples by virtue of their fast analysis, higher sensitivity, minimal perturbation to living systems, and real-time detection capabilities [9–13]. Indeed, several such probes have been developed for sensitive detection of GST activities with representatives being DNAT-Me [14], DNs-CV [15], and 3,4-DNADCF [16]. However, these probes exhibit either high nonenzymatic background noise or narrow isoenzyme selectivity. Specifically, while the 2,4-dinitrobenzenesulfonyl (DNs) group has often been employed as a receptor unit for GST probes [15, 17], those probes for thiols such as GSH and cysteine mostly just adopt the same group [18–20], demonstrating the nonnegligible background noise due to the nonenzymatic reaction between GSH and this very group. Given the considerable concentration of GSH (*ca.* 1–10 mM) in mammalian cells, interferences from this GSH noise with GST detection should not be ignored. However, a probe with higher sensitivity for GSTs is usually accompanied by a higher nonenzymatic background noise due to its chemical reactivity with GSH, which implies that alleviating this noise is also at the expense of sensitivity. Therefore, finely tuning the reactivity with GSH is critical for designing a practical probe for GSTs with both specificity and sensitivity.

It is conceivable that an effective parameter characterizing the reactivity of one GST probe with GSH should be conducive to molecular design for the sake of subtle tuning. It is well documented that GST catalyzes the nucleophilic attack of GSH on the electrophilic center of its substrate via nucleophilic aromatic substitution (S_N_Ar) reaction mechanism [1, 21], so what is desired should be a parameter reflecting the effective electrophilicity of a probe. Therefore, we turned to the local electrophilicity *ω*_*k*_ [22], a concept quoted from conceptual density functional theory (CDFT), which has been extensively employed to investigate Diels-Alder reactions [23–26]. As shown in Equation (1), *ω*_*k*_ is equal to the arithmetic product of the global electrophilicity *ω* [27] and the electrophilic Parr function *P*_*k*_^+^ [22], the latter one being an approximation of the condensed Fukui function *f*_*k*_^+^, which characterizes the regioselectivity. With GSH generally accepted as a so-called soft nucleophile and nitrobenzene derivative a soft electrophile [28], it is reasonable to use this parameter to describe the reactivity [29, 30].

(1) $$\begin{array}{c} \omega_{k}=\omega \cdot P_{k}^{+}. \end{array}$$

Here, we present the first rational semiquantitative strategy for designing practical two-photon fluorescent probes for GSTs with both high sensitivity and negligible background noise. First, based on the S_N_Ar reaction mechanism, we established that the electrophilic Parr function *P*_*k*_^+^ could characterize the regioselectivity of a probe, and therefore, the local electrophilicity *ω*_*k*_ can be used to represent and predict relative chemical reactivity of different probes. Hence, a series of probe candidates were designed and screened out according to their *ω*_*k*_ values, which were available by quantum chemical calculations. These probes were synthesized and evaluated in terms of sensitivity and signal-to-noise (S/N) ratio, after which **NI3** was selected and successfully applied to the imaging of GST activities in live cells and tissue sections with high sensitivity and S/N ratio. Furthermore, femtosecond transient absorption spectra and time-dependent density functional theory (TD-DFT) calculations revealed the photoinduced electron transfer (PET) mechanism of fluorescence quenching, which also contributed to the considerably low background noise.

## 2. Results

### 2.1. Designing and Screening of the Probe Candidates

**Figure 1:**
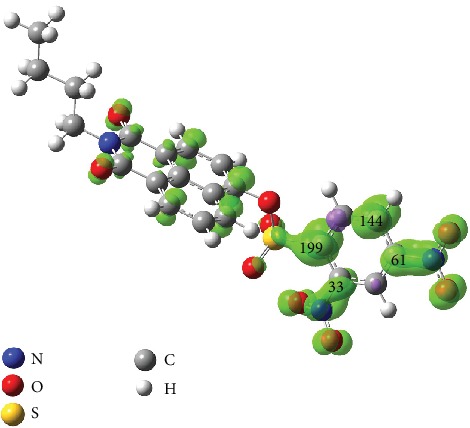
Spin density distribution of the anion radical of **NI1**. Respective positive *P*_*k*_^+^ value (amplified by a factor of 1,000) of the carbon atoms in the nitrobenzene ring is marked, with positive and negative spin density colored by green and purple, respectively. Negative spin density or *P*_*k*_^+^ values are herein regarded as meaningless [35, 36]. Isodensity value = 0.002.

As stated earlier, the DNs group has often been employed as a receptor unit for GST detection probes; we thus started to design our first two-photon fluorescent probe candidate **NI1** by introducing the DNs group to the ring of 4-hydroxyl-*N*-butyl-1,8-naphthalimide (**NI**), a well-known fluorophore with two-photon absorptivity [31, 32]. Initially, to examine whether the calculation method introduced here is rational, the electronic spin density distribution and the atomic spin population (namely, the *P*_*k*_^+^ values) of the anion radical of **NI1** were calculated. The *α*-carbon of the aryl-sulfonyl group showed the maximum spin density and *P*_*k*_^+^ (Figure 1), indicating the most electrophilic center lies on this very carbon, which is to be attacked by GSH, in accord with the regioselectivity revealed by previous experiments [15, 17]. This result indicates that it is the electronic aspects related to electrophilicity rather than other factors such as the leaving ability of the nucleofuge related to nuclear displacement [33, 34] that dominate the regioselectivity herein. Confirming *P*_*k*_^+^'s ability to reproduce the real regioselectivity led to the conclusion that the effective reactivity of a probe with GSH could be characterized by the local electrophilicity *ω*_*k*_ of the *α*-carbon (refer to Equation (1)). It can be envisaged that by altering substitution situations on the nitrobenzene ring and comparing *ω*_*k*_ of resultant probe candidates, some superior probes will be preliminarily screened out with the criterion: the *ω*_*k*_ of a practical probe should be modestly lower than that of **NI1**.

**Figure 2:**
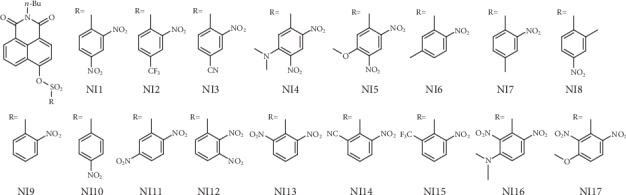
Chemical structures of the probe candidates.

Considering the DNs group is a much too sensitive receptor unit, five approaches were put forward (Figure 2): (1) replace the second nitro group, the one *para* to the *α*-carbon, with less electron-withdrawing groups to give **NI2** and **NI3**; (2) add an electron-donating group to the position *meta* to the *α*-carbon to give **NI4** and **NI5**; (3) simply shift the second nitro group to other positions to give **NI11**, **NI12**, and **NI13**; (4) first, shift the second nitro group to the other position *ortho* to the *α*-carbon, and then, replace it with less electron-withdrawing groups to give **NI14** and **NI15**; and (5) first, shift the second nitro group to the other position *ortho* to the *α*-carbon, and then, add an electron-donating group to the position *meta* to the *α*-carbon to give **NI16** and **NI17**. Subsequently, these compounds were evaluated in terms of spin density distribution, *P*_*k*_^+^ and *ω*_*k*_ values, and the results showed that all probe candidates except those in the third approach displayed a valid regioselectivity and reasonably lower *ω*_*k*_ values with **NI5**, **NI3**, and **NI14** among the most moderate ones, suggestive of their potential as practical probes (Figure S1 and Table S1). It should be noted that similar *ω*_*k*_ values appear in **NI1** and **NI13**, **NI3** and **NI14**, **NI2** and **NI15**, or **NI4** and **NI16**, respectively, indicating that there is no significant difference in the electron-withdrawing group's locating *para* or *ortho* to the *α*-carbon, which is in line with the chemical intuition. As for **NI11** and **NI12**, the most electrophilic center lies on the alternative *β*-carbon rather than on the *α*-carbon (Figure S1), indicative of their inappropriateness as GST detection probes. Actually, one tries synthesizing **NI11** but only to find that apart from the fluorine atom, one nitro group was substituted competitively and comparably by the sulfonic group just via the S_N_Ar reaction mechanism (Figure S2; refer to the synthesis section below and in Supplementary Materials; for more evidence for the applications of *P*_*k*_^+^ and *ω*_*k*_, refer to Figures S3–4 and Tables S2–3).

Taking both the above results and the practical complexity of chemosynthesis into account, we determined to synthesize **NI5**, **NI3**, **NI4**, and **NI2**, with **NI1** also prepared as a representative for oversensitive probes. In addition, since this is the first time *ω*_*k*_ has been introduced for designing GST detection probes, less sensitive receptor units which emerged in the previous literature [3, 15] were also adopted to give **NI6**, **NI7**, **NI8**, **NI9**, and **NI10** to reveal the relationship between chemical structures and sensitivities in more detail. Notably, although they share the same regioselectivity as that of **NI1** (Figure S1), their *ω*_*k*_ values are somewhat too minor relative to the latter's (Table S1).

### 2.2. Synthesis and *In Vitro* Evaluation of the Probe Candidates

To synthesize these probe candidates, a facile three-step procedure was adopted from starting materials either commercially available or conveniently synthesized (refer to Supplementary Materials). In essence, a sulfonic group was introduced by treating the appropriate nitro-fluorobenzene derivative with sodium sulfite in a solvent mixture of water and ethanol, the reaction mechanism of which happens to be S_N_Ar as well, followed by chlorination with thionyl chloride or phosphoryl chloride to give the receptor unit, which was then readily attached to the hydroxyl of **NI** to prepare the final compound. It is worth mentioning that this synthetic route circumvents the conventional method calling for poisonous gas SO_2_. Compounds **NI1**–**NI10** and relevant intermediate products were fully characterized using NMR spectroscopy (Figures S20–S57) and mass spectrometry.

**Figure 3:**
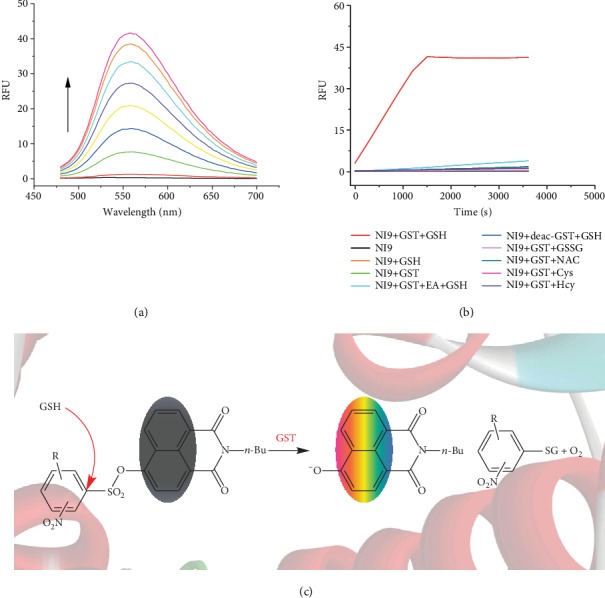
*In vitro* GST detection with NI9 and the detection mechanism for **NI**-series probes. (a) Time-dependent fluorescence spectra of **NI9** (20 *μ*M) in HEPES buffer (20 mM, 0.5% DMSO, pH 7.4) upon addition of GSTs (12.5 *μ*g/mL) over the course of 25 min at 37°C in the presence of GSH (2 mM). *λ*_ex_ = 445 nm. (b) A full set of control experiments on examining the cause of fluorescence enhancement. EA = ethacrynic acid (GSTs were preincubated with 200 *μ*M EA for 30 min before addition of GSH and **NI9** sequentially); deac-GST = deactivated GSTs (12.5 *μ*g/mL) by pretreatment at 100°C for 10 min; GSSG = oxidized glutathione (2 mM); NAC = *N*-acetylcysteine (2 mM); Cys = *L*-cysteine (2 mM); Hcy = *L*-homocysteine (2 mM). *λ*_ex/em_ = 445/560 nm. (c) Schematic for the proposed GST activity detection mechanism of **NI**-series probes.

With these probe candidates in hand, we investigated whether they were amenable to GST detection *in vitro*. On the whole, upon encounter with GSTs from the equine liver, all these probe candidates gained a drastic enhancement in fluorescence intensity from a virtually nonluminescence state, despite their different S/N ratios (Figure S5). For instance, **NI9** showed beyond 35-fold fluorescence increase at 560 nm in less than 30 minutes (Figure 3(a)). To confirm the probe was lightened by no other than GST activities, a full set of control experiments were conducted. As shown in Figure 3(b), hardly any fluorescence was triggered without either of GST and GSH or both, using deactivated GSTs or replacing the GSH with other sulphur-bearing analogues, namely, oxidized glutathione, *N*-acetylcysteine, *L*-cysteine, and *L*-homocysteine. In addition, if ethacrynic acid (EA), a well-known inhibitor for various GSTs [37], was added 30 min prior to GSH and **NI9**, the rising of fluorescence was substantially suppressed (Figure 3(b)). These results demonstrate that GST and GSH are both indispensable to the fluorescence enhancement. Further, with both of the resultant organic products being captured and tracked, the UPLC-MS analysis (Figure S6) as well as the spectra comparison (Figure S7) provided a more explicit and solid evidence that the detection mechanism is exactly the one illustrated in Figure 3(c). As designed, under the catalysis of GST, the attack of GSH on the *α*-carbon releases glutathione conjugate, SO_2_, and **NI**, enabling the detection of GST activities.

**Figure 4:**
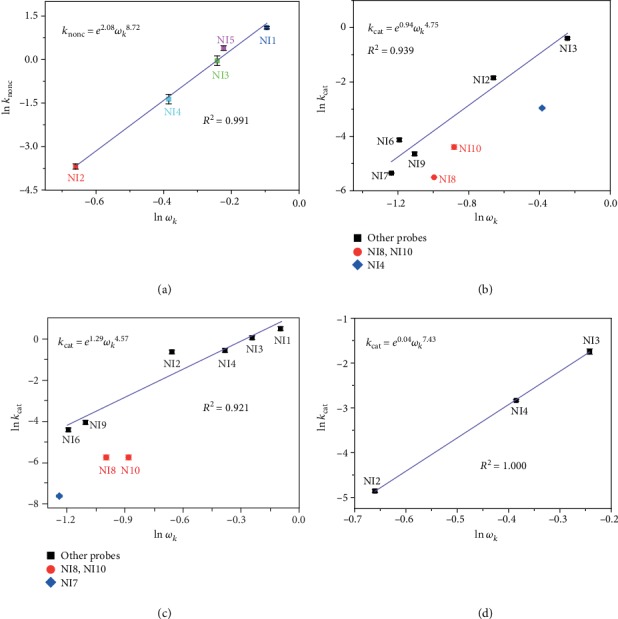
Structure-activity relationships between nonenzymatic or enzymatic kinetic parameters and the local electrophilicity *ω_k_* of the *α*-carbon. (a) Regarding the second-order rate constant *k*_nonc_ of the nonenzymatic reaction between GSH and probes. Data for other probes are unavailable due to extremely low reactivity. (b–d) Regarding the catalytic constant *k*_cat_ of (b) GSTA1-1, (c) GSTM1-1, and (d) GSTP1-1, respectively, towards different probes. Linear fittings were based on mean values. Error bars represent SD.

Although every single probe did display measurable response towards GSTs, they differed greatly from one another in terms of sensitivity and nonenzymatic noise. For example, with rapid response to either GSTs or GSH, **NI1** is regarded as an oversensitive probe, whereas **NI6** belongs to undersensitive probes due to slow response towards GSTs, along with no background noise at all (Figures S5a,f and S8). In order to inspect the relationship between chemical structures of the probes and their performances quantitatively, an elaborate kinetic study on both nonenzymatic and enzymatic reactions was implemented, and the kinetic parameters were plotted as the function of *ω*_*k*_ values of the *α*-carbon (Figure 4 and Tables S4–S6). With all the probes whose nonenzymatic reactions with GSH are detectable falling into the quasilinear plot of ln *k*_nonc_ and ln *ω*_*k*_ (the goodness of fit *R*^2^ = 0.991), the local electrophilicity *ω*_*k*_ proved itself an excellent parameter to describe the S_N_Ar reactivity with GSH, namely, the nonenzymatic noise for GST detection. Remarkably, the order of *k*_nonc_ for different probes (**NI2**<**NI4**<**NI3**<**NI5**<**NI1**) agrees quite well with the order of fluorescence rising rate in preceding nonenzymatic tests (Figure S8b), thus corroborating the conclusions drawn here. To our knowledge, this is the first time the nonenzymatic noises of probes for GST detection have been depicted and predicted by a single parameter (see Figure S9 for more evidence). Furthermore, to some extent, this parameter can also reflect the probe's sensitivity to GSTs (Figures 4(b)–4(d)). Remarkably, the data of **NI8** and **NI10** deviate from the description by *ω*_*k*_, demonstrating the *o*-NO_2_ is indispensable to GST catalysis, in agreement with the previous literature [1, 15]. In general, a smaller *ω*_*k*_ means a lower background noise and yet probably a lower sensitivity meanwhile. Then, to what extent do these two aspects depend on the chemical structure, namely, *ω*_*k*_?

Notably, the preexponential factor (2.08) and exponential term (8.72) of the fitting formula for the nonenzymatic reaction are both larger than the ones for enzymatic reactions (0.94 and 4.75 for GSTA1-1; 1.29 and 4.57 for GSTM1-1; and 0.04 and 7.43 for GSTP1-1), respectively (Figure 4), implying that improving the sensitivity (here depicted by *k*_cat_) by enlarging the *ω*_*k*_ would be monotonically accompanied by the loss of S/N ratio (here depicted by *k*_cat_/*k*_nonc_), in good agreement with preceding results (Figures S5 and S8). Thus, there is a trade-off between sensitivity and S/N ratio, and reaching a balance point between them was critical for a superb probe. Fortunately, the relationships above can also be reviewed from the other side: starting from an oversensitive probe such as **NI1**, a tiny decrease of *ω*_*k*_ would probably afford a drastic reduction in nonenzymatic noise while leaving the sensitivity almost unchanged (*cf. ***NI3** and **NI1** in Figures 4(a) and 4(c); see also Figure S8b), thus obtaining a probe with both high sensitivity and high S/N ratio, self-supporting the design strategy here quantitatively. Therefore, it is unsurprising that after an overall consideration of sensitivity, S/N ratio, and broad isoenzyme selectivity (Table S7), **NI3**, one of the two probes whose *ω*_*k*_ are modestly lower than that of **NI1** (Table S1), was found to be the best one. It was thus used for more practical and rigorous applications such as bioimaging in the next section.

### 2.3. Fluorescence Imaging of Live Cells and Tissue Sections

**Figure 5:**
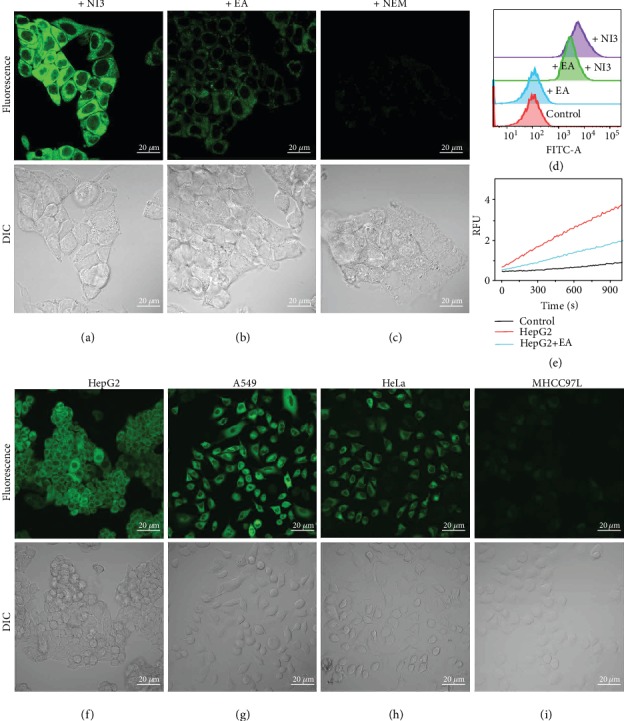
(a–c) Single-photon confocal fluorescence imaging of HepG2 cells (a) incubated with 20 *μ*M **NI3**, (b) pretreated with 100 *μ*M EA and then incubated with 20 *μ*M **NI3**, and (c) pretreated with 50 *μ*M NEM and then incubated with 20 *μ*M **NI3** with a 100x objective. *λ*_ex_ = 458 nm. *λ*_em_ = 500–600 nm. Scale bar = 20 *μ*m. (d) Flow cytometric analysis of the HepG2 cells. *λ*_ex_ = 488 nm. *λ*_em_ = 500–600 nm. (e) Assay of the HepG2 cell lysate using 20 *μ*M **NI3** with 1 mM GSH added in. “Control” means assay without cell lysate. *λ*_ex_ = 445 nm. *λ*_em_ = 560 nm. (f–i) Fluorescence images of various cell lines incubated with 20 *μ*M **NI3** for 30 min with a 40x objective. *λ*_ex_ = 458 nm. *λ*_em_ = 500–600 nm. Scale bar = 20 *μ*m. Representative images from repeated experiments are shown.

It is often desirable to be able to detect very low levels of enzymatic activities in live cells, cell extracts, or tissues. Hence, **NI3** was examined whether it was capable of reporting GST activities by cellular fluorescence imaging. First, HepG2 was selected as an appropriate cell line for GST monitoring according to the Western blotting results (Figure S10). Incubation with 20 *μ*M **NI3** in HEPES buffer (pH 7.4) for just one minute could afford a discernible fluorescence image derived from a completely dark one when no probe was added, and then, the cells gradually became brighter in the following *ca.* 30 min (Figure S11 and Video 1), demonstrating the probe was ignited by the intracellular substances. To ascertain the fluorescence arose from GST activities, cells were pretreated with the GST inhibitor EA and the GSH-depleting agent *N*-ethylmaleimide (NEM), respectively, before the addition of **NI3**. The results showed a substantial loss of fluorescence for both cases (Figures 5(a)–5(c)), proving that it was the attack of GSH on the probe by virtue of GST catalysis that lighted the cells up. A similar consequence was obtained from the flow cytometric analysis (Figure 5(d) and Table S8). Furthermore, an analogous assay in cell lysate samples revealed that the fluorescence intensity of cell lysate pretreated with EA was still obviously higher than that of the control group which represented the nonenzymatic reaction of **NI3** with GSH (Figure 5(e)). This finding implies the remaining fluorescence that appeared in cellular imaging or flow cytometry came from the residual GST activities other than the GSH itself (Figures 5(b) and 5(d)) and thus highlights the remarkably high sensitivity and S/N ratio of **NI3**. Actually, the limit of detection (3*σ*) was calculated to be 3.7 nM. Moreover, to further verify that the nonenzymatic reaction produces little noise, we selected MHCC97L cells as a negative control and HepG2 together with A549 and HeLa cells as positive controls based on the Western blotting results (Figures S10). After being subjected to **NI3** incubation and fluorescence imaging, MHCC97L cells displayed little fluorescence, whereas other cells exhibited strong emission under the same conditions (Figures 5(f)–5(i)). With almost no GST in the MHCC97L cell line and varied GST isoenzymes in each individual positive cell line, this result manifested the probe's specificity for GSTs with negligible nonenzymatic noise again and broad isoenzyme selectivity as well. Incidentally, when incubated with **NI3**, all cells kept a normal and fine morphology during the whole imaging course, indicating its low cytotoxicity and favorable biocompatibility. In addition, to investigate the source of the GST activities, the colocalization experiment was performed and the results showed the fluorescence spread all over the cytoplasm homogeneously rather than focusing on any organelles (Figures S12–S14), consistent with the GSTs' coming from the whole cytosol. Notably, when HepG2 cells were incubated with 20 *μ*M **NI3** or **NI2** for 30 min, respectively, both cellular imaging and flow cytometry tests exhibited a weaker fluorescence for the latter (Figure S15). Additionally, when pretreated with EA and then incubated with **NI1**, HepG2 cells showed almost the same fluorescence intensity as incubated with only **NI1** (Figure S16), highlighting **NI1**'s nonnegligible background noise. All these consequences were in agreement with previous *in vitro* results, demonstrating the local electrophilicity *ω*_*k*_ does affect a probe's effective performances.

**Figure 6:**
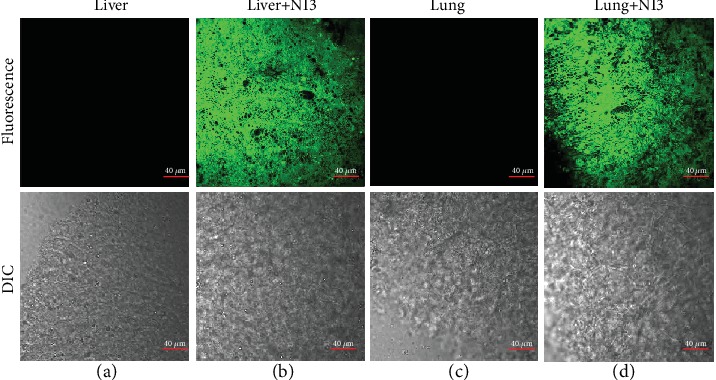
Two-photon confocal fluorescence imaging of mouse (a, b) liver and (c, d) lung tissue sections incubated with or without 40 *μ*M **NI3** at a depth of 60 *μ*m with a 60x objective. Scale bar = 40 *μ*m. *λ*_ex_ = 810 nm. *λ*_em_ = 520–560 nm.

With its ability to detect GST activities certified by single-photon confocal fluorescence imaging, we next tested if **NI3** was amenable to two-photon imaging of more complicated biological samples. Initially, identical consequences were obtained by two-photon cell imaging upon irradiation at 810 nm with a femtosecond pulse laser (Figure S17), establishing the probe's capacity for applications based on two-photon microscopy. Subsequently, **NI3** was interrogated as to whether it could afford clear images of tissue sections containing plenty of GSTs. Hence, the tissues of the liver and lung from female BALB/c mice were cut to 100 *μ*m slices with a frozen slicer and then soaked into the HEPES buffer containing 40 *μ*M **NI3** for 1 h before two-photon microimaging. As shown in Figures 6(b) and 6(d), both liver and lung tissues displayed bright fluorescence, albeit with different GST isoenzymes in them [38, 39], whereas completely dark images were acquired in the absence of **NI3** (Figures 6(a) and 6(c)), with the negligible background autofluorescence benefiting from the near-infrared excitation wavelength implied by the two-photon absorptivity of the probe. Furthermore, fluorescence images of the liver tissue sections at different depths were collected in the Z-scan mode (Figure S18 and Video 2), and the results indicate **NI3** is able to realize tissue imaging as deep as *ca.* 100 *μ*m, which enables the 3D reconstruction of the tissue images (Figure S19). To sum up, these results exhibited the excellent two-photon staining and tissue-penetrating capabilities of **NI3**.

### 2.4. Fluorescence Quenching Mechanism of Intact NI-Series Probes

**Figure 7:**
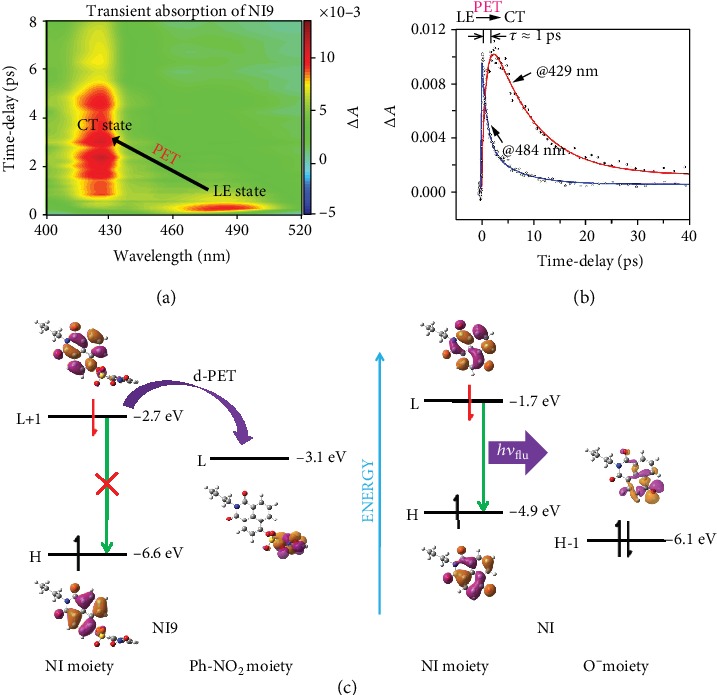
Experimental and theoretical studies on the fluorescence quenching mechanism. (a) Pseudocolor femtosecond transient absorption (TA) spectral plot of **NI9** in DMSO. (b) Kinetic traces at different wavelengths following the 370 nm laser pulse excitation and the respective fit with three exponential functions. (c) TD-DFT calculations on the electronic transitions of **NI9** and **NI** in DMSO at the B3LYP/aug-cc-pVDZ level.

The establishment of **NI3**'s outstanding performances is unavailable without the low background noise, which is intimately related not only to the appropriate nonenzymatic reactivity but also to the remarkable “off” state of the intact probe. Thus, it is high time to review the fluorescence quenching mechanism. As not merely **NI3** but all the other intact **NI**-series probes showed well-quenched fluorescence (Figure S5), the nitro group was considered to bring about this property, and thus, **NI9** was selected as the representative subjected to the subsequent exploration. We used femtosecond transient absorption (TA) spectroscopy to monitor spectral changes induced by excitation at 370 nm. Almost the moment the pump light was administrated (<120 fs), a photoinduced absorption band centered at around 484 nm was observed (Figures 7(a) and 7(b)), which is attributed to the formation of the locally excited (LE) state. Subsequently, the absorption of the LE state decreased gradually, accompanied by the emergence of a new absorption band centered at around 429 nm, indicative of the formation of a new state. It is remarkable that the timescales of the decay of the LE state and the formation of the new state are both 1 ps according to their respective fits, suggesting the new state was derived from the LE state, which caused the fluorescence quenching.

To elucidate this phenomenon and gain more insight, calculations on the electronic transitions of **NI9** and **NI** were implemented based on the TD-DFT method. As shown in Table S9, for **NI9**, a small oscillator strength of S_0_⟶S_1_ transition (*f* = 0.009) suggests a forbidden transition, demonstrating the S_1_ state of **NI9** is not accessible directly from the S_0_ state. However, it might be populated via internal conversion from the S_2_ state, which arises from a considerable oscillator strength of S_0_⟶S_2_ transition (*f* = 0.382). This result is corroborated by the agreement between experimental (360 nm) and calculated (356 nm) maximum absorption wavelengths. The S_0_⟶S_2_ transition is dominated by the transition of HOMO to LUMO+1, both of which are located on the **NI** moiety, exhibiting a LE characteristic; the S_0_⟶S_1_ transition is dominated by the transition of HOMO to LUMO, the latter being located on the nitrobenzene moiety, exhibiting a charge-transfer (CT) characteristic (Figure 7(c), left). Hence, a donor-excited photoinduced electron transfer (d-PET) process occurred from the **NI** moiety to the nitrobenzene moiety upon excitation owing to the driven force derived from the energy (-2.7 and -3.1 eV for LUMO+1 and LUMO, respectively) gap, quenching the fluorescence, and the new state formed in Figures 7(a) and 7(b) corresponds to **NI9**'s S_1_ state, a dark state, also known as the CT state. To our knowledge, this is the first time the actual PET process of **NI**-based probes has been observed experimentally. As a comparison, for **NI**, the maximum oscillator strength appears in S_0_⟶S_1_ transition (*f* = 0.200), which is mainly contributed by the transition of HOMO to LUMO (Table S9). With both molecular orbitals settled on the **NI** moiety and no other state between S_0_ and S_1_ (LE state), its fluorescence shines out unrestrictedly (Figure 7(c), right), ensuring the excellent applications in bioimaging with **NI**-based probes. Taken together, the existence of a nitro group in the intact probe induces a d-PET process caging the fluorescence, avoiding a background noise arising from the probe itself.

## 3. Discussion

Finely turning down the nonenzymatic reactivity with GSH plays a pivotal role in reducing the background noise of a GST detection probe while maintaining a considerable sensitivity. We have adopted and established the local electrophilicity index *ω*_*k*_ as efficient to characterize this reactivity and thus developed a rational semiquantitative strategy to design a two-photon fluorescent probe for GSTs with high sensitivity and S/N ratio by evaluating its *ω*_*k*_. In this way, **NI3** has been selected, and both examinations *in vitro* and bioimaging in live cells or tissue sections verify its outstanding performances, demonstrating the feasibility of this strategy. Moreover, besides the modestly lower *ω*_*k*_, the success of achieving a low background noise depends on the caged fluorescence of intact probes by PET mechanism as well, which is observed for the first time by femtosecond TA spectra for **NI**-based probes, and a theoretical study based on TD-DFT calculations has explained how PET, and thus, fluorescence quenching happens.

In summary, this work highlights the start of introducing a parameter from CDFT to GST probe design. Theoretically speaking, the *ω*_*k*_ adopted here is not only limited to GST probe design but can be widely used for any probes based on S_N_Ar reactions between soft nucleophile and soft electrophile. Overall, we anticipate our strategy will inspire more high-performance probes like **NI3** to be applied to biomedical research in the future.

## Data Availability

All data needed to evaluate the conclusions in the paper are present in the paper and/or the Supplementary Materials. Any additional datasets, analysis details, and material recipes are available upon request.

## Supplementary Materials

Supplementary Materials Detailed experimental and computational methods, material preparations, characterizations, fluorescence imaging videos, and supplementary figures and tables are available in Supplementary Materials.
