# Supplementary material for: Semi-Quantitatively Designing Two-Photon High-Performance Fluorescent Probes for Glutathione S-Transferases
Source: Research (Wash D C). 2020 Mar 18;2020:7043124. doi: 10.34133/2020/7043124 (PMC7114728; doi:10.34133/2020/7043124)
Supplement: Supplementary Materials — Detailed experimental and computational methods, material preparations, characterizations, fluorescence imaging videos, and supplementary figures and tables are available in Supplementary Materials. [file 7043124.f1.zip › Supplementary Materials.pdf]

Supplementary Materials

Semi-Quantitatively Designing Two-Photon High-Performance Fluorescent Probes for Glutathione S-Transferases

Xue-Xiang Zhang[†,⊥,§], Huan Qi[‡,§], Mei-Heng Lu[†,⊥], Song-Qiu Yang[†], Peng Li[†,∥], Hai-long Piao[*,‡] and Ke-Li Han[*,†,∥]

[†] State Key Laboratory of Molecular Reaction Dynamics, Dalian Institute of Chemical Physics, Chinese Academy of Sciences, Dalian 116023, P. R. China.

[‡] CAS Key Laboratory of Separation Science for Analytical Chemistry, Dalian Institute of Chemical Physics, Chinese Academy of Sciences, Dalian 116023, P. R. China.

[⊥] University of the Chinese Academy of Sciences, Beijing 100049, P. R. China.

[∥] Institute of Molecular Sciences and Engineering, Shandong University, Qingdao 266237, P. R. China

*Corresponding authors. Emails: klhan@dicp.ac.cn; Hpiao@dicp.ac.cn

Table of Contents

Materials and Instruments.......... 3

Calculations of spin density distribution, P_{+k}, ω and ω_k.......... 3

Kinetic measurements.......... 4

Fluorescence imaging.......... 4

Femetosecond TA spectroscopy.......... 5

UPLC-MS analysis.......... 5

TD-DFT calculations.......... 6

Cell culture.......... 6

Western blotting.......... 6

Assay of the HepG2 cell lysate.......... 7

Flow cytometry.......... 7

Synthesis and Characterization of Compounds.......... 8

Supplementary Tables and Figures.......... 25

Cartesian coordinates (angstrom) for the optimized structures of probes used for P_{+k} or ω_k calculations.......... 66

Cartesian coordinates (angstrom) for the optimized structures of NI9 and NI used for TD-DFT calculations.......... 87

References.......... 89

Materials and Instruments

Unless otherwise stated, all reagents were obtained from commercial source of analytical reagent grade and used without further purification. 96-well black flat bottom polystyrene NBS™ microplate (Corning® Product #3650, USA) with a microplate reader Varioskan Flash (Thermo Fisher Scientific, USA) was used in *in vitro* tests, kinetic measurements and assay in cell lysate samples. GSTs from equine liver (Sigma-Aldrich #G6511, USA) were used for *in vitro* tests. GSTA1-1, GSTM1-1 and GSTP1-1 (Abcam #ab167981, #ab168035 and #ab167990, respectively, UK) were used for enzymatic kinetic study. For all **NI**-series probes, a 4 mM stock solution (dissolved in DMSO) were freshly prepared, followed by dilution with HEPES buffer before use. ^{1}H, ^{13}C and ^{19}F NMR spectra were taken on a nuclear magnetic resonance spectrometer (Bruker, Germany) at room temperature. High resolution mass spectra (HRMS) were recorded on 6540 UHD Accurate-Mass Q-TOF LC/MS instrument (Agilent, USA).

Calculations of spin density distribution, P_k^+, ω and ω_k.

Geometry optimization and vibrational frequency analysis were carried out at the B3LYP/6-31G(d) level[1] of DFT theory. The P_k^+ values were obtained based on natural population analysis (NPA) charge by single-point energy calculations over the optimized neutral geometries using the unrestricted UB3LYP formalism for the radical anion of compounds at the same level, and the results of these calculations were analysed by Multiwfn software[2] to give spin density distributions. Above calculations were conducted with Gaussian 09 suite of programs[3]. The ω values were obtained based on the calculations of vertical ionization potential (I) and vertical electron affinity (A) with ORCA 4.0.1 program at the PWPB95/def2-QZVPP level[4] according to the formulas for the valance state parabola model in the literature[5], utilizing RIJCOSX acceleration with auxiliary basis sets def2/J[6] and def2-QZVPP/C[7] and involving the atom-pairwise dispersion correction with the Becke-Johnson damping scheme (D3BJ)[8]. The integral grid was set to 4, and the convergence limit was set as 'tightSCF'. In light of the analysis in the literature[5] and the probes' applications in biological systems, all these calculations were implemented in gas phase. The ω_k values were calculated according to the Equation (1).

$$\omega_k = \omega \cdot P_k^+ \qquad (1)$$

Kinetic measurements.

Enzymatic kinetic study was performed by monitoring changes in fluorescence intensity with a 96-well black flat bottom polystyrene NBS™ microplate (Corning® Product #3650, USA) using a microplate reader Varioskan Flash (Thermo Fisher Scientific, USA). All measurements were carried out in HEPES buffer (20 mM, <2.5% DMSO, pH 7.4) at 37 °C in duplicate. The final volume of the mixed solution was 200 μL with final enzyme concentration 1.984, 0.992 and 2.500 μg/mL for GSTA1-1, GSTM1-1 and GSTP1-1, respectively. Briefly, a certain probe of a series of concentrations (from 0.39 to 100 μM) was added to the GSH (2 mM, final concentration 1 mM) premixed (10 min earlier) enzyme solution to initiate the reaction. Immediately, a vibration of the reaction system in appropriate strength for 6 s was executed to eliminate the interferences arising from any possible diffusion kinetics. Then the fluorescence emission at 560 nm was measured upon excitation at 445 nm to determine the formation of **NI**. The initial reaction rates were determined by the data within a linear response range, the obvious nonenzymatic reaction noise being subtracted from the enzymatic reaction signal. Kinetic parameters (K_m, k_{cat} and k_{cat}/K_m) were calculated by direct fits of the data to the Michaelis-Menten Equation using a nonlinear regression. For nonenzymatic kinetic study, same measurement conditions were used except the concentrations of probes were ranging from 0 to 6.25 μM, and the second-order rate constant of the nonenzymatic reaction k_{nonc} was determined by direct fits of the data to the equation (2) using a linear regression[9], in which the subscript '0' stands for the initial state of the reaction.

$$v_{0,nonc} = k_{nonc}[GSH]_0[probe]_0 \quad (2)$$

Fluorescence imaging.

Single- and two-photon cellular fluorescence microscopic images were captured by a FV1000 confocal laser scanning microscope (Olympus, Japan) and a FV1000MPE microscope (Olympus, Japan) by exciting the probe with a mode-locked titanium-sapphire pulse laser source (100 fs pulse length, 80 MHz repetition rate, MaiTai Spectra Physics) set at a wavelength of 810 nm, respectively. Direct imaging without washing cells was implemented. For imaging concerned with EA and NEM, operation procedures were as follows: (1) HepG2 cells were pretreated with HEPES buffer (20

mM, 5% glucose, pH 7.4) for 45 min (to rule out interferences from any possible effect induced by long-time incubation in HEPES buffer) and then incubated with 20 μM **NI**-based probe in HEPES buffer for 30 min prior to imaging; (2) HepG2 cells were pretreated with 100 μM EA (a higher concentration will cause considerable cell death) in HEPES buffer for 30 min and then incubated with 20 μM **NI**-based probe in HEPES buffer in the presence of EA for another 30 min prior to imaging; (3) HepG2 cells were pretreated with 50 μM NEM in HEPES buffer for 45 min and then incubated with 20 μM **NI**-based probe in HEPES buffer in the absence of NEM for another 30 min prior to imaging. To address the poor cytomembrane permeability of **NI1**, 0.03% Pluronic-F127 was used. For other probes, cells were never preincubated with any cytomembrane-penetrating reagent.

Femetosecond TA spectroscopy.

TA experiment was carried out using a home-made femtosecond pump-probe setup. Laser pulses (800 nm, 50 fs pulse length, 1 kHz repetition rate) were generated by a Ti: sapphire femtosecond laser source (Spitfire, Spectra-Physics). We used an optical parametric amplifier to change the laser wavelength. The white light-continuum generation from a thin CaF_2 plate was used for the probe. By placing a Berek compensator in the pump beam, the mutual polarization between pump and probe beams was set to the magic angle (54.7°). The wavelength and the power of the pump beam were 370 nm and 80 nJ/pulse, respectively, and the spot size was 0.2 mm in diameter. A solution of *ca.* 2 mM **NI9** in DMSO (Spectrum Pure, >99.9% GC) was subjected to the measurement. The kinetic of the repeated scans remained the same, showing no sign of degradation.

UPLC-MS analysis.

UPLC-MS analysis was performed on a ZORBAX Eclipse Plus C18 column (2.1 × 50 mm, 1.8-Micron, Agilent, USA) using 6540 UHD Accurate-Mass Q-TOF LC/MS instrument (Agilent, USA). LC signal was detected based on absorbance at 370 nm with the conditions: flow rate being 200 μL/min; sample amount being 0.5 μL; eluent ingredient being 5% A to 95% A over the initial 1 min and then maintaining the eluent ingredient for 15 min (solvent A and B are water with 0.1% formic acid and acetonitrile, respectively). The final concentrations of **NI3**, **NI** and GSH were 40 μM, 40 μM and 1 mM, respectively.

TD-DFT calculations.

In order to mimic the real process of photoexcitation in femetosecond TA spectroscopy, the solvent effect in DMSO solution was employed in the SCRF calculations by using the SMD solvation model[10]. Geometrical optimizations of **NI9** and **NI** in the ground state and vibrational frequency analysis were carried out at the B3LYP/6-31G(d) level[1] of DFT method. The electron transitions of **NI9** and **NI** were studied at the B3LYP/aug-cc-pVDZ level[11-13] based on TD-DFT method conducted with Gaussian 16 suite of programs[14].

Cell culture.

HepG2 (human hepatocellular carcinoma cells) and MHCC97L (human hepatocellular carcinoma cells) were obtained from the American Type Culture Collection (ATCC, Manassas, VA, USA). A549 (human breast cancer cells) and HeLa (Human cervical cancer cells) were purchased from Cell Bank of Shanghai Institute of Cell Biology, Chinese Academy of Sciences. HepG2 cells were cultured in Dulbecco's Modified Eagle's Medium (DMEM, Thermo Fisher Scientific, USA) supplemented with 10% fetal bovine serum (FBS, Thermo Fisher Scientific) and 1% penicillin-streptomycin (P/S, Thermo Fisher Scientific). MHCC97L, A549 and Hela cells were cultured in RPMI 1640 medium (Thermo Fisher Scientific) with 10% FBS and 1% P/S. All cells were maintained in a humidified atmosphere containing 5% CO_2 at 37 °C and were seeded in glass bottom dish (φ20 mm, NEST) with a density of 1×10^4 cells per dish for imaging.

Western blotting.

Cells were harvested and lysed with RIPA buffer supplemented with protease and phosphatase inhibitors (Sigma-Aldrich, USA), and then kept on ice for 1 h with vortex every 10 min. Following centrifugation, the supernatant was collected and the protein concentrations were determined by using BCA protein assay kit (Tiangen Biotech Co., Ltd., China). Subsequently, equal amounts of protein added with 3× lithium dodecyl sulfate (LDS) loading buffer were separated by SDS-polyacrylamide gel electrophoresis, and then transferred to polyvinylidene difluoride (PVDF) membranes (Millipore). After blocked with 5% nonfat milk for 2 h, the membranes were incubated with the corresponding primary antibodies overnight at 4 °C, then followed by incubation with HRP-conjugated secondary antibody for 2 h at room temperature. Thereafter, the blots

of interest were incubated with ECL detection reagents (Thermo Fisher Scientific, USA) and visualized by Tanon-5200 chemiluminescent imaging system (Tanon Science & Technology Co., Ltd., China). The primary antibodies including GSTA1 (1:1000, Proteintech #14475-1-AP, USA), GSTM1 (1:1000, Proteintech #12412-1-AP, USA), GSTP1 (1:1000, Proteintech #15902-1-AP, USA) and β-Actin (1:2000, CST, USA) were used in this study with β-Actin served as an internal control.

Assay of the HepG2 cell lysate.

HepG2 cells were harvested and lysed with RIPA buffer, and then kept on ice for 1 h with vortex every 10 min. Following centrifugation, the supernatant was collected and the protein concentrations were determined by using BCA protein assay kit (Tiangen Biotech Co. Ltd, China). This lysate was diluted with HEPES buffer (20 mM, 0.5% DMSO, pH 7.4), making the final concentration of proteins be *ca.* 25 μg/mL. The lysate was preincubated with GSH (2 mM, final assay concentration 1 mM) for 10 min, followed by addition of 20 μM **NI3.** The assay was performed by tracing the change of fluorescence intensity in each sample at 37 °C with NBS™ microplate (Corning® Product #3650, USA) using a microplate reader Varioskan Flash (Thermo Fisher Scientific, USA). The final volume of the mixed solution was 200 μL. The excitation and collected emission wavelengths were 445 and 560 nm respectively. For assay with EA pretreatment, the lysate was preincubated with 200 μM EA for 30 min, followed by addition of GSH (2 mM, final assay concentration 1 mM) for another 10 min, after which 20 μM **NI3** was added. The assay in the control group was simply conducted in the absence of lysate.

Flow cytometry.

FACSCanto™ II (BD, USA) was used for the flow cytometric analysis upon excitation at 488 nm, with fluorescence signal collected at 500−600 nm (using FITC optical filter). HepG2 cells were pretreated with HEPES buffer (20 mM, 0.5% DMSO, 5% glucose, pH 7.4) for 30 min (to rule out interferences from any possible effect induced by long-time incubation in HEPES buffer) and then incubated with 20 μM **NI3** in HEPES buffer for 30 min prior to FACS. For pretreatment with EA, HepG2 cells were preincubated with 100 μM EA (a higher concentration will cause considerable cell death) in HEPES buffer (20 mM, 0.5% DMSO, 5% glucose, pH 7.4) for 30 min, followed by the incubation of 20 μM **NI3** for another 30 min in the presence of EA.

Synthesis and Characterization of Compounds

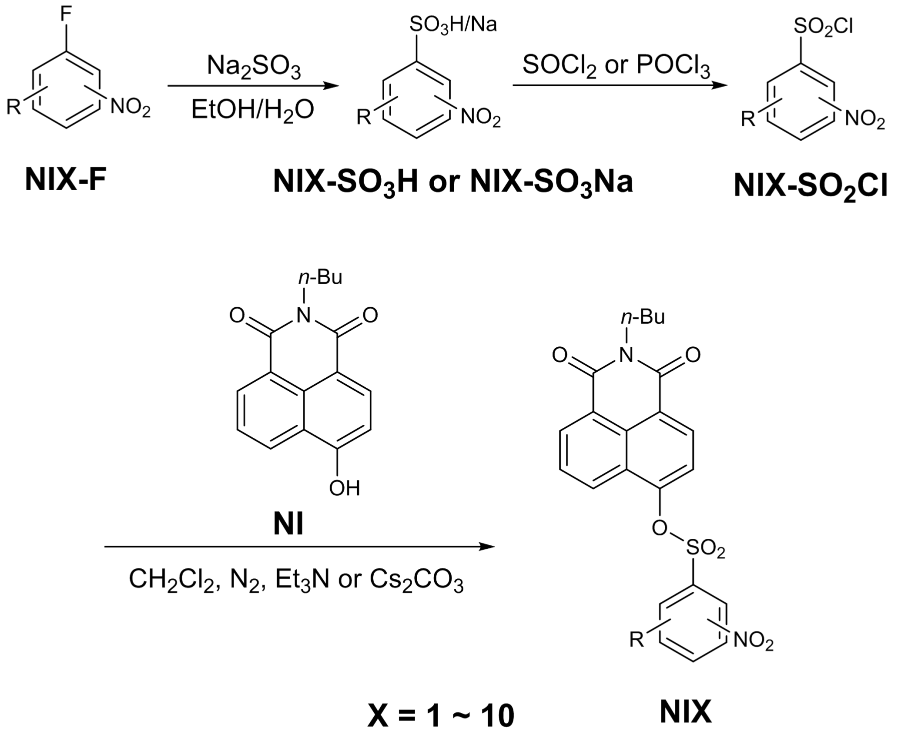

Generally, a sulfonic group was introduced by treating the appropriate nitro-fluorobenzene derivative (**NIX-F**) with sodium sulfite in a solvent mixture of water and ethanol, a modified method adapted from a patent[15], followed by chlorination with thionyl chloride or phosphoryl chloride to give the receptor unit (**NIX-SO$_2$Cl**), which was then readily attached to the hydroxyl of **NI** to prepare the final probe candidate (**NIX**).

NI1

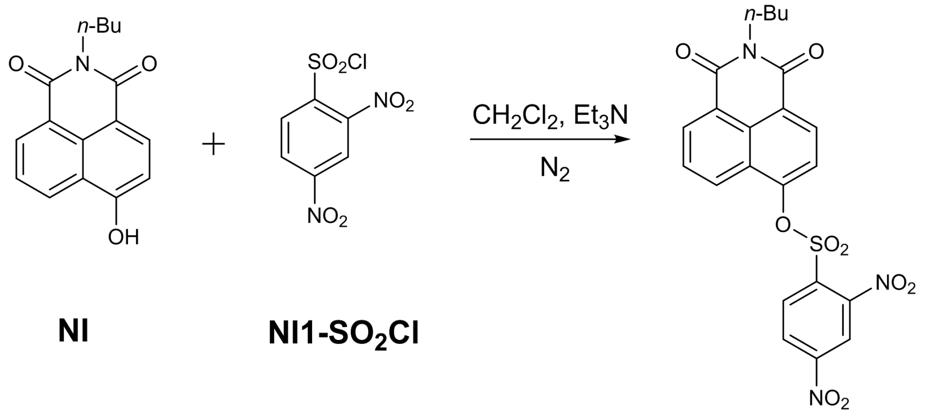

NI **NI1-SO$_2$Cl** **NI1**

NI1-SO$_2$Cl was purchased from the supplier. To a stirred mixture of **NI** (0.27 g, 1 mmol) and **NI1-SO$_2$Cl** (0.4 g, 1.5 mmol) in CH_2Cl_2 (20 mL) being cooled to 0 °C under N_2 was added the triethylamine (0.11 g, 1.1 mmol), after which the ice bath was removed to leave the reaction proceeding until completion. The solvent was evaporated under reduced pressure, and **NI1** as a pale-yellow solid was recrystallized from the resultant crude product with methanol and CH_2Cl_2 (0.27 g, yield 54%). ^{1}H NMR (400 MHz, Chloroform-*d*) δ 8.74 (s, 1H), 8.64 (d, *J* = 7.3 Hz, 1H), 8.57 (dd, *J* = 8.4, 5.0 Hz, 2H), 8.44 (d, *J* = 8.5 Hz, 1H), 8.32 (d, *J* = 8.6 Hz, 1H), 7.81 (t, *J* = 7.9 Hz, 1H), 7.64 (d, *J* = 8.1 Hz, 1H), 4.16 (t, *J* = 7.5 Hz, 2H), 1.75 – 1.65 (m, 2H), 1.51 – 1.39 (m, 2H), 0.98 (t, *J* = 7.3 Hz, 3H). ^{13}C NMR (101 MHz, DMSO) δ 163.43, 162.82, 152.20, 148.82, 148.56, 134.27, 132.14, 131.61, 130.98, 129.24, 129.19, 128.25, 127.78, 125.19, 122.99, 122.44, 121.83, 120.50, 40.59, 29.99, 20.24, 14.17. HRMS (ESI$^+$): $[M+H]^+$, calcd. for $C_{22}H_{18}N_3O_9S^+$, 500.0758; found 500.0754.

NI2-SO$_3$H

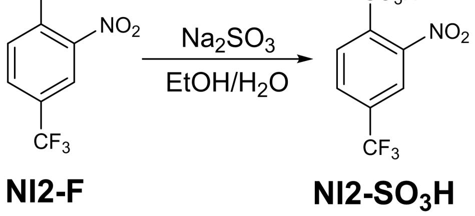

NI2-F **NI2-SO$_3$H**

A suspension of Na_2SO_3 (8.16 g, 64 mmol) in EtOH (70 mL) and water (64 mL) (a little trick: the suspension was formed by addition of EtOH into the aqueous Na_2SO_3 solution quickly) was added to a solution of **NI2-F** (10.8 g, 51.6 mmol) in EtOH (60 mL) and the mixture was heated at 75 °C for 48 h. After cooling, the mixture was acidified to pH = 2 with conc. HCl (4.5 mL) and the solvent was removed *in vacuo*. The residue was diluted with deionized water (130 mL) and the mixture was heated under reflux for 2 h. The solution was cooled in ice bath and the filtrate was collected after filtration, after which the solvent was removed again *in vacuo*. The residue was diluted with excessive methanol, and the filtrate was collected after filtration, after which the solvent was removed to give **NI2-SO₃H** as a pale-yellow solid (10.2 g, yield 73%). ^{1}H NMR (400 MHz, DMSO-d_6) δ 8.17 (d, J = 1.7 Hz, 1H), 8.06 (d, J = 8.2 Hz, 1H), 7.99 (dd, J = 8.3, 1.7 Hz, 1H). ^{13}C NMR (101 MHz, DMSO-d_6) δ 148.19, 143.36, 130.88, 130.65 (q, J = 33.2 Hz), 128.13 (q, J = 3.6 Hz), 123.36 (q, J = 272.7 Hz), 120.51 (q, J = 3.8 Hz). ^{19}F NMR (376 MHz, DMSO-d_6) δ -61.26. HRMS (ESI$^-$): [M-H]$^-$, calcd. for $C_7H_3F_3NO_5S^-$, 269.9690; found 269.9697.

NI2

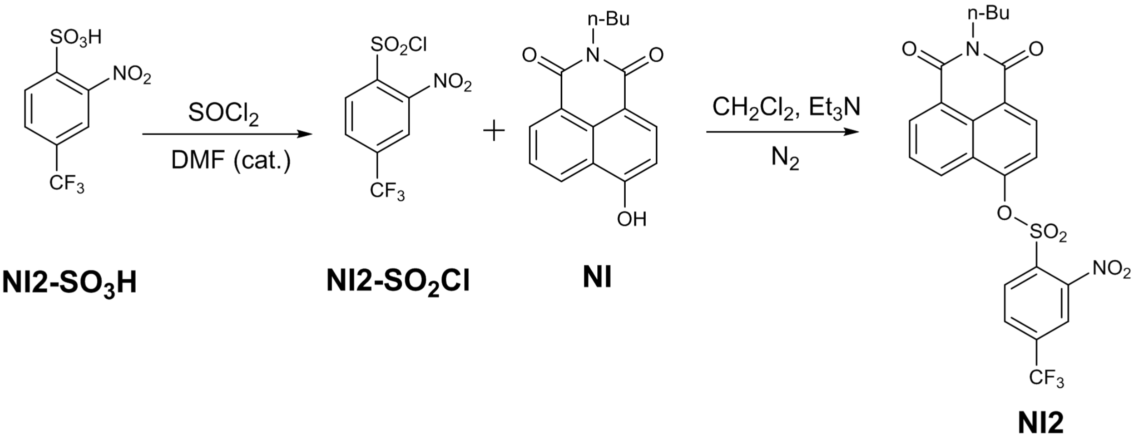

NI2-SO₃H **NI2-SO₂Cl** **NI** **NI2**

To a stirred solution of **NI2-SO₃H** (8 g, 29.5 mmol) in $SOCl_2$ (130 mL) under N_2 was added a few drops of DMF as the catalyst and then the mixture was heated at 80 °C for 10 h. The solvent was removed *in vacuo* and the crude product was chromatographed on silica gel (petroleum ether/ethyl acetate = 500:7) to give **NI2-SO₂Cl** as yellow oil (2.3 g, 27%).

To a stirred mixture of **NI** (0.27 g, 1 mmol) and **NI2-SO$_2$Cl** (0.43 g, 1.5 mmol) in CH_2Cl_2 (20 mL) being cooled to 0 °C under N_2 was added the triethylamine (0.11 g, 1.1 mmol), after which the ice bath was removed to leave the reaction proceeding until completion. The solvent was evaporated under reduced pressure, and **NI2** as a pale-yellow solid was recrystallized from the resultant crude product with methanol and CH_2Cl_2 (0.167 g, yield 32%). ^{1}H NMR (400 MHz, Chloroform-*d*) δ 8.65 (dd, *J* = 7.2, 1.1 Hz, 1H), 8.58 (d, *J* = 8.1 Hz, 1H), 8.45 (dd, *J* = 8.6, 1.1 Hz, 1H), 8.24 (d, *J* = 8.3 Hz, 1H), 8.18 (d, *J* = 1.7 Hz, 1H), 8.01 (dd, *J* = 8.3, 1.7 Hz, 1H), 7.80 (dd, *J* = 8.5, 7.3 Hz, 1H), 7.64 (d, *J* = 8.1 Hz, 1H), 4.20 – 4.14 (m, 2H), 1.76 – 1.65 (m, 2H), 1.44 (h, *J* = 7.4 Hz, 2H), 0.98 (t, *J* = 7.3 Hz, 3H). ^{13}C NMR (101 MHz, Chloroform-*d*) δ 163.60, 162.98, 149.19, 148.84, 137.92 (q, *J* = 35.2 Hz), 132.96, 132.24, 131.81, 131.25, 129.52, 129.37 (q, *J* = 3.5 Hz), 128.19, 127.82, 125.37, 124.34 (q, *J* = 273.1 Hz), 122.94, 122.74 (q, *J* = 3.6 Hz), 122.28, 119.44, 40.44, 30.15, 20.36, 13.82. ^{19}F NMR (376 MHz, Chloroform-*d*) δ -63.40. HRMS (ESI$^+$): $[M+H]^+$, calcd. for $C_{23}H_{18}F_3N_2O_7S^+$, 523.0781; found 523.0800.

NI3-SO$_3$H

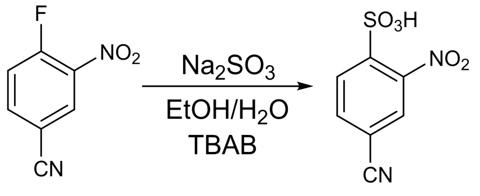

NI3-F **NI3-SO$_3$H**

A suspension of Na_2SO_3 (1.58 g, 12.4 mmol) in EtOH (10 mL) and water (10 mL) (a little trick: the suspension was formed by addition of EtOH into the aqueous Na_2SO_3 solution quickly) was added to a solution of **NI3-F** (1.66 g, 10 mmol) and tetrabutylammonium bromide (TBAB, 0.161 g, 0.5 mmol) in EtOH (50 mL) and the mixture was heated at 30 °C for 16 h. After cooling, the mixture was acidified to pH = 2 with conc. HCl (1.5 mL) and the solvent was removed *in vacuo*. The crude product was chromatographed on silica gel (MeOH/CH_2Cl_2 = 10:500→85:500) to give **NI3-SO$_3$H** as a white solid (1.94 g, yield 85%). ^{1}H NMR (400 MHz, DMSO-*d*$_6$) δ 8.32 (d, *J* = 1.5 Hz, 1H), 8.07 (dd, *J* = 8.1, 1.5 Hz, 1H), 7.99 (d, *J* = 8.1 Hz, 1H). ^{13}C NMR (101 MHz,

DMSO) δ 148.06, 143.72, 135.25, 130.67, 127.07, 117.30, 113.10. HRMS (ESI$^-$): [M-H]$^-$, calcd. for $C_7H_3N_2O_5S^-$, 226.9768; found 226.9771.

NI3

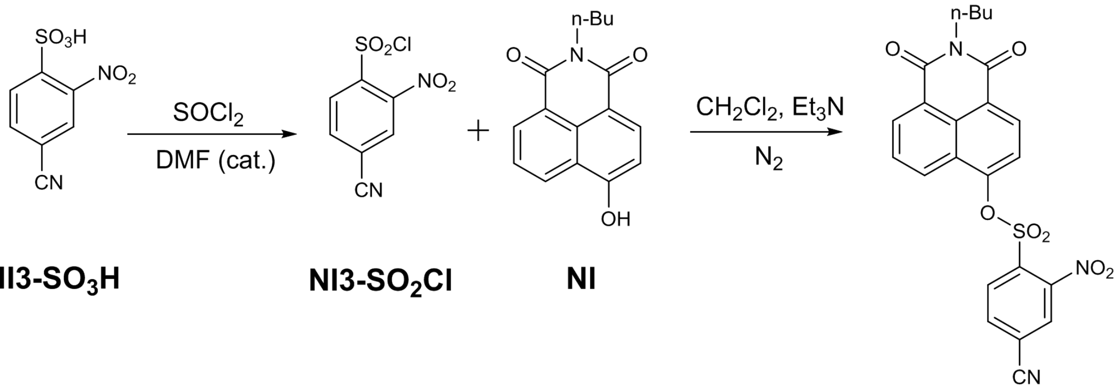

NI3-SO$_3$H **NI3-SO$_2$Cl** **NI** **NI3**

To a stirred solution of **NI3-SO$_3$H** (1.14 g, 5 mmol) in $SOCl_2$ (15 mL) under N_2 was added a few drops of DMF as the catalyst and then the mixture was heated at 80 °C for 4 h. The solvent was removed *in vacuo* and the product was dissolved in CH_2Cl_2 as standby.

To a stirred mixture of **NI** (0.27 g, 1 mmol) and excessive **NI3-SO$_2$Cl** in CH_2Cl_2 (20 mL) being cooled to 0 °C under N_2 was added the triethylamine (0.202 g, 2 mmol), after which the ice bath was removed to leave the reaction proceeding until completion. The solvent was evaporated under reduced pressure, and **NI3** as a brown solid was recrystallized from the resultant crude product with methanol and CH_2Cl_2 (0.122 g, yield 25%). ^{1}H NMR (400 MHz, Chloroform-*d*) δ 8.65 (d, *J* = 7.2 Hz, 1H), 8.57 (d, *J* = 8.1 Hz, 1H), 8.42 (d, *J* = 8.4 Hz, 1H), 8.21 (d, *J* = 8.6 Hz, 2H), 8.02 (dd, *J* = 8.1, 1.5 Hz, 1H), 7.81 (t, *J* = 7.9 Hz, 1H), 7.63 (d, *J* = 8.1 Hz, 1H), 4.17 (t, *J* = 7.6 Hz, 2H), 1.70 (p, *J* = 8.0 Hz, 2H), 1.44 (h, *J* = 7.4 Hz, 2H), 0.98 (t, *J* = 7.3 Hz, 3H). ^{13}C NMR (101 MHz, $CDCl_3$) δ 163.53, 162.92, 149.07, 148.74, 135.76, 132.84, 132.27, 132.23, 131.21, 129.52, 128.50, 128.28, 127.67, 125.29, 122.99, 122.40, 120.03, 119.45, 114.67, 40.45, 30.15, 20.36, 13.82. HRMS (ESI$^+$): [M+H]$^+$, calcd. for $C_{23}H_{18}N_3O_7S^+$, 480.0860; found 480.0839.

NI4-F

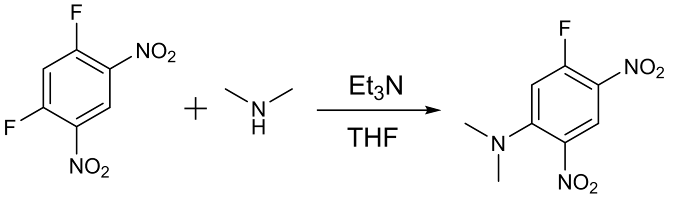

DFDNB **NI4-F**

To a stirred solution of 1,5-difluoro-2,4-dinitro-benzene (**DFDNB**, 6.25 g, 30 mmol) and Et_3N (3.33 g, 33 mmol) in THF (100 mL) was added a solution of dimethylamine (40 wt.% in water, 3.38 g, 30 mmol) in THF (50 mL) dropwise at RT. The mixture was stirred for 1 h and the solvent was removed *in vacuo*. The residue was diluted with water and then let it stand still for 1 h, after which the mixture was filtered to give **NI4-F** as a yellow solid (6.8 g, yield 99%). ^1H NMR (400 MHz, Chloroform-*d*) δ 8.69 (d, J = 8.0 Hz, 1H), 6.74 (d, J = 13.9 Hz, 1H), 3.06 (s, 6H).

NI4-SO_3Na

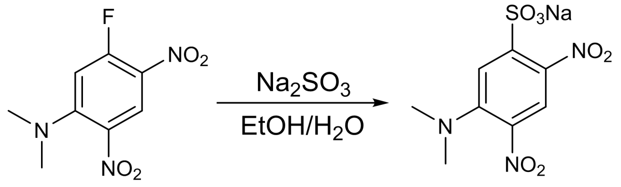

NI4-F **NI4-SO_3Na**

A suspension of Na_2SO_3 (9.48 g, 74.4 mmol) in EtOH (40 mL) and water (80 mL) (a little trick: the suspension was formed by addition of EtOH into the aqueous Na_2SO_3 solution quickly) was added to a solution of **NI4-F** (13.75 g, 60 mmol) in EtOH (120 mL) and the mixture was heated at 80 °C for 48 h. After cooling, the mixture was filtered and the filtrate was concentrated to give **NI4-SO_3Na** as a yellow solid (15.3 g, yield 81%). ^1H NMR (400 MHz, DMSO-d_6) δ 8.21 (s, 1H), 7.57 (s, 1H), 2.94 (s, 6H). ^{13}C NMR (101 MHz, DMSO) δ 146.83, 145.15, 136.97, 134.62, 123.98, 118.10, 42.48. HRMS (ESI$^-$): [M-Na]$^-$, calcd. for $C_8H_8N_3O_7S^-$, 290.0088; found 290.0097.

NI4

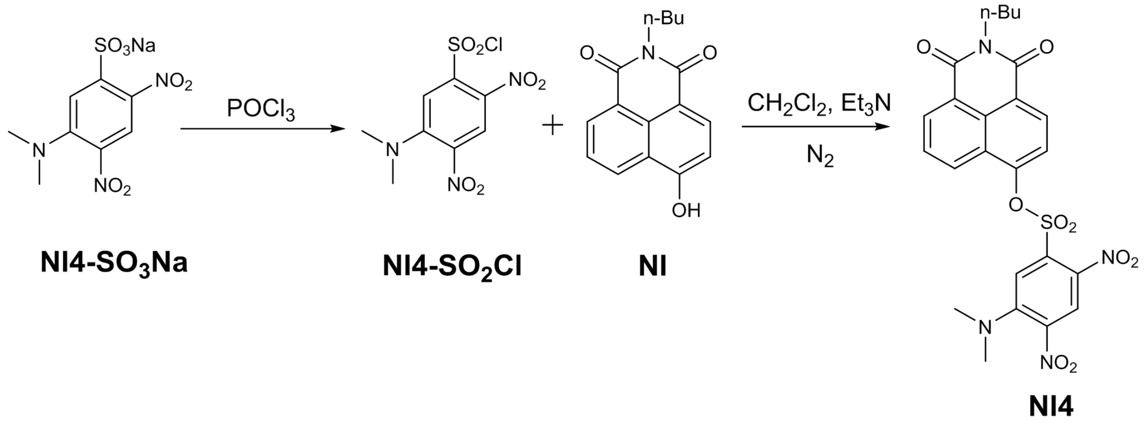

A stirred solution of **NI4-SO_3Na** (1.57 g, 5 mmol) in $POCl_3$ (60 mL) was heated at 146 °C (Caution for such a high temperature! Later repeated experiment verified 105 °C was high enough with a longer reaction time needed) for 40 min and then the solvent was removed *in vacuo*. The residue was diluted with CH_2Cl_2 (30 mL) and the mixture was filtered, after which the filtrate was washed with 5% $NaHCO_3$ (100 mL) to give **NI4-SO_2Cl** as standby.

To a stirred mixture of **NI** (0.27 g, 1 mmol) and excessive **NI4-SO_2Cl** in CH_2Cl_2 (45 mL) being cooled to 0 °C under N_2 was added the triethylamine (0.11 g, 1.1 mmol), after which the ice bath was removed to leave the reaction proceeding until completion. The solvent was evaporated under reduced pressure, and **NI4** as a brown solid was recrystallized from the resultant crude product with methanol and CH_2Cl_2 (0.518 g, yield 96%). ^{1}H NMR (400 MHz, TFA-*d*) δ 8.83 (s, 1H), 8.77 (dd, *J* = 7.4, 1.0 Hz, 1H), 8.71 (d, *J* = 8.3 Hz, 1H), 8.62 (dd, *J* = 8.5, 1.1 Hz, 1H), 8.10 (s, 1H), 7.91 (dd, *J* = 8.5, 7.4 Hz, 1H), 7.79 (d, *J* = 8.2 Hz, 1H), 4.31 – 4.24 (m, 2H), 3.21 (s, 6H), 1.82 – 1.71 (m, 2H), 1.47 (h, *J* = 7.5 Hz, 2H), 0.98 (t, *J* = 7.4 Hz, 3H). ^{13}C NMR (101 MHz, $CDCl_3$) δ 163.65, 163.08, 149.57, 146.62, 136.52, 133.54, 132.20, 131.24, 129.53, 128.19, 128.15, 127.21, 125.49, 122.87, 122.01, 121.50, 119.15, 42.69, 40.41, 30.16, 20.35, 13.81. HRMS (ESI$^+$): $[M+H]^+$, calcd. for $C_{24}H_{23}N_4O_9S^+$, 543.1180; found 543.1184.

NI5-F

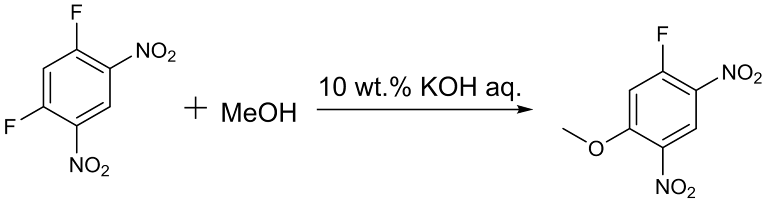

DFDNB **NI5-F**

To a stirred solution of 1,5-difluoro-2,4-dinitro-benzene (**DFDNB**, 6.25 g, 30 mmol) in MeOH (300 mL) was added 10 wt.% KOH aqueous solution (15 mL) dropwise at 0 °C. The ice bath was removed to leave the mixture stirred for 48 h. The mixture was filtered and the filter residue was washed with water and then dried to give **NI5-F** as a pale-yellow solid (5.45 g, yield 84%). ^{1}H NMR (400 MHz, Chloroform-*d*) δ 8.81 (d, J = 7.8 Hz, 1H), 6.99 (d, J = 12.0 Hz, 1H), 4.10 (s, 3H).

NI5-SO$_3$Na

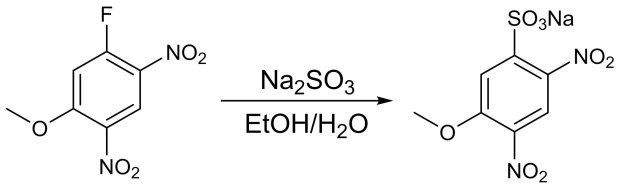

NI5-F **NI5-SO$_3$Na**

A suspension of Na_2SO_3 (3.95 g, 31 mmol) in EtOH (20 mL) and water (35 mL) (a little trick: the suspension was formed by addition of EtOH into the aqueous Na_2SO_3 solution quickly) was added to a solution of **NI5-F** (5.72 g, 26.47 mmol) in EtOH (80 mL) and the mixture was heated at 60 °C for 2 h. After cooling, the mixture was left still until a solid was precipitated out, then a filtration gave **NI5-SO$_3$Na** as a white solid (6.45 g, yield 81%). ^{1}H NMR (400 MHz, DMSO-d_6) δ 8.38 (s, 1H), 7.67 (s, 1H), 4.02 (s, 3H). ^{13}C NMR (101 MHz, DMSO) δ 153.42, 145.78, 140.34, 138.18, 121.31, 114.51, 57.90. HRMS (ESI$^-$): [M-Na]$^-$, calcd. for $C_7H_5N_2O_8S^-$, 276.9772; found 276.9764.

NI5

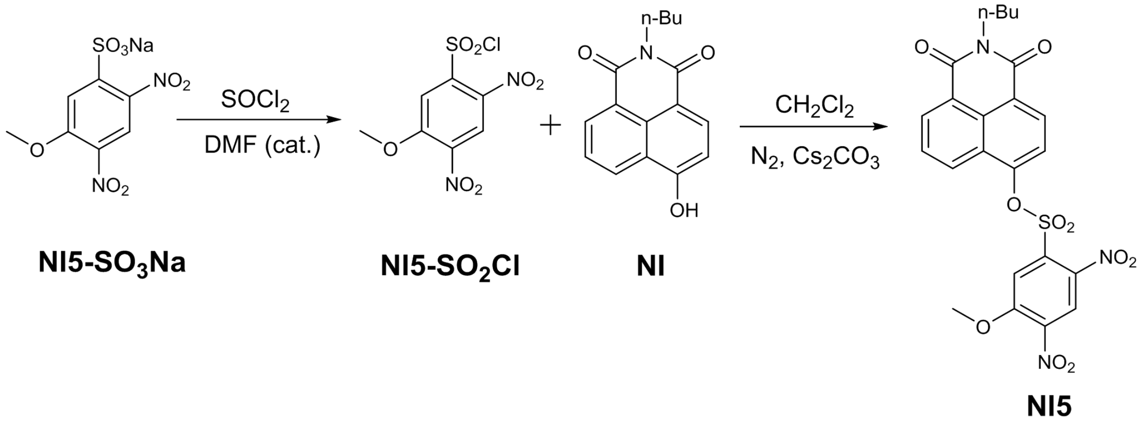

To a stirred solution of **NI5-SO$_3$Na** (3.21 g, 10.7 mmol) in $SOCl_2$ (60 mL) under N_2 was added a few drops of DMF as the catalyst and then the mixture was heated at 80 °C for 4 h. The solvent was removed *in vacuo* and the crude product was chromatographed on silica gel (petroleum ether/ethyl acetate = 500:25→500:45) to give **NI5-SO$_2$Cl** as a pale-yellow solid (1.18 g, yield 37%).

To a stirred mixture of **NI** (0.27 g, 1 mmol) and Cs_2CO_3 (0.18 g, 0.55 mmol) in CH_2Cl_2 (10 mL) being cooled to 0 °C under N_2 was added the solution of **NI5-SO$_2$Cl** (0.326 g, 1.1 mmol) in CH_2Cl_2 (10 mL) dropwise, after which the ice bath was removed to leave the reaction proceeding until completion. The mixture was filtered and the filtrate was collected, followed by evaporation under reduced pressure to remove the solvent. Then **NI5** as a pale-yellow solid was recrystallized from the resultant crude product with methanol and CH_2Cl_2 (0.406 g, yield 77%). ^{1}H NMR (400 MHz, TFA-*d*) δ 8.77 (dd, J = 7.4, 1.0 Hz, 1H), 8.72 (d, J = 8.2 Hz, 1H), 8.63 – 8.59 (m, 2H), 7.98 (s, 1H), 7.91 (dd, J = 8.5, 7.4 Hz, 1H), 7.78 (d, J = 8.2 Hz, 1H), 4.31 – 4.24 (m, 2H), 4.09 (s, 3H), 1.81 – 1.71 (m, 2H), 1.47 (h, J = 7.4 Hz, 2H), 0.98 (t, J = 7.4 Hz, 3H). ^{13}C NMR (101 MHz, $CDCl_3$) δ 163.54, 162.94, 154.87, 149.22, 141.29, 140.27, 134.07, 132.30, 131.17, 129.57, 128.31, 127.83, 125.35, 123.95, 122.99, 122.36, 119.27, 117.30, 58.26, 40.45, 30.15, 20.35, 13.79. HRMS (ESI$^+$): [M+H]$^+$, calcd. for $C_{23}H_{20}N_3O_{10}S^+$, 530.0864; found 530.0871.

NI6-SO_2Cl

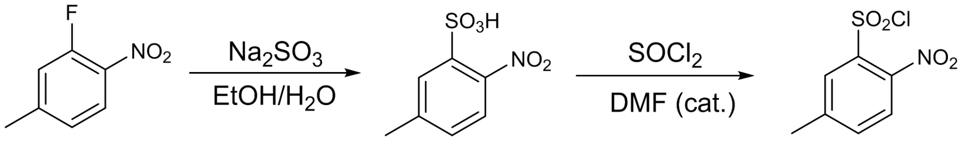

A suspension of Na_2SO_3 (8.16 g, 64 mmol) in EtOH (70 mL) and water (64 mL) (a little trick: the suspension was formed by addition of EtOH into the aqueous Na_2SO_3 solution quickly) was added to a solution of **NI6-F** (8 g, 51.6 mmol) in EtOH (60 mL) and the mixture was heated at 70 °C for 20 h. After cooling, the mixture was acidified to pH = 2 with conc. HCl (4.5 mL) and the solvent was removed *in vacuo*. The residue was diluted with deionized water (130 mL) and the mixture was heated under reflux for 1 h. The solution was cooled in ice bath and the filtrate was collected after filtration, after which the solvent was removed again *in vacuo*. The residue was diluted with excessive methanol, and the filtrate was collected after filtration, after which the solvent was removed to give **NI6-SO_3H** as a pale-yellow solid (8.72 g, yield 72%).

To a stirred solution of **NI6-SO_3H** (3.28 g, 14.94 mmol) in $SOCl_2$ (36 mL) under N_2 was added a few drops of DMF as the catalyst and then the mixture was heated at 80 °C for 3 h. The solvent was removed *in vacuo* and the crude product was chromatographed on silica gel (petroleum ether/ethyl acetate = 500:25) to give **NI6-SO_2Cl** as a white solid (2.5 g, yield 71%). ^1H NMR (400 MHz, Chloroform-*d*) δ 8.05 (d, J = 1.8 Hz, 1H), 7.82 (d, J = 8.1 Hz, 1H), 7.69 (ddd, J = 8.2, 1.8, 0.8 Hz, 1H), 2.59 (s, 3H). ^{13}C NMR (101 MHz, $CDCl_3$) δ 145.07, 144.81, 136.57, 135.95, 130.76, 125.50, 21.55. HRMS (ESI^+): $[M+Na]^+$, calcd. for $C_7H_6ClNNaO_4S^+$, 257.9598; found 257.9598.

NI6

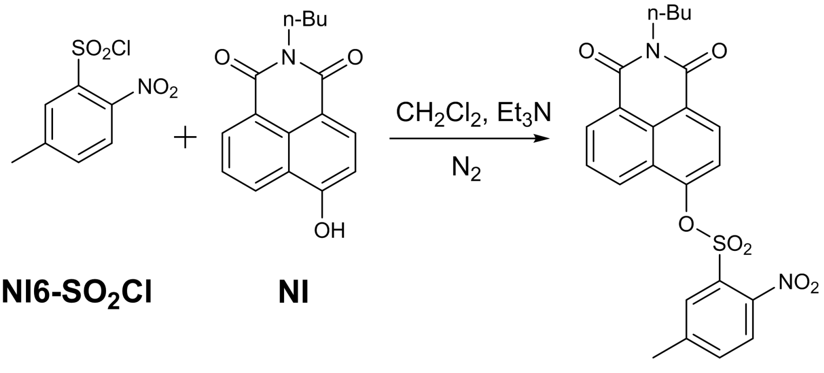

NI6-SO₂Cl **NI** **NI6**

To a stirred mixture of **NI** (0.27 g, 1 mmol) and **NI6-SO_2Cl** (0.36 g, 1.5 mmol) in CH_2Cl_2 (10 mL) being cooled to 0 °C under N_2 was added the triethylamine (0.11 g, 1.1 mmol), after which the ice bath was removed to leave the reaction proceeding until completion. The solvent was evaporated under reduced pressure, and the crude product was chromatographed on neutral Al_2O_3 column (petroleum ether/ethyl acetate = 500:20→500:75) to give **NI6** as a white solid (0.234 g, yield 50%). ^{1}H NMR (400 MHz, DMSO-d_6) δ 8.52 (d, J = 7.3 Hz, 1H), 8.49 (d, J = 8.2 Hz, 1H), 8.38 (d, J = 8.5 Hz, 1H), 8.17 (d, J = 8.2 Hz, 1H), 7.99 (s, 1H), 7.92 (d, J = 8.0 Hz, 2H), 7.68 (d, J = 8.1 Hz, 1H), 4.02 (t, J = 7.4 Hz, 2H), 2.45 (s, 3H), 1.70 – 1.54 (m, 2H), 1.43 – 1.29 (m, 2H), 0.92 (t, J = 7.3 Hz, 3H). ^{13}C NMR (101 MHz, DMSO) δ 167.41, 163.47, 162.88, 149.11, 146.24, 145.25, 137.89, 132.32, 132.01, 131.72, 129.20, 128.88, 127.99, 126.49, 126.29, 125.28, 122.92, 122.06, 120.36, 40.60, 30.01, 21.09, 20.25, 14.17. HRMS (ESI$^+$): $[M+H]^+$, calcd. for $C_{23}H_{21}N_2O_7S^+$, 469.1064; found 469.1083.

NI7-SO_2Cl

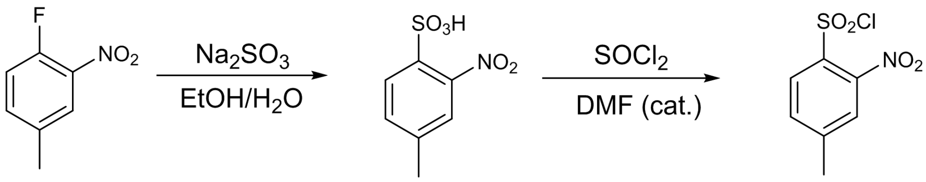

NI7-F **NI7-SO_3H** **NI7-SO_2Cl**

A suspension of Na_2SO_3 (8.16 g, 64 mmol) in EtOH (70 mL) and water (64 mL) (a little trick: the suspension was formed by addition of EtOH into the aqueous Na_2SO_3 solution quickly) was added to a solution of **NI7-F** (8 g, 51.6 mmol) in EtOH (60 mL) and the mixture was heated at 70 °C for 27 h. After cooling, the mixture was acidified to pH = 2 with conc. HCl (4.5 mL) and the solvent was removed *in vacuo*. The residue was diluted with deionized water (130 mL) and the mixture was heated under reflux for 1 h. The solution was cooled in ice bath and the filtrate was collected after filtration, after which the solvent was removed again *in vacuo*. The residue was diluted with excessive methanol, and the filtrate was collected after filtration, after which the solvent was removed to give **NI7-SO₃H** as an orange solid (7.83 g, yield 70%).

To a stirred solution of **NI7-SO_3H** (4.35 g, 19.8 mmol) in $SOCl_2$ (50 mL) under N_2 was added a few drops of DMF as the catalyst and then the mixture was heated at 80 °C for 3 h. The solvent was removed *in vacuo* and the crude product was chromatographed on silica gel (petroleum ether/ethyl acetate = 500:30) to give **NI7-SO_2Cl** as a white solid (3.25 g, yield 70%). ^{1}H NMR (400 MHz, Chloroform-*d*) δ 8.12 (d, *J* = 8.2 Hz, 1H), 7.67 (d, *J* = 1.7 Hz, 1H), 7.62 (ddd, *J* = 8.2, 1.8, 0.9 Hz, 1H), 2.59 (s, 3H). ^{13}C NMR (101 MHz, $CDCl_3$) δ 148.98, 147.20, 133.29, 133.14, 130.38, 125.68, 21.73. HRMS (ESI$^-$): $[M-H]^-$, calcd. for $C_7H_5ClNO_4S^-$, 233.9633; found 233.9625.

<u>**NI7**</u>

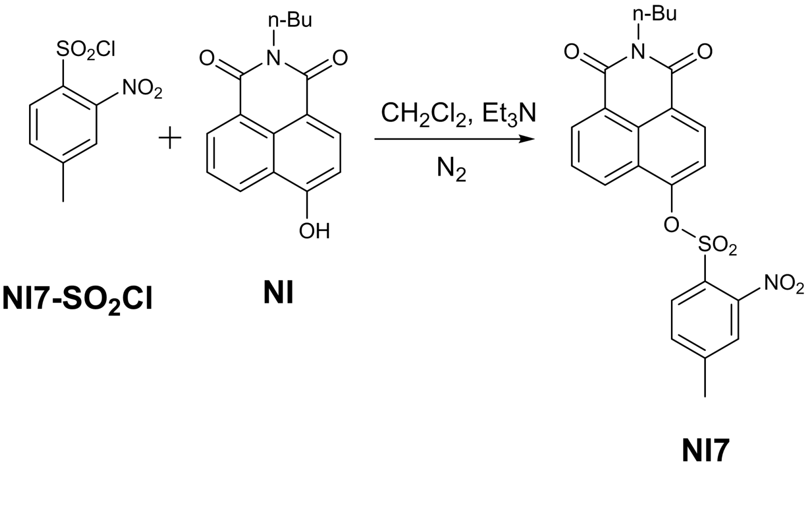

To a stirred mixture of **NI** (0.27 g, 1 mmol) and **NI7-SO_2Cl** (0.36 g, 1.5 mmol) in CH_2Cl_2 (20 mL) being cooled to 0 °C under N_2 was added the triethylamine (0.11 g, 1.1 mmol), after which the ice bath was removed to leave the reaction proceeding until completion. The solvent was evaporated under reduced pressure, and the crude product was chromatographed on neutral Al_2O_3 column (petroleum ether/ethyl acetate = 500:80) to give **NI7** as a white solid (0.300 g, yield 64%). ^{1}H NMR (400 MHz, Chloroform-*d*) δ 8.62 (dd, *J* = 7.3, 1.2 Hz, 1H), 8.55 (d, *J* = 8.1 Hz, 1H), 8.50 (dd, *J* = 8.5, 1.1 Hz, 1H), 7.88 (d, *J* = 8.1 Hz, 1H), 7.77 (dd, *J* = 8.5, 7.3 Hz, 1H), 7.71 (t, *J* = 1.1 Hz, 1H), 7.62 (d, *J* = 8.1 Hz, 1H), 7.49 (ddd, *J* = 8.2, 1.8, 0.9 Hz, 1H), 4.20 – 4.13 (m, 2H), 2.56 (s, 3H), 1.76 – 1.65 (m, 2H), 1.51 – 1.38 (m, 2H), 0.98 (t, *J* = 7.4 Hz, 3H). ^{13}C NMR (101 MHz, $CDCl_3$) δ 163.75, 163.15, 149.66, 148.61, 148.38, 132.78, 132.08, 131.95, 131.32, 129.44, 128.30, 127.90, 125.77, 125.58, 125.26, 122.77, 121.82, 119.50, 40.38, 30.16, 21.68, 20.37, 13.84. HRMS (ESI$^+$): [M+H]$^+$, calcd. for $C_{23}H_{21}N_2O_7S^+$, 469.1064; found 469.1105.

NI8-SO_2Cl

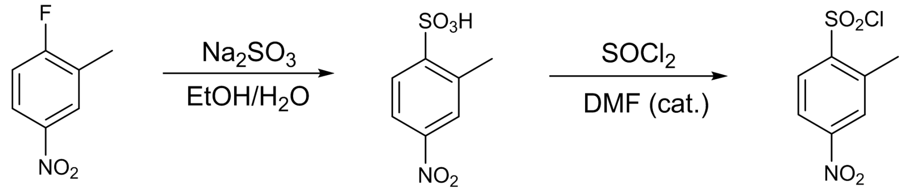

NI8-F **NI8-SO_3H** **NI8-SO_2Cl**

A suspension of Na_2SO_3 (18.56 g, 145.6 mmol) in EtOH (158 mL) and water (145 mL) (a little trick: the suspension was formed by addition of EtOH into the aqueous Na_2SO_3 solution quickly) was added to a solution of **NI8-F** (18.2 g, 117.4 mmol) in EtOH (136 mL) and the mixture was heated at 70 °C for 36 h. After cooling, the mixture was acidified to pH = 2 with conc. HCl (4.5 mL) and the solvent was removed *in vacuo*. The residue was diluted with deionized water (130 mL) and the mixture was heated under reflux for 2 h. The supernatant was collected while the mixture was hot, after which the solvent was removed *in vacuo*. The residue was diluted with excessive methanol, and the filtrate was collected after filtration, after which the solvent was removed to give **NI8-SO_3H** as an orange solid (13.5 g, yield 53%).

To a stirred solution of **NI8-SO_3H** (6.5 g, 29.5 mmol) in $SOCl_2$ (78 mL) under N_2 was added a few drops of DMF as the catalyst and then the mixture was heated at 80 °C for 3 h. The solvent was removed *in vacuo* and the crude product was chromatographed on silica gel (petroleum ether/ethyl acetate = 500:10) to give **NI8-SO_2Cl** as a pale-yellow solid (3.35 g, yield 48%). ^{1}H NMR (400 MHz, Chloroform-*d*) δ 8.31 – 8.27 (m, 2H), 8.27 – 8.23 (m, 1H), 2.92 (s, 3H). ^{13}C NMR (101 MHz, $CDCl_3$) δ 150.92, 147.15, 140.59, 130.28, 128.10, 121.74, 20.65.

<u>NI8</u>

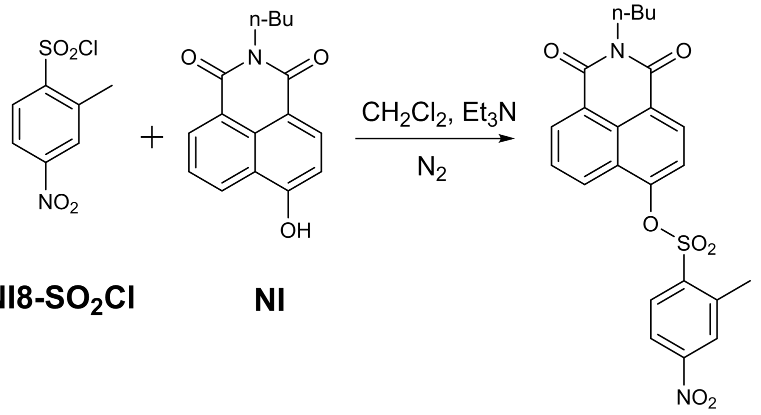

NI8-SO_2Cl **NI** **NI8**

To a stirred mixture of **NI** (0.27 g, 1 mmol) and **NI8-SO_2Cl** (0.36 g, 1.5 mmol) in CH_2Cl_2 (20 mL) being cooled to 0 °C under N_2 was added the triethylamine (0.11 g, 1.1 mmol), after which the ice bath was removed to leave the reaction proceeding until completion. The solvent was evaporated under reduced pressure, and **NI8** as a white solid was recrystallized from the resultant crude product with methanol and CH_2Cl_2 (0.23 g, yield 49%). ^{1}H NMR (400 MHz, Chloroform-*d*) δ 8.66 (d, *J* = 7.3 Hz, 1H), 8.48 (d, *J* = 8.1 Hz, 1H), 8.36 (d, *J* = 10.4 Hz, 2H), 8.20 (d, *J* = 8.9 Hz, 1H), 8.14 (d, *J* = 8.6 Hz, 1H), 7.82 (t, *J* = 7.9 Hz, 1H), 7.27 (s, 1H), 4.16 (t, *J* = 7.6 Hz, 2H), 2.99 (s, 3H), 1.77 – 1.63 (m, 2H), 1.50 – 1.37 (m, 2H), 0.97 (t, *J* = 7.3 Hz, 3H). ^{13}C NMR (101 MHz, $CDCl_3$) δ 163.62, 163.00, 150.99, 149.51, 141.40, 139.67, 132.23, 131.73, 131.19, 129.52, 128.07, 127.81, 127.66, 125.70, 122.97, 121.95, 121.51, 118.66, 77.36, 77.25, 77.04, 76.72, 40.41, 30.15, 21.08, 20.36, 13.83. HRMS (ESI$^+$): $[M+H]^+$, calcd. for $C_{23}H_{21}N_2O_7S^+$, 469.1064; found 469.1068.

NI9

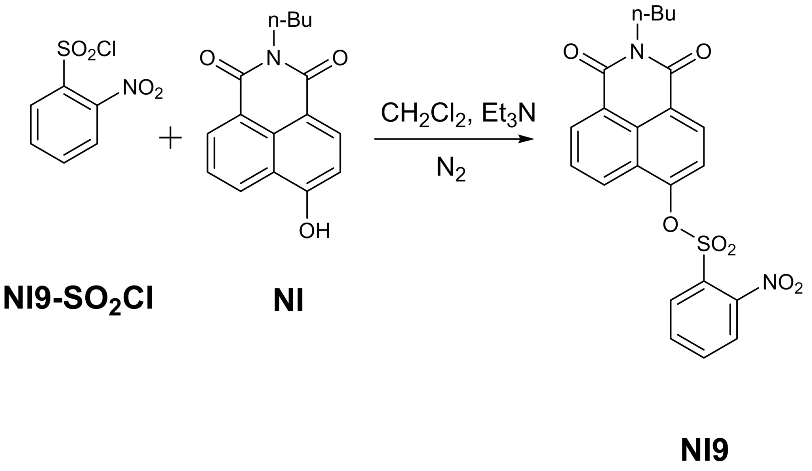

NI9-SO₂Cl was purchased from the supplier. To a stirred mixture of **NI** (0.54 g, 2 mmol) and **NI9-SO₂Cl** (0.66 g, 3 mmol) in CH_2Cl_2 (30 mL) being cooled to 0 °C under N_2 was added the triethylamine (0.22 g, 2.2 mmol), after which the ice bath was removed to leave the reaction proceeding until completion. The solvent was evaporated under reduced pressure, and **NI9** as a pale-yellow solid was recrystallized from the resultant crude product with methanol and CH_2Cl_2 (0.5 g, yield 55%). ^{1}H NMR (400 MHz, Chloroform-*d*) δ 8.62 (dd, *J* = 7.3, 1.1 Hz, 1H), 8.56 (d, *J* = 8.1 Hz, 1H), 8.48 (dd, *J* = 8.4, 1.1 Hz, 1H), 8.03 (dd, *J* = 7.9, 1.2 Hz, 1H), 7.95 – 7.87 (m, 2H), 7.80 – 7.70 (m, 2H), 7.63 (d, *J* = 8.1 Hz, 1H), 4.19 – 4.14 (m, 2H), 1.76 – 1.65 (m, 2H), 1.44 (h, *J* = 7.4 Hz, 2H), 0.98 (t, *J* = 7.3 Hz, 3H). ^{13}C NMR (101 MHz, $CDCl_3$) δ 163.70, 163.10, 149.53, 148.69, 136.08, 132.44, 132.09, 131.96, 131.30, 129.45, 128.29, 128.14, 127.95, 125.53, 125.30, 122.81, 121.95, 119.59, 40.39, 30.16, 20.36, 13.83. HRMS (ESI$^+$): $[M+H]^+$, calcd. for $C_{22}H_{19}N_2O_7S^+$, 455.0907; found 455.0915.

NI10

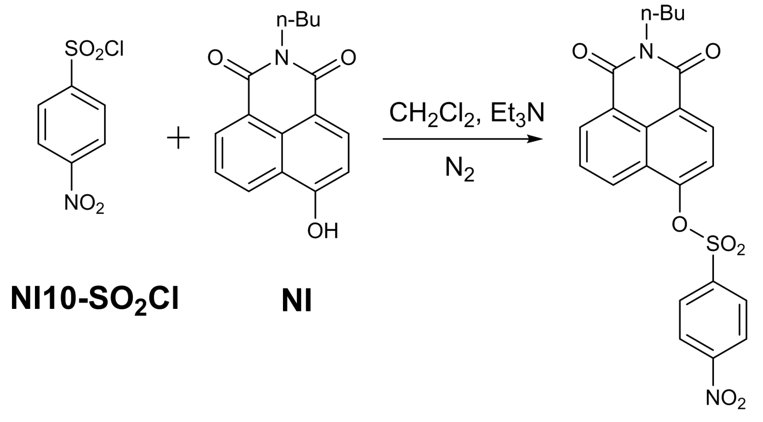

NI10-SO₂Cl **NI** **NI10**

NI10-SO_2Cl was purchased from the supplier. To a stirred mixture of **NI** (0.54 g, 2 mmol) and **NI10-SO_2Cl** (0.66 g, 3 mmol) in CH_2Cl_2 (30 mL) being cooled to 0 °C under N_2 was added the triethylamine (0.22 g, 2.2 mmol), after which the ice bath was removed to leave the reaction proceeding until completion. Let the mixture stand still and then filter residue by filtration was washed with methanol, after which the residue was dried to give **NI10** as a pale-yellow solid (0.7 g, yield 77%). ^1H NMR (400 MHz, TFA-*d*) δ 8.75 (dd, *J* = 7.4, 1.0 Hz, 1H), 8.70 (d, *J* = 8.2 Hz, 1H), 8.48 – 8.43 (m, 2H), 8.39 (dd, *J* = 8.5, 1.0 Hz, 1H), 8.27 – 8.20 (m, 2H), 7.85 (dd, *J* = 8.5, 7.4 Hz, 1H), 7.65 (d, *J* = 8.2 Hz, 1H), 4.33 – 4.19 (m, 2H), 1.83 – 1.67 (m, 2H), 1.47 (h, *J* = 7.4 Hz, 2H), 0.98 (t, *J* = 7.4 Hz, 3H). ^{13}C NMR (101 MHz, TFA-*d*) δ 168.71, 168.00, 153.75, 153.09, 142.59, 136.14, 135.42, 132.13, 131.63, 131.53, 130.56, 127.70, 126.97, 123.69, 122.86, 121.80, 118.55, 115.73, 43.81, 31.70, 21.88, 14.20. HRMS (ESI^+): $[M+H]^+$, calcd. for $C_{22}H_{19}N_2O_7S^+$, 455.0907; found 455.0904.

NI11-F

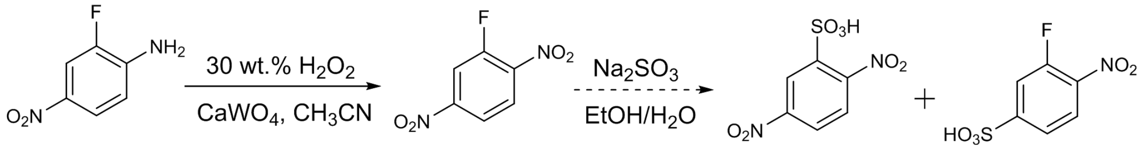

NI11-F **NI11-SO₃H**

In order to try preparing **NI11-SO_3H**, we first synthesized **NI11-F**.

A mixture of 30 wt.% H_2O_2 (43.2 mL) and $CaWO_4$ (1.03g, 3.6 mmol) was stirred at 45 °C until the suspension turned yellow, and then a solution of 2-fluoro-4-nitro-benzenamine (9.48 g, 60 mmol) in CH_3CN (90 mL) was added slowly in 30 min, followed by stirring at 95 °C for 20 h. The organic layer was extracted and the solvent was removed *in vacuo*, resultant crude product being chromatographed on silica gel (petroleum ether/ethyl acetate = 500:20) to give **NI11-F** as a pale-yellow solid (4.14 g, yield 37%). ^{1}H NMR (400 MHz, Chloroform-*d*) δ 8.26 (dd, *J* = 9.2, 6.7 Hz, 1H), 8.23 – 8.18 (m, 2H). ^{13}C NMR (101 MHz, Chloroform-*d*) δ 155.16 (d, *J* = 270.0 Hz), 150.82 (d, *J* = 5.7 Hz), 141.04, 127.29 (d, *J* = 2.5 Hz), 119.68 (d, *J* = 4.8 Hz), 114.63 (d, *J* = 25.4 Hz).

Supplementary Tables and Figures

The global elctrophilicity ω in the valence state parabola model was calculated in terms of vertical ionization potential (I) and vertical electron affinity (A) according to the Equation (3) –(5)[5], in which μ means the electronic chemical potential and η represents the chemical hardness:

$$\mu =-(I+A)/2 \tag{3}$$

$$\eta = (I-A)/2 \tag{4}$$

$$\omega = \mu^2/2\eta = (I+A)^2/4(I-A) \tag{5}$$

Table S1. Electronic chemical potential μ, chemical hardness η, global electrophilicity ω, the electrophilic Parr function P_k^+ of the α-carbon and the local electrophilicity ω_k of the α-carbon for the probe candidates[a]

	μ (eV)	η (eV)	ω (eV)	P_k^+	ω_k (eV)
**NI1**	-5.404	6.390	4.570	0.199	0.909
**NI13**	-5.158	6.558	4.056	0.218	0.882
**NI5**	-5.292	6.541	4.281	0.187	0.800
**NI3**	-5.278	6.606	4.218	0.186	0.785
**NI14**	-5.039	6.775	3.749	0.203	0.761
**NI4**	-5.107	6.699	3.894	0.175	0.680
**NI17**	-4.958	6.756	3.639	0.180	0.653
**NI16**	-4.933	6.685	3.641	0.178	0.648
**NI15**	-4.993	6.869	3.629	0.153	0.557
**NI2**	-5.133	6.777	3.887	0.133	0.517
**NI10**	-5.134	6.754	3.902	0.106	0.414
**NI8**	-5.057	6.928	3.691	0.100	0.369
**NI9**	-4.940	6.928	3.522	0.094	0.331
**NI6**	-4.879	6.956	3.422	0.089	0.304
**NI7**	-4.882	6.938	3.436	0.084	0.290
**NI11**	-5.424	6.261	4.698	0.045	0.210
**NI12**	-5.116	6.677	3.920	-0.037	-0.146

[a] The list was sorted by ω_k from the highest to the lowest. The order of ω_k in **NI2** < **NI3** < **NI1** (0.517 < 0.785 < 0.909) or **NI15** < **NI14** < **NI13** (0.557 < 0.761 < 0.882) is reasonable due to the same order of electron-deficiency in CF_3 < CN < NO_2. The order of ω_k in **NI4** < **NI5** < **NI1** (0.680 < 0.800 < 0.909) or **NI16** < **NI17** < **NI13** (0.648 < 0.653 < 0.882) is also reasonable owing to the order of electron-donating ability in $N(CH_3)_2$ > OCH_3 > H. The order of ω_k in **NI6**, **NI7** < **NI9** (0.304, 0.290 < 0.331) or **NI8** < **NI10** (0.369 < 0.414) is also reasonable because of the order of electron-donating ability in CH_3 > H. All these results demonstrated the rationality in utilizing ω_k to represent the effective reactivity of **NI**-series probes as an electrophile with GSH.

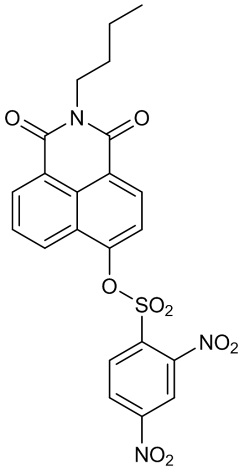

NI1

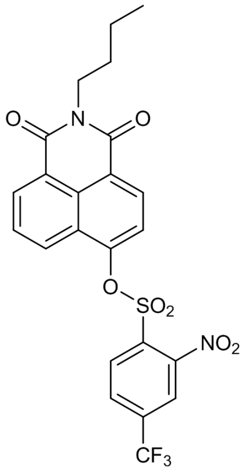

NI2

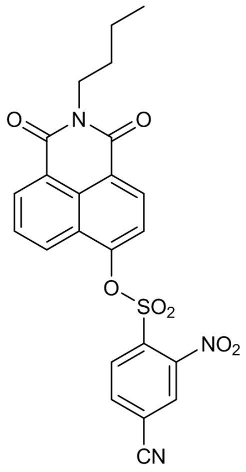

NI3

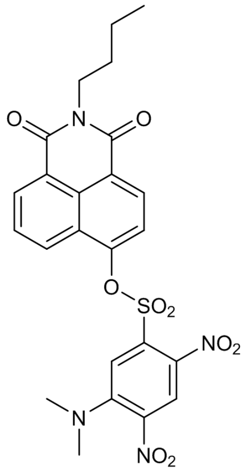

NI4

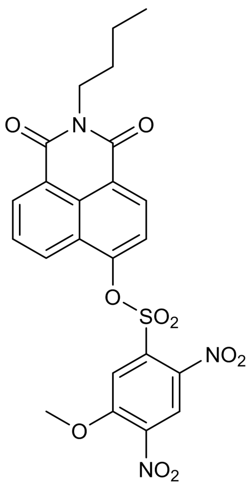

NI5

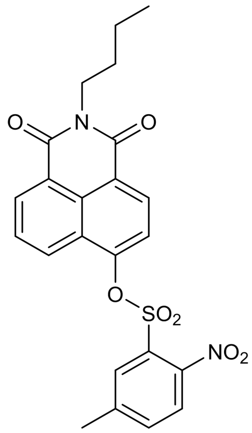

NI6

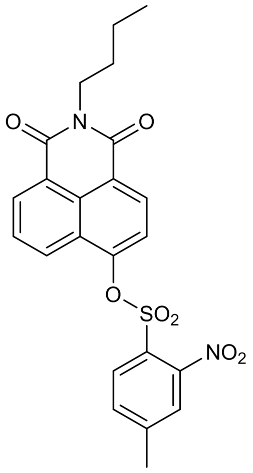

NI7

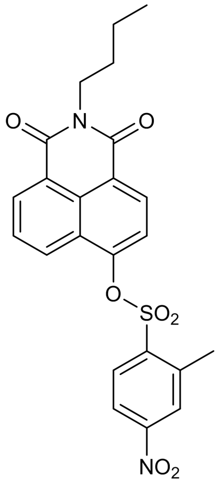

NI8

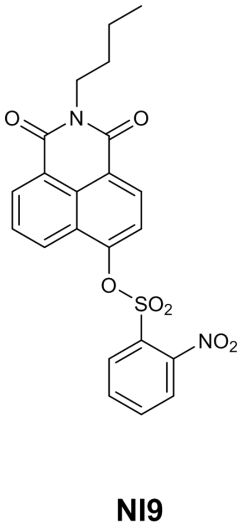

NI9

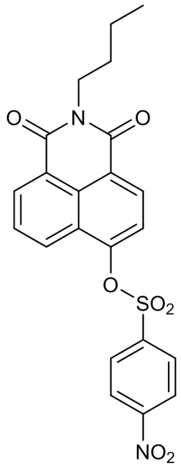

NI10

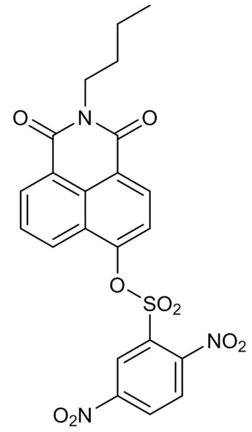

NI11

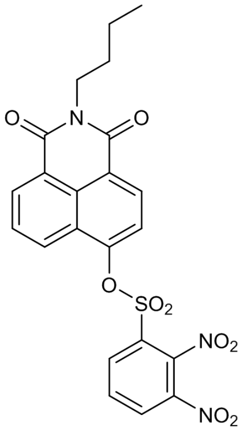

NI12

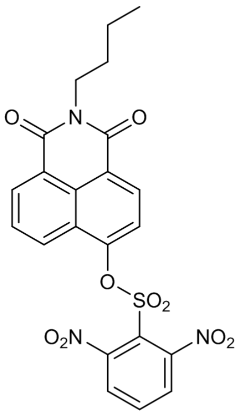

NI13

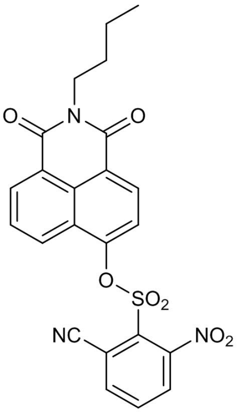

NI14

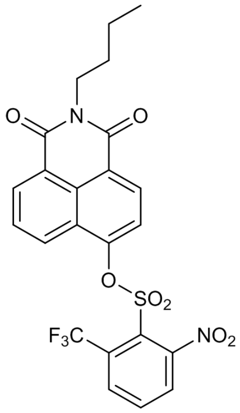

NI15

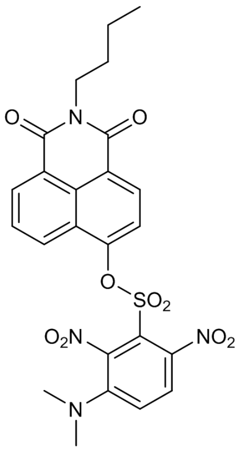

NI16

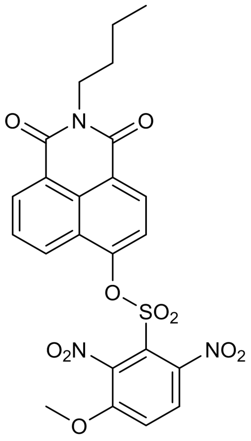

NI17

Table S2. Electronic chemical potential μ, chemical hardness η, global electrophilicity ω, the electrophilic Parr function P_k^+ of the α-carbon and the local electrophilicity ω_k of the α-carbon for **CDNB** and **NNBA**[16].[a]

	μ (eV)	η (eV)	ω (eV)	P_k^+	ω_k (eV)
**CDNB**	-5.920	8.790	3.987	0.252	1.004
**NNBA**	-5.168	6.968	3.834	0.168	0.644

[a] The local electrophilicity ω_k reproduces the stronger nonenzymatic reactivity of **CDNB** as compared with **NNBA** (1.004 > 0.644), in good agreement with the experimental results in this reference[16].

Table S3. Electronic chemical potential μ, chemical hardness η, global electrophilicity ω, the electrophilic Parr function P_k^+ of the α-carbon and the local electrophilicity ω_k of the α-carbon for **ANs-AcRh**, **BNs-AcRh**, **CNs-AcRh** and **DNs-AcRh**[17].[a]

	μ (eV)	η (eV)	ω (eV)	P_k^+	ω_k (eV)
**DNs-AcRh**	-5.154	6.269	4.237	0.240	1.019
**CNs-AcRh**	-5.032	6.493	3.900	0.228	0.891
**ANs-AcRh**	-4.889	6.655	3.591	0.221	0.793
**BNs-AcRh**	-4.815	6.715	3.453	0.217	0.748

[a] The list was sorted by ω_k from the highest to the lowest.

Table S4. Kinetic parameters for enzymatic reactions of probes with GSH catalysed by GSTA1-1[a]

	k_{cat} (s^{-1})	K_m (μM)	k_{cat}/K_m (s^{-1} μM^{-1})
**NI1**	N.D.	N.D.	N.D.
**NI2**	0.158 ± 0.009	4.1 ± 0.8	0.039 ± 0.006
**NI3**	0.673 ± 0.028	6.5 ± 0.9	0.103 ± 0.011
**NI4**	0.052 ± 0.002	3.3 ± 0.4	0.016 ± 0.002
**NI5**	N.D.	N.D.	N.D.
**NI6**	0.016 ± 0.001	3.3 ± 0.6	0.005 ± 0.001
**NI7**	0.005 ± 0.000	0.9 ± 0.1	0.006 ± 0.001
**NI8**	0.004 ± 0.000	0.4 ± 0.0	0.009 ± 0.001
**NI9**	0.010 ± 0.001	2.5 ± 0.6	0.004 ± 0.001
**NI10**	0.012 ± 0.001	2.2 ± 0.8	0.006 ± 0.002

[a] N.D. = Not determined. Data for **NI1** and **NI5** were unobtainable due to the relatively too large background noise.

Table S5. Kinetic parameters for enzymatic reactions of probes with GSH catalysed by GSTM1-1[a]

	k_{cat} (s^{-1})	K_m (μM)	k_{cat}/K_m (s^{-1} μM^{-1})
**NI1**	1.656 ± 0.150	16.4 ± 4.4	0.101 ± 0.020
**NI2**	0.524 ± 0.016	12.9 ± 1.2	0.041 ± 0.003
**NI3**	1.072 ± 0.073	5.5 ± 1.4	0.196 ± 0.042
**NI4**	0.587 ± 0.028	37.9 ± 4.1	0.016 ± 0.001
**NI5**	N.D.	N.D.	N.D.
**NI6**	0.012 ± 0.001	3.5 ± 0.6	0.003 ± 0.000
**NI7**	0.001 ± 0.000	0.4 ± 0.2	0.001 ± 0.000
**NI8**	0.003 ± 0.000	0.4 ± 0.1	0.007 ± 0.001
**NI9**	0.017 ± 0.001	1.5 ± 0.3	0.012 ± 0.002
**NI10**	0.003 ± 0.000	0.8 ± 0.2	0.004 ± 0.001

[a] N.D. = Not determined. Data for **NI5** were unobtainable due to the relatively too large background noise.

Table S6. Kinetic parameters for enzymatic reactions of probes with GSH catalysed by GSTP1-1[a]

	k_{cat} (s^{-1})	K_m (μM)	k_{cat}/K_m (s^{-1} μM^{-1})
**NI1**	N.D.	N.D.	N.D.
**NI2**	0.008 ± 0.000	6.6 ± 0.9	0.001 ± 0.000
**NI3**	0.175 ± 0.011	5.7 ± 1.3	0.031 ± 0.006
**NI4**	0.059 ± 0.001	19.5 ± 1.1	0.003 ± 0.000
**NI5**	N.D.	N.D.	N.D.
**NI6**	UD	UD	UD
**NI7**	UD	UD	UD
**NI8**	UD	UD	UD
**NI9**	UD	UD	UD
**NI10**	UD	UD	UD

[a] N.D. = Not determined. UD = undetectable. Data for **NI1** and **NI5** were unobtainable due to the relatively too large background noise. Data for **NI6**−**NI10** were undetectable due to their low sensitivity to this specific isoenzyme, in agreement with previous similar results that substrates showed less sensitivity for GSTP than for GSTA or GSTM[17,18].

Table S7. Quantitative comparison of the probes in terms of sensitivity and S/N ratio[a]

	GSTA1-1		GSTM1-1		GSTP1-1	
	k_{cat} (s^{-1})	k_{cat}/k_{nonc} (M)	k_{cat} (s^{-1})	k_{cat}/k_{nonc} (M)	k_{cat} (s^{-1})	k_{cat}/k_{nonc} (M)
**NI1**	/	/	1.656 ± 0.150	0.55 ± 0.07	/	/
**NI2**	0.158 ± 0.009	6.39 ± 0.87	0.524 ± 0.016	21.15 ± 2.34	0.008 ± 0.000	0.32 ± 0.02
**NI3**	0.673 ± 0.028	0.73 ± 0.15	1.072 ± 0.073	1.16 ± 0.27	0.175 ± 0.011	0.19 ± 0.04
**NI4**	0.052 ± 0.002	0.21 ± 0.04	0.587 ± 0.028	2.37 ± 0.49	0.059 ± 0.001	0.24 ± 0.05

[a] Note the unit of k_{cat}/k_{nonc} is M, namely mol/L. This is due to the different units of k_{cat} and k_{nonc} (s^{-1} and s^{-1} M^{-1}, respectively), so here the S/N ratio is merely described by k_{cat}/k_{nonc}, and the definite S/N ratio should take the concentrations of the enzyme, probes and GSH all into account.

Table S8. Respective mean fluorescence intensity (FI) in flow cytometry analysis regarding **NI3**.

	Mean FI
HepG2 (Control)	709
HepG2 + EA	693
HepG2 + **NI3**	7542
HepG2 + EA + **NI3**	3316

Table S9. Calculated electronic transitions of **NI9** and **NI** at TD-DFT/B3LYP/aug-cc-pVDZ level with the smd solvation model (solvent: DMSO)

Transitions	λ_{cal} (nm)	λ_{exp} (nm)	f	CI expansion coefficients
**NI9**				
$S_0 \rightarrow S_1$	409		0.009	0.706 (H→L)
$S_0 \rightarrow S_2$	356	360	0.382	0.700 (H→L+1)
**NI**				
$S_0 \rightarrow S_1$	455	445	0.200	0.697 (H→L)
$S_0 \rightarrow S_2$	364		0.000	0.696 (H-1→L)

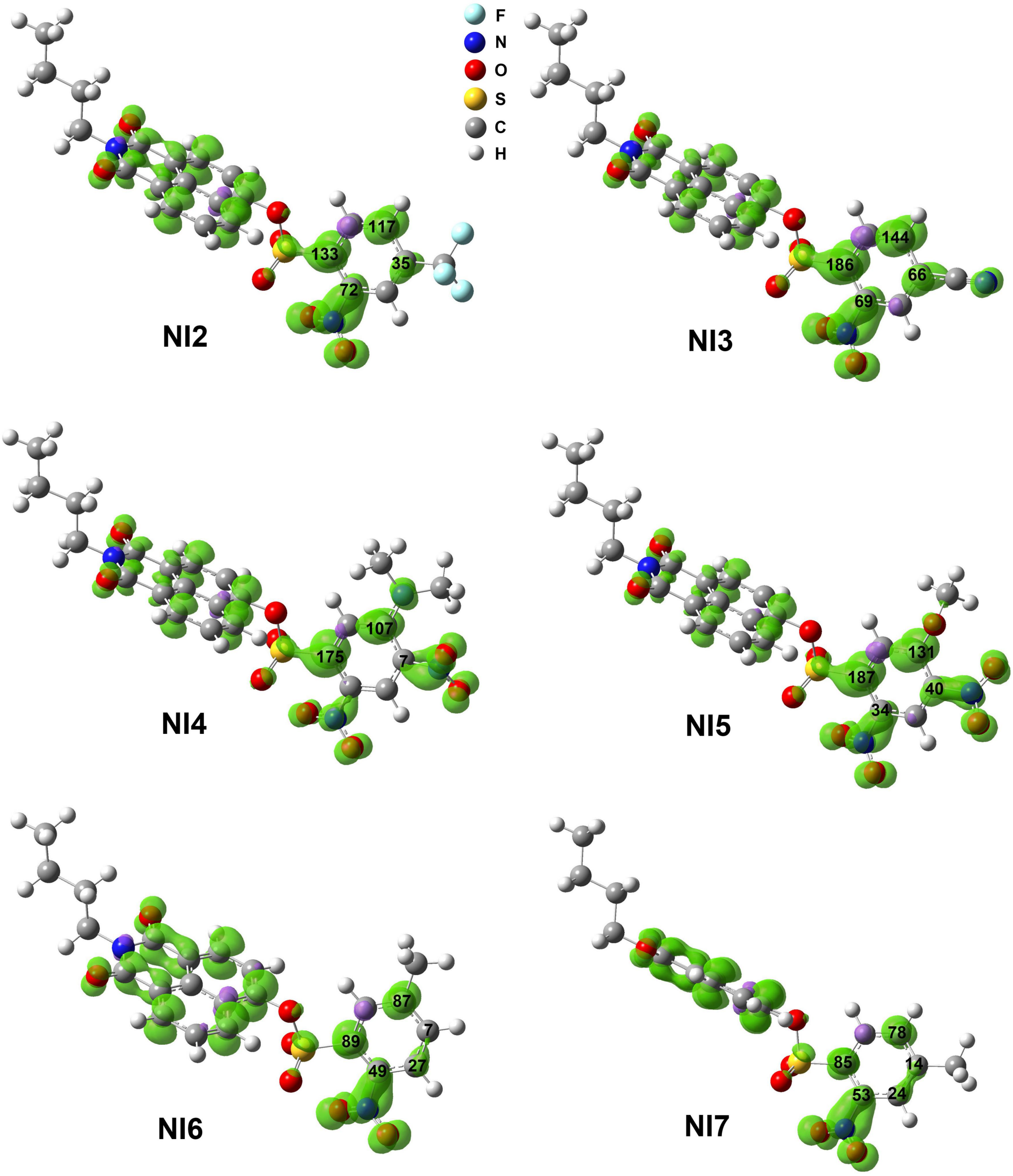
F
N
O
S
C
H
117
133
35
72
NI2
144
186
66
69
NI3
107
175
7
NI4
131
187
40
34
NI5
87
89
7
49
27
NI6
78
85
14
53
24
NI7

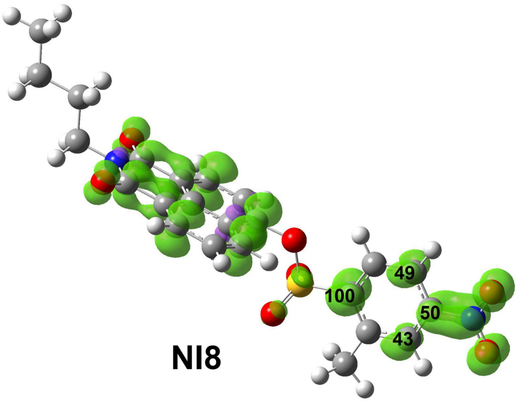

NI8

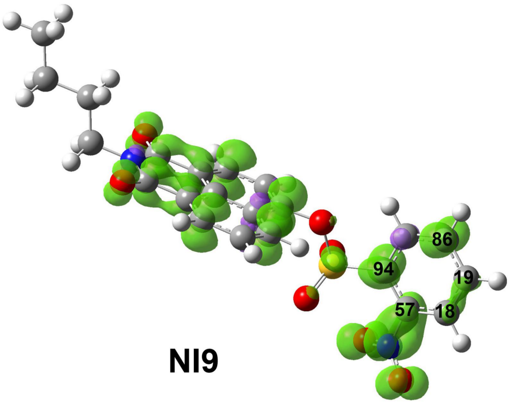

NI9

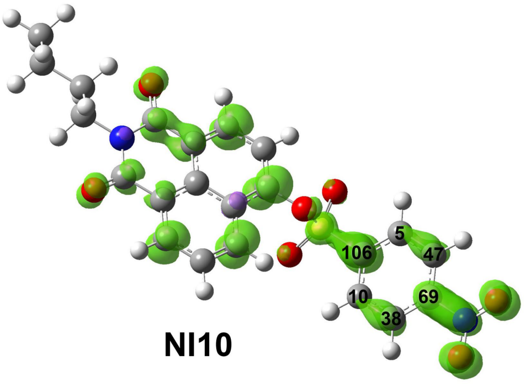

NI10

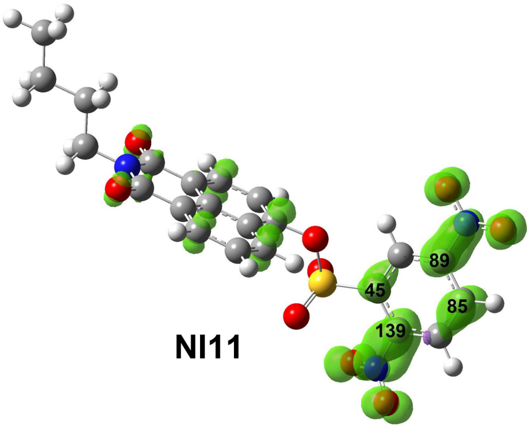

NI11

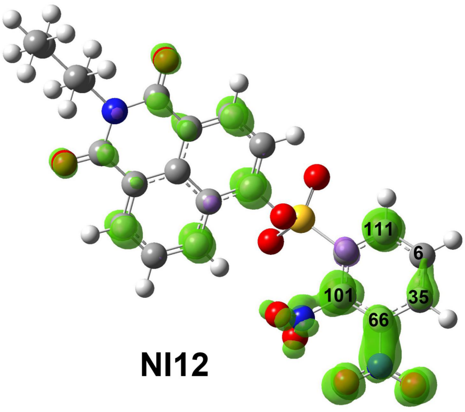

NI12

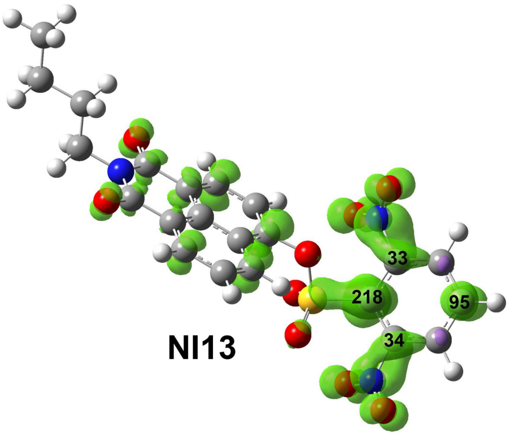

NI13

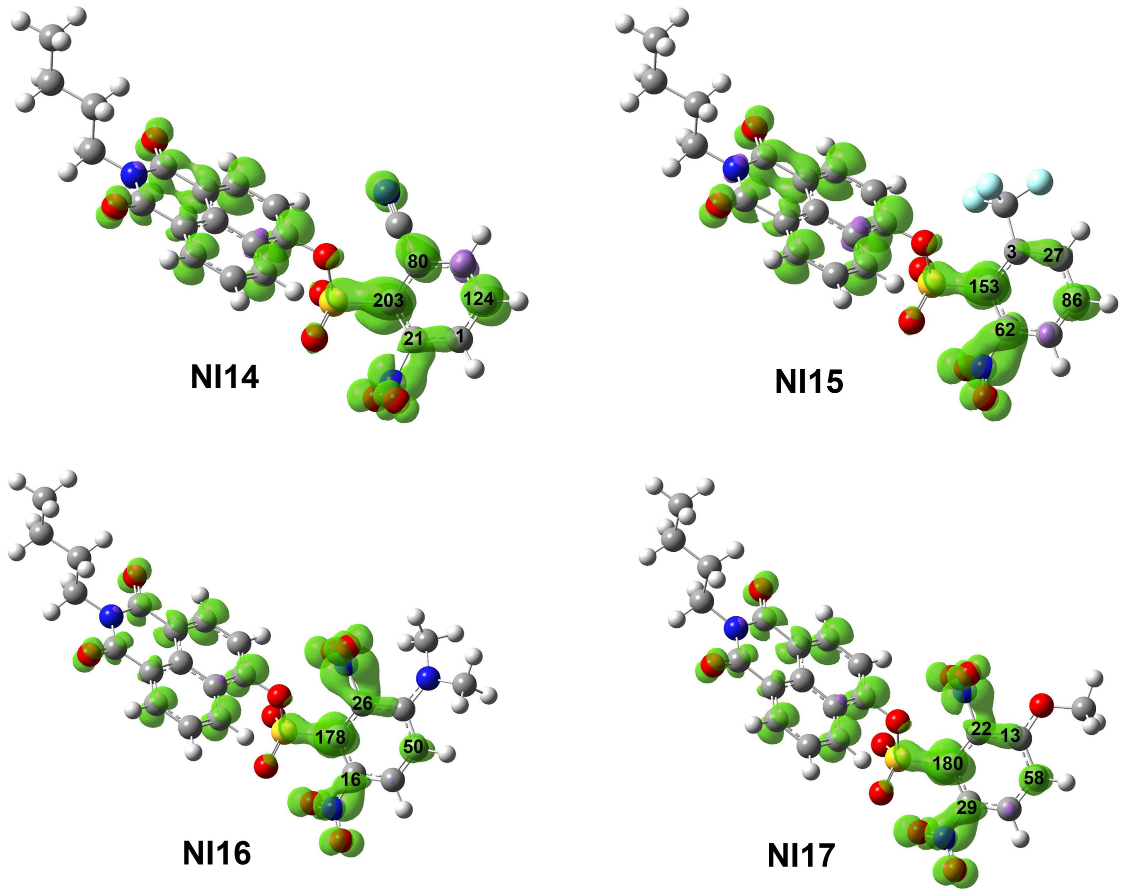

Figure S1. Spin density distributions of the anion radicals of probe candidates **NI2−NI17**. Respective positive P_k^+ value (amplified by a factor of 1,000) of the carbon atoms in the nitrobenzene ring is marked, with positive and negative spin density coloured by green and purple, respectively. Negative spin density or P_k^+ values are herein regarded as meaningless.[19,20] Isodensity value = 0.002.

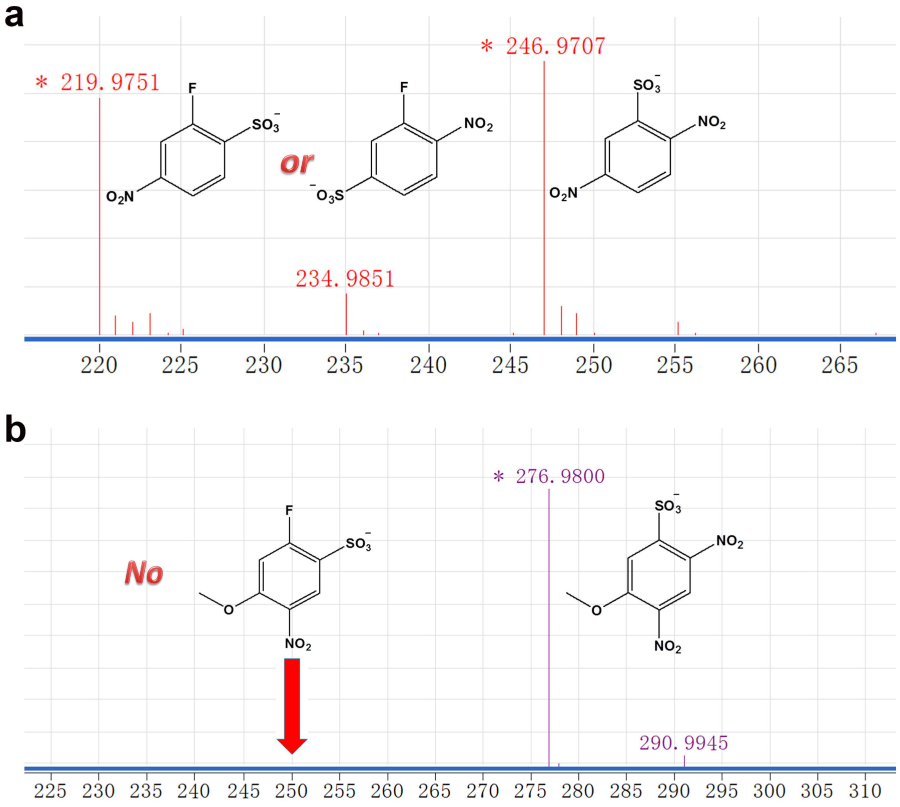

Figure S2. Comparison of the mass spectra of the reaction mixture of on-going synthesis of (**a**) 2,5-dinitro-benzenesulfonic acid or (**b**) 5-methoxy-2,4-dinitro-benzenesulfonic acid (i.e. intermediate product **NI5-SO$_3$H**). **a**, Apart from the target product ([M-H]$^-$, calcd. for $C_6H_3N_2O_7S^-$, 246.9666; found 246.9707), a nitro-substituted compound, namely 3-fluoro-4-nitro-benzenesulfonic acid or 2-fluoro-4-nitro-benzenesulfonic acid ([M-H]$^-$, calcd. for $C_6H_3FNO_5S^-$, 219.9721; found 219.9751) was also found. **b**, Only the target product ([M-H]$^-$, calcd. for $C_7H_5N_2O_8S^-$, 276.9772; found 276.9800) emerged, with no nitro-substituted product 2-fluoro-4-methoxy-5-nitro-benzenesulfonic acid found ([M-H]$^-$, calcd. for $C_7H_5FNO_6S^-$, 249.9827). Please refer to the corresponding synthesis section (**NI11-F**).

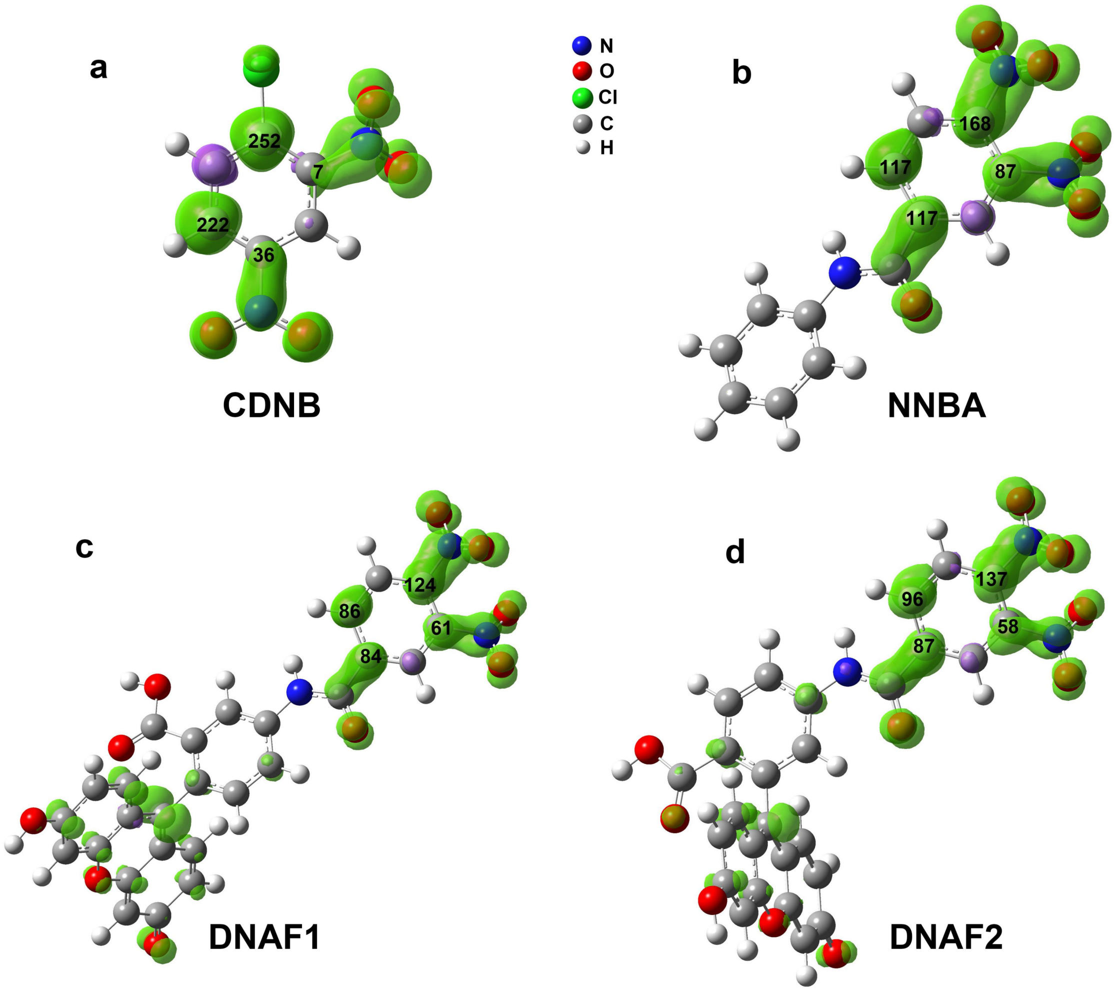

Figure S3. Spin density distribution of the anion radicals of (**a**) **CDNB**, (**b**) **NNBA**, (**c**) **DNAF1** and (**d**) **DNAF2**[16]. Respective positive P_k^+ value (amplified by a factor of 1,000) of the carbon atoms in the nitrobenzene ring is marked, the α-carbon of the aryl-sulfonyl group showing the maximum spin density and P_k^+, with positive and negative spin density coloured by green and purple, respectively. Negative spin density or P_k^+ values are herein regarded as meaningless.[19,20] Isodensity value = 0.002.

The electrophilic Parr function P_k^+ reproduces quite well the regioselectivity of various substrates for GST, whose chemical structures are distinct from those of naphthalimide-based probes reported in this work, demonstrating the universality of this parameter. Particularly, for the adjacent two nitro groups in **NNBA**, **DNAF1** and **DNAF2**, this parameter describes the true one that is substituted by GSH (0.168 > 0.087 for **NNBA**, 0.124 > 0.061 for **DNAF1**, 0.137 > 0.058 for **DNAF2**), which was proved by ^{1}H-NMR chemical shifts in this reference[16].

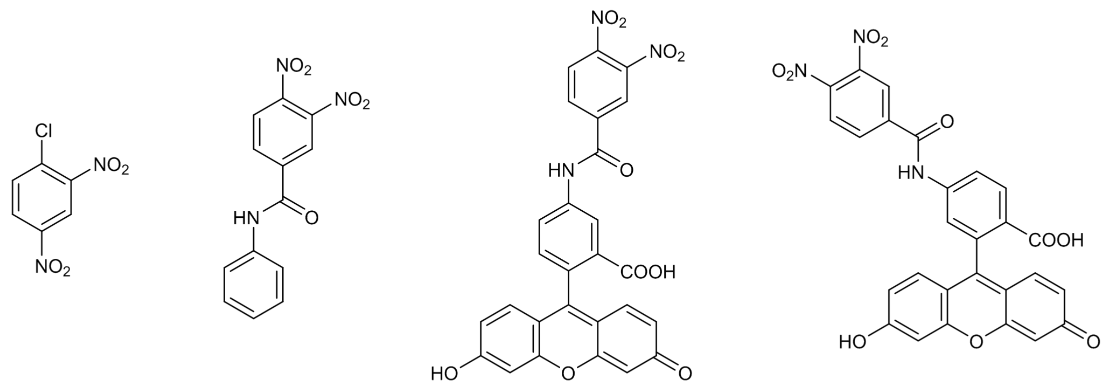

CDNB NNBA DNAF1 DNAF2

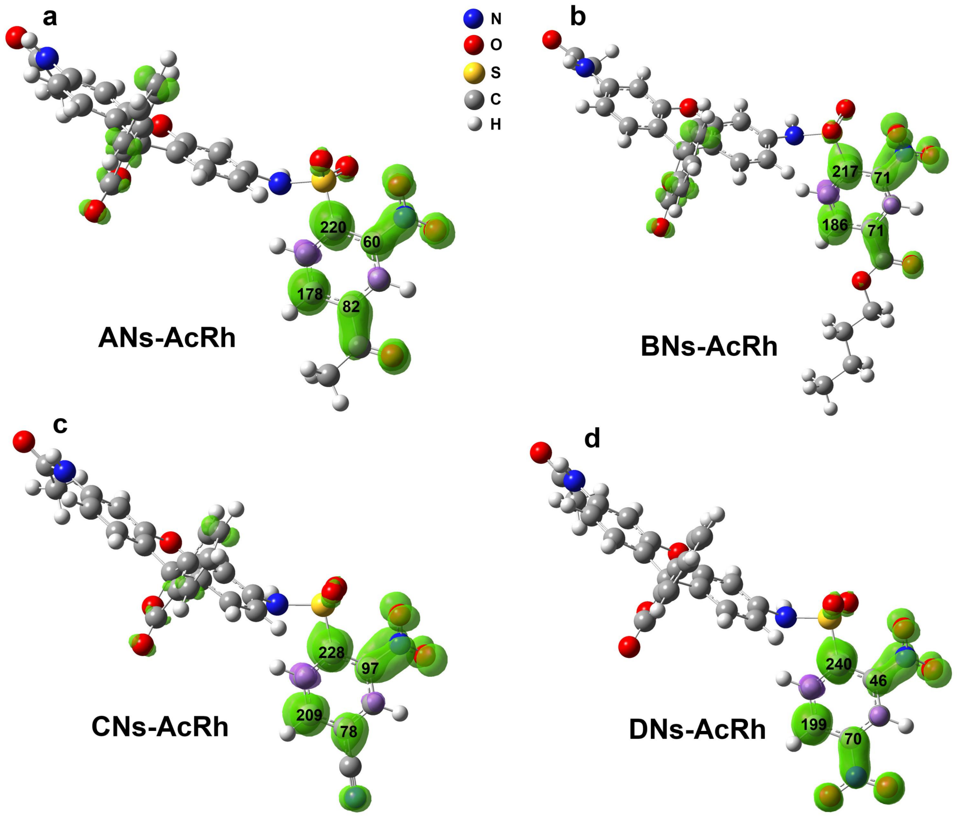

Figure S4. Spin density distribution of the anion radicals of (**a**) **ANs-AcRh**, (**b**) **BNs-AcRh**, (**c**) **CNs-AcRh** and (**d**) **DNs-AcRh**[17]. Respective positive P_k^+ value (amplified by a factor of 1,000) of the carbon atoms in the nitrobenzene ring is marked, the α-carbon of the arylsulfonyl group showing the maximum spin density and P_k^+, with positive and negative spin density coloured by green and purple, respectively. Negative spin density or P_k^+ values are herein regarded as meaningless.[19,20] Isodensity value = 0.002.

The electrophilic Parr function P_k^+ reproduces quite well the regioselectivity of probes reported therein[17]. Note the difference between the sulfonamide group in these probes and sulfonyl group in our probes.

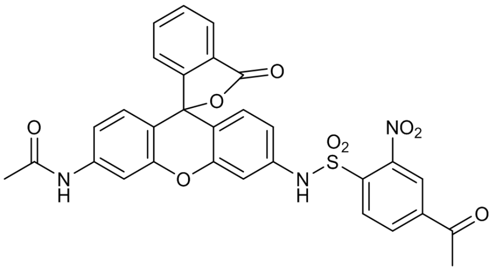

ANs-AcRh

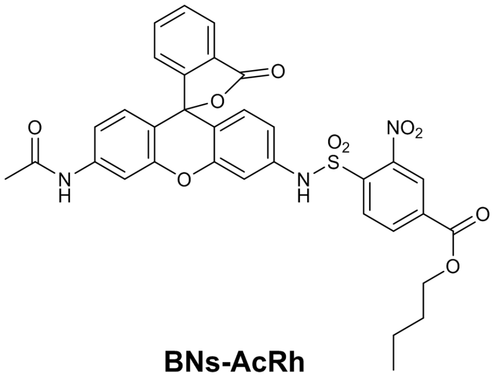

BNs-AcRh

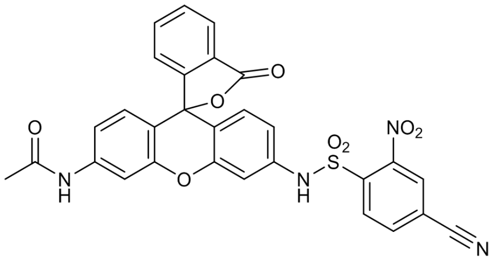

CNs-AcRh

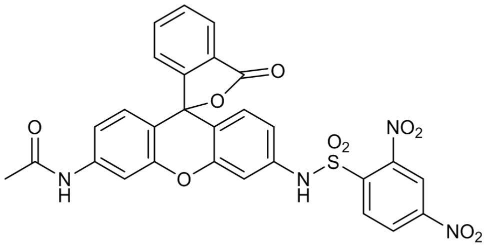

DNs-AcRh

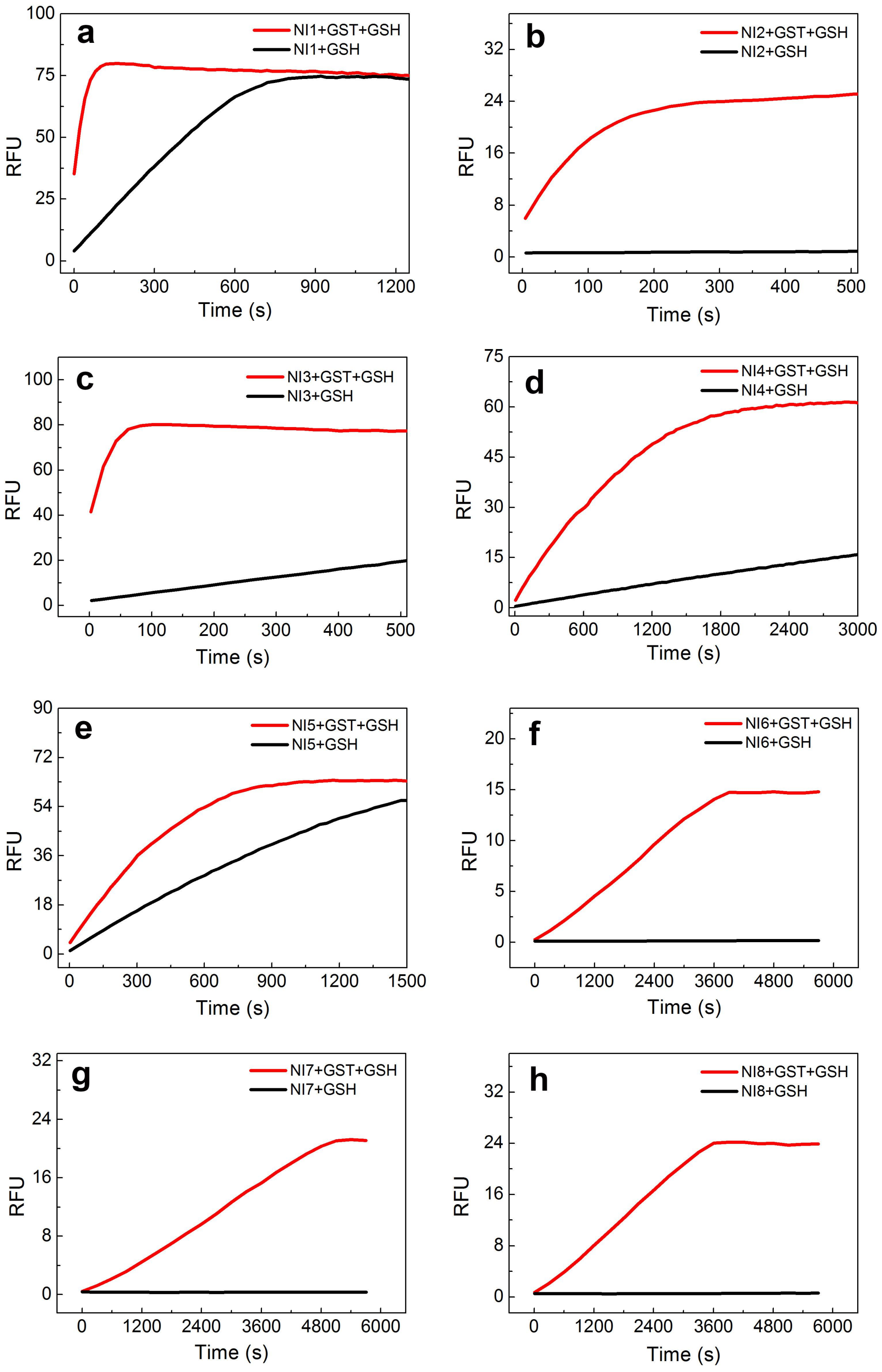
a
NI1+GST+GSH
NI1+GSH
RFU
Time (s)
b
NI2+GST+GSH
NI2+GSH
RFU
Time (s)
c
NI3+GST+GSH
NI3+GSH
RFU
Time (s)
d
NI4+GST+GSH
NI4+GSH
RFU
Time (s)
e
NI5+GST+GSH
NI5+GSH
RFU
Time (s)
f
NI6+GST+GSH
NI6+GSH
RFU
Time (s)
g
NI7+GST+GSH
NI7+GSH
RFU
Time (s)
h
NI8+GST+GSH
NI8+GSH
RFU
Time (s)

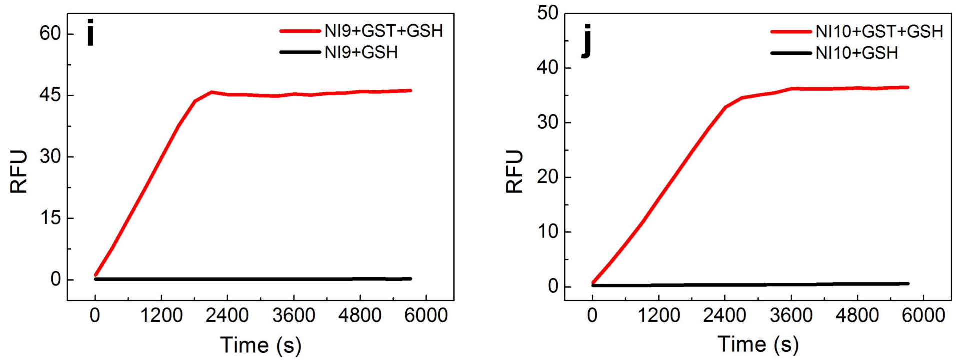

Figure S5. Respective time course of fluorescence intensity of probes **NI1−10** (20 μM) in HEPES buffer (20 mM, 0.5% DMSO, pH 7.4) with or without GSTs (12.5 μg/mL) from equine liver in the presence of GSH (1 mM). $\lambda_{ex/em}$ = 445/560 nm. GSTs were premixed with GSH for 10 min prior to the addition of probes.

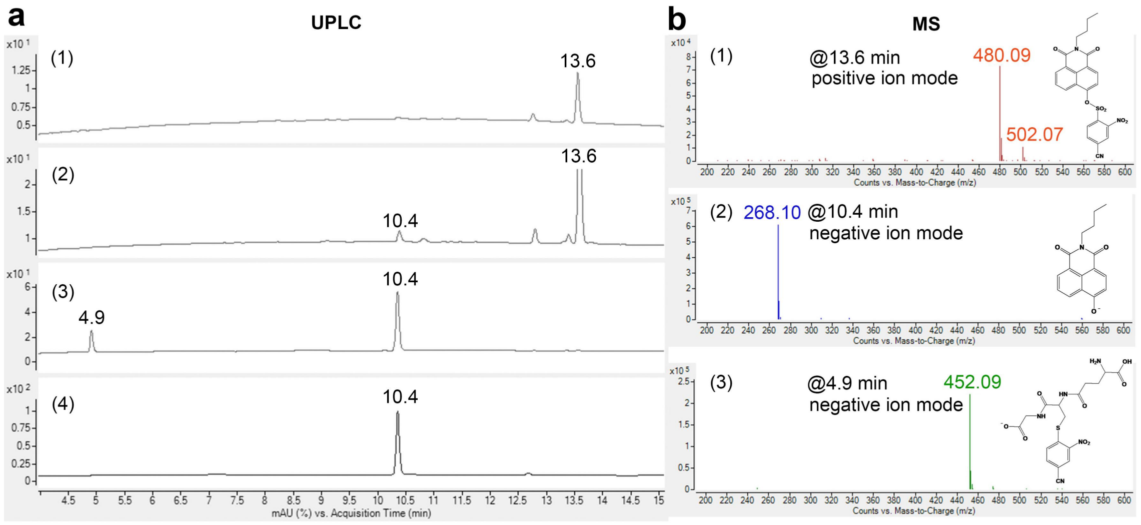

Figure S6. UPLC-MS analysis of the GST-catalysed reaction between **NI3** and GSH. **a**, UPLC traces of (**1**) **NI3**, (**2**) reaction mixture at 10 min after addition of GSTs (0.625 μg/mL) from equine liver, (**3**) reaction mixture at 2 h after addition of GSTs (12.5 μg/mL) from equine liver and (**4**) **NI**. Signal was detected based on absorbance at 370 nm. **b**, Mass spectra (MS) analysis of the peaks in UPLC. (**1**) The peak at 13.6 min corresponds to **NI3** ($[M+H]^+$, calcd. for $C_{23}H_{18}N_3O_7S^+$, 480.0860; found 480.09. $[M+Na]^+$, calcd. for $C_{23}H_{17}N_3NaO_7S^+$, 502.0679; found 502.07) . (**2**) The peak at 10.4 min corresponds to **NI** ($[M-H]^-$, calcd. for $C_{16}H_{14}NO_3^-$, 268.0979; found 268.10). (**3**) The peak at 4.9 min corresponds to the glutathione-conjugate **GS-3** ($[M-H]^-$, calcd. for $C_{17}H_{18}N_5O_8S^-$, 452.0882; found 452.09).

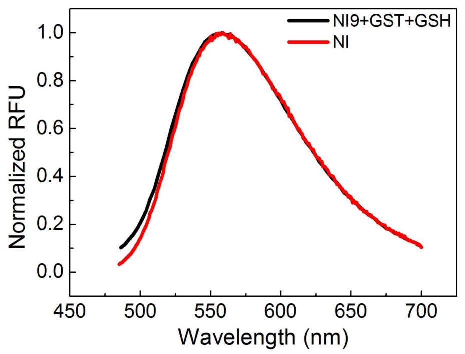

Figure S7. Fluorescence spectra of **NI** and the GST catalyzed reaction product with **NI9** and GSH being the reactants (prolong the time until the reaction nearly fully completed). Fluorescence intensity is normalized. λ_{ex} = 445 nm. The shapes of their fluorescence spectra are identical.

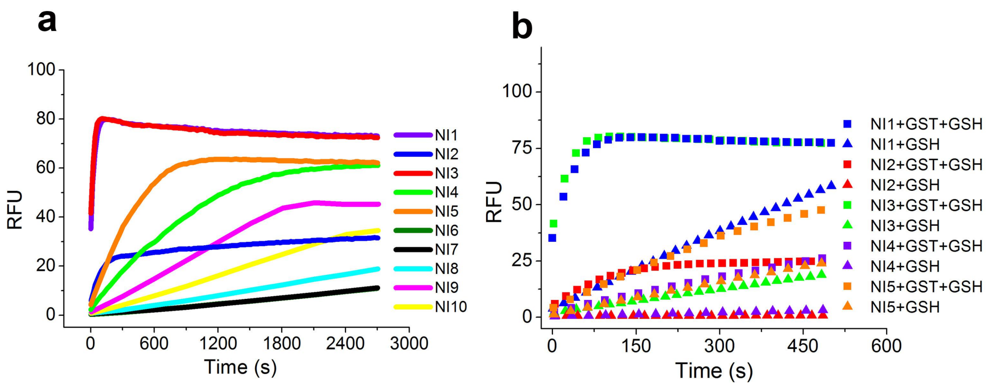

Figure S8. Comparison of probes in terms of (**a**) sensitivity and (**b**) relative S/N ratio. Data in Figure S5 were reprocessed to give this figure.

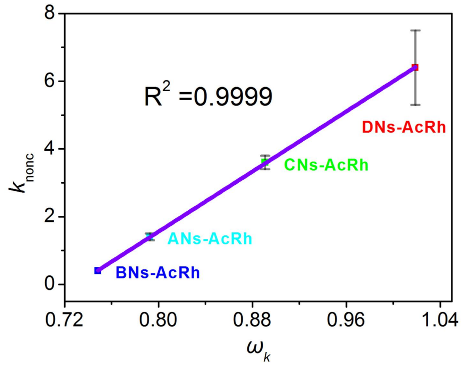

Figure S9. Structure-activity relationships between nonenzymatic kinetic parameters and the local electrophilicity ω_k of the α-carbon in terms of **AcRh**-series probes[17]. Note the kinetic data (k_{nonc}) were drawn from this reference[17].

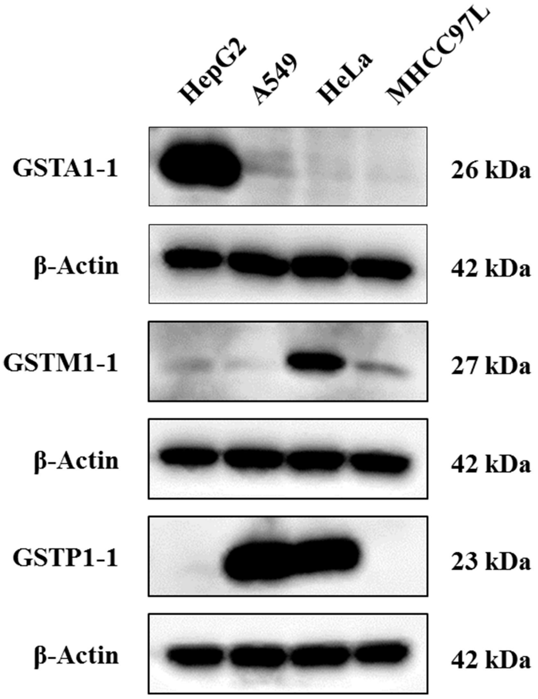

Figure S10. Western blotting analysis of the GST isoenzymes content in HepG2, A549, HeLa and MHCC97L cell lines**.** While other three cell lines are rich in one or two of the GST isoenzymes, MHCC97L cell line contains hardly any GST.

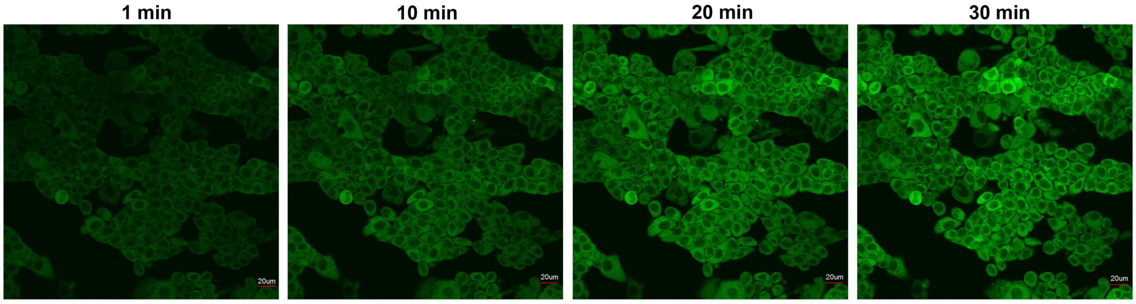

Figure S11. Confocal fluorescence images of HepG2 cells at different time points after incubation with 20 µM **NI3** in HEPES buffer (20 mM, 0.5% DMSO, 5% glucose, pH 7.4). Images were acquired upon excitation at 458 nm with a 40× objective. λ_{em} = 500−600 nm. Scale bar = 20 µm.

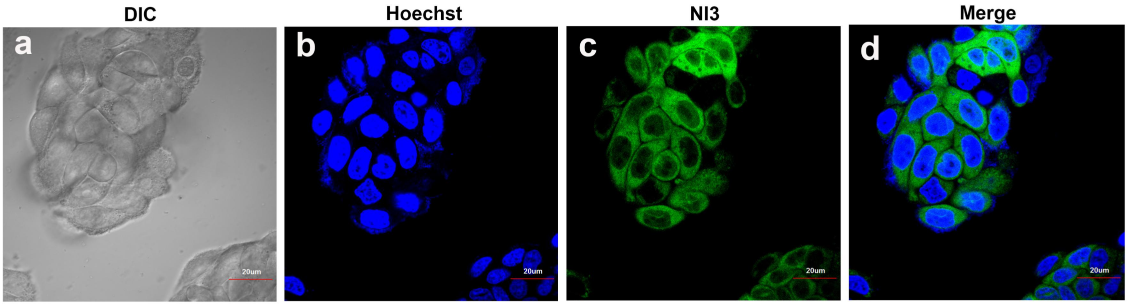

Figure S12. Fluorescence images of HepG2 cells co-incubated with Hoechst 33342 (a commercial nucleus-staining dye) and **NI3**. **a**, DIC image. **b**, Hoechst. **c**, **NI3**. **d**, Merged image of **b** and **c**. The excitation wavelengths for **b** and **c** were 405 and 488 nm, respectively, and the corresponding emissions were collected at 440–490 nm (Hoechst 33342) and 500–600 nm (**NI3**). Scale bar = 20 μm. Representative images from repeated experiments are shown.

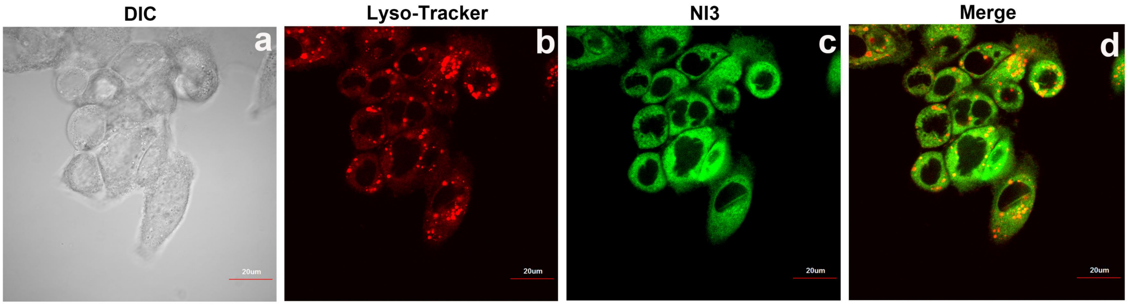

Figure S13. Fluorescence images of HepG2 cells co-incubated with Lyso-Tracker Red and **NI3**. **a**, DIC image. **b**, Lyso-Tracker Red. **c**, **NI3**. **d**, Merged image of **b** and **c**. The excitation wavelengths for **b** and **c** were 543 and 405 nm, respectively, and the corresponding emissions were collected at 580–650 nm (Lyso-Tracker Red) and 500–560 nm (**NI3**). Scale bar = 20 μm. Representative images from repeated experiments are shown.

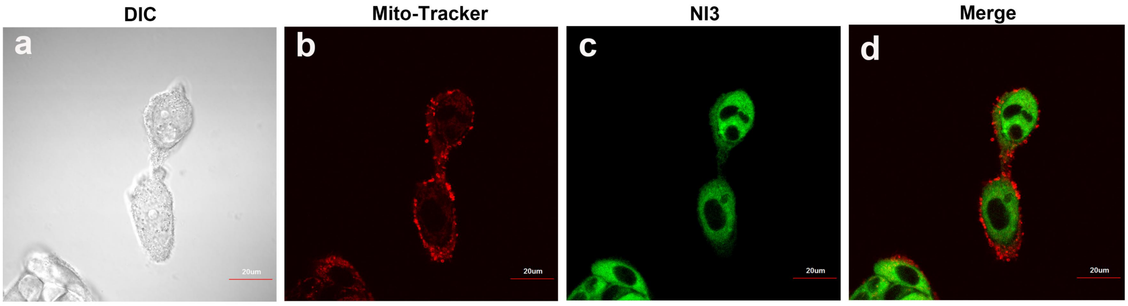

Figure S14. Fluorescence images of HepG2 cells co-incubated with Mito-Tracker Red and **NI3**. **a**, DIC image. **b**, Mito-Tracker Red. **c**, **NI3**. **d**, Merged image of **b** and **c**. The excitation wavelengths for **b** and **c** were 543 and 405 nm, respectively, and the corresponding emissions were collected at 580–650 nm (Mito-Tracker Red) and 500–560 nm (**NI3**). Scale bar = 20 μm. Representative images from repeated experiments are shown.

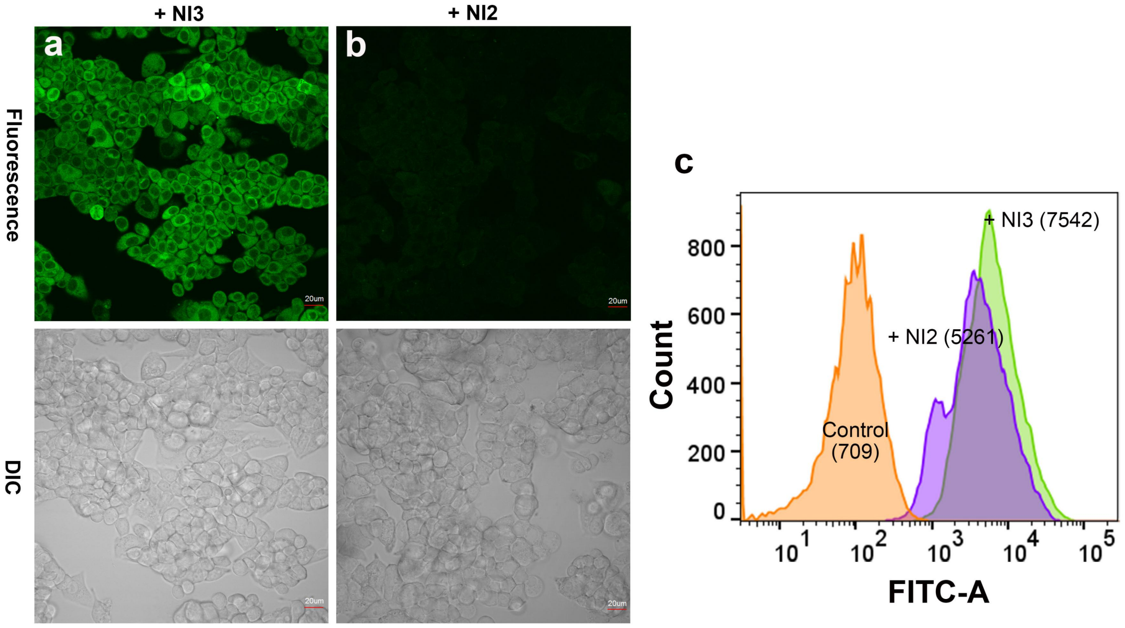

Figure S15. Comparison of the performances of **NI3** and **NI2** in (**a**,**b**) celluar fluorescence imaging and (**c**) flow cytometry with HepG2 cells incubated with 20 μM probes respectively for 30 min. **a** and **b**, 40× objective. λ_{ex} = 458 nm. λ_{em} = 500−600 nm. Scale bar = 20 μm. Representative images from repeated experiments are shown. **c**, The numbers within brackets are corresponding respective mean fluorescence intensity. λ_{ex} = 488 nm. λ_{em} = 500−600 nm.

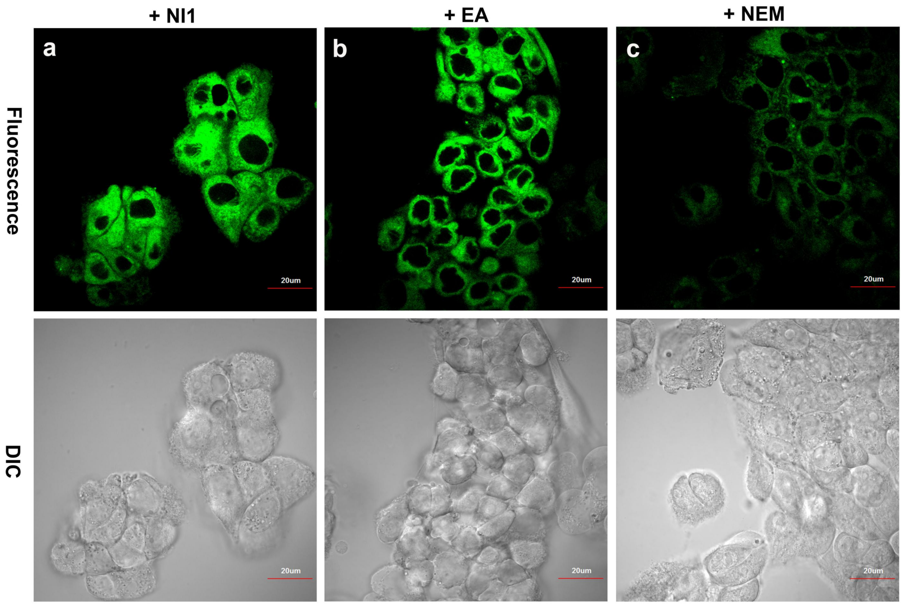

Figure S16. Single-photon confocal fluorescence imaging of HepG2 cells (a) incubated with 20 μM **NI1**, (b) pretreated with 100 μM EA and then incubated with 20 μM **NI1** and (c) pretreated with 50 μM NEM and then incubated with 20 μM **NI1** with a 100× objective.

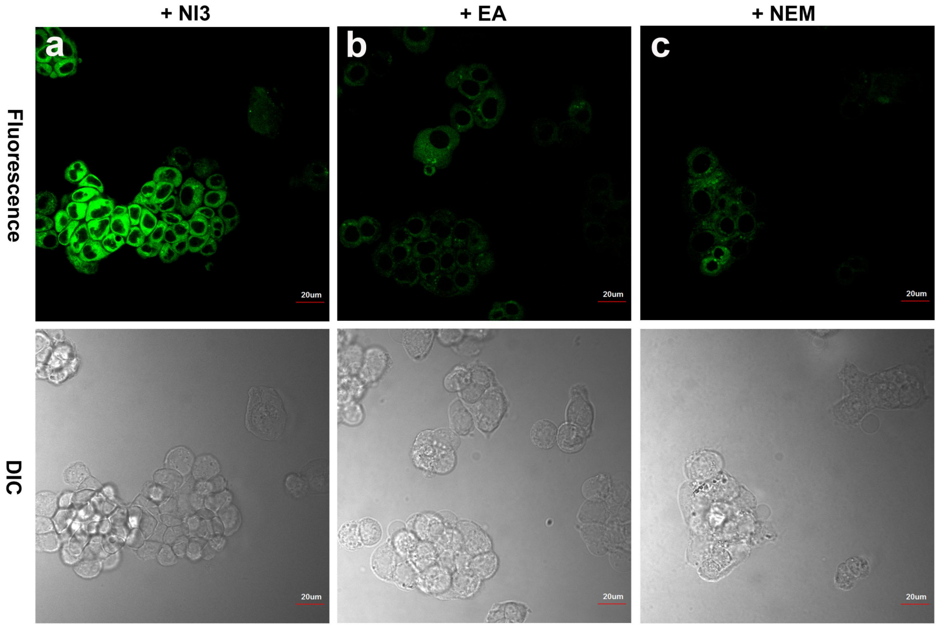

Figure S17. Two-photon fluorescence images of HepG2 cells (**a**) incubated with 20 μM **NI3** for 30 min, (**b**) pretreated with 100 μM EA for 30 min and then incubated with 20 μM **NI3** for 30 min and (**c**) pretreated with 50 μM NEM for 45 min and then incubated with 20 μM **NI3** for 30 min with a 60× objective. λ_{ex} = 810 nm. λ_{em} = 520−560 nm. Scale bar = 20 μm. Representative images from repeated experiments are shown.

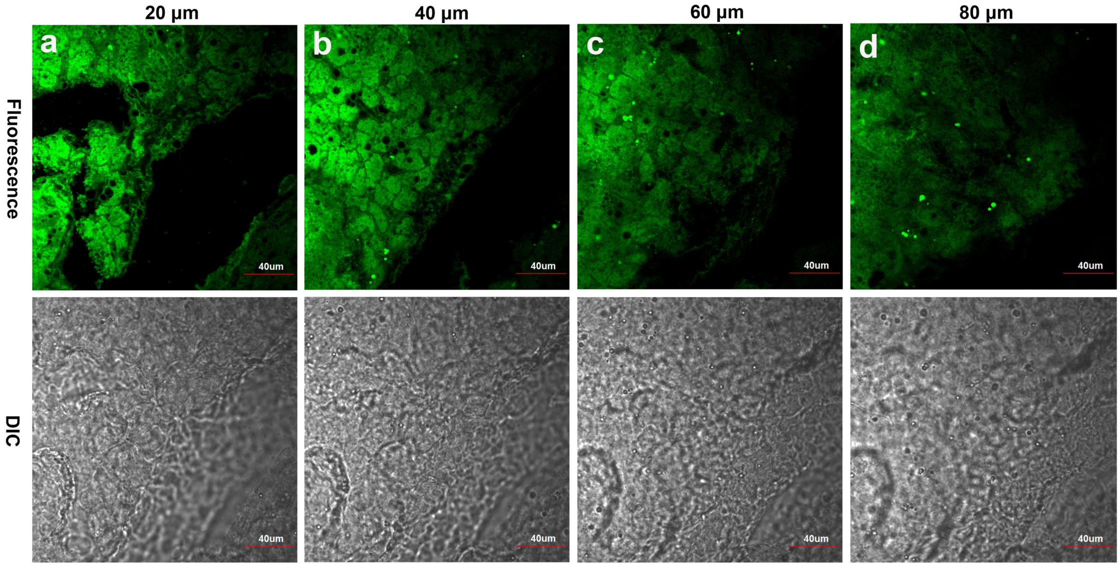

Figure S18. Two-photon fluorescence images of mice liver tissue sections incubated with 40 μM **NI3** for 1 h at different depths with a 60× objective. λ_{ex} = 810 nm. λ_{em} = 520−560 nm. Scale bar = 40 μm.

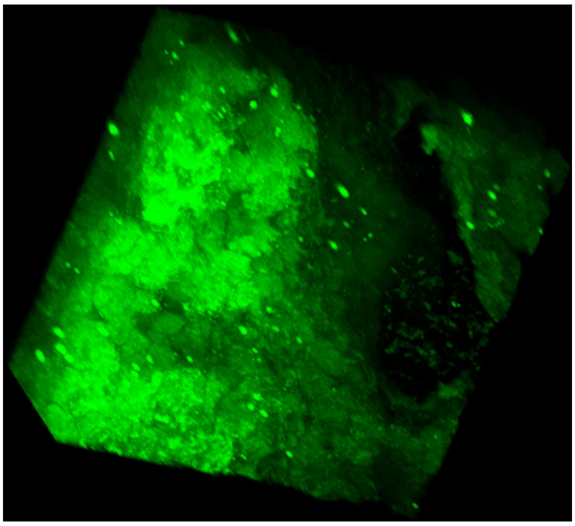

Figure S19. 3D reconstruction of the two-photon fluorescence images of mice liver tissue sections.

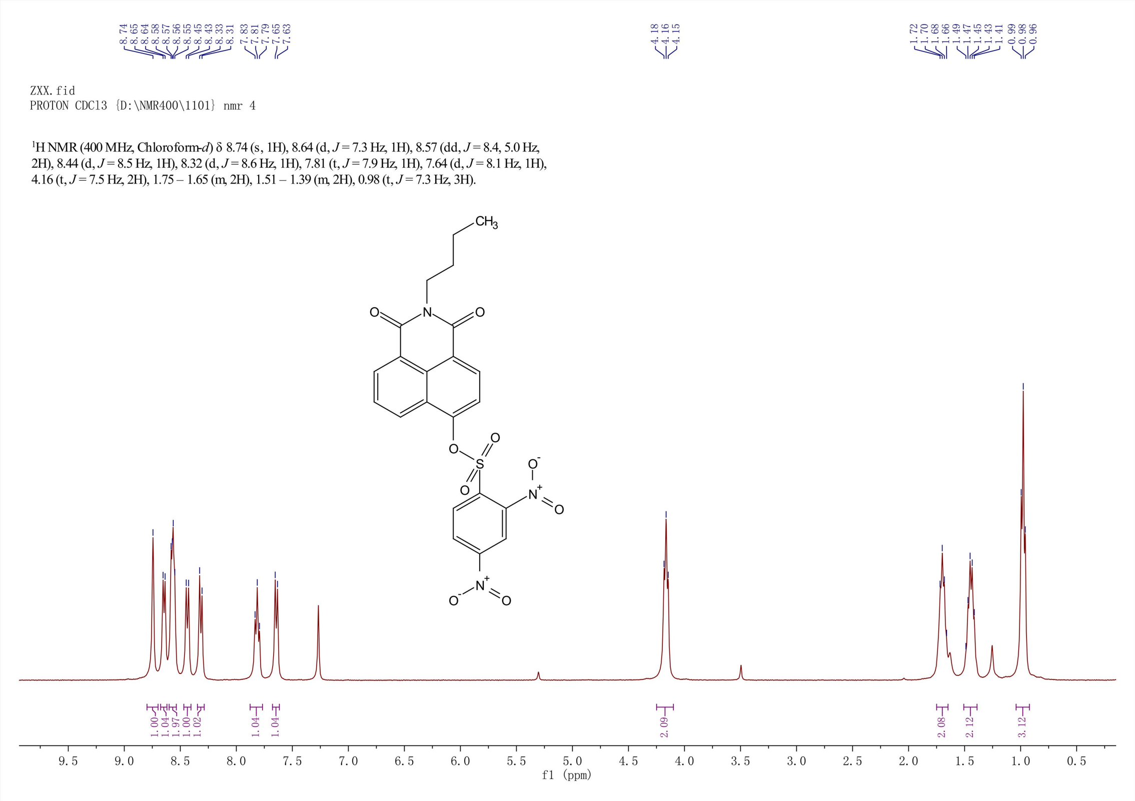

Figure S20. ^{1}H NMR spectrum (400 MHz) of **NI1** in Chloroform-*d*.

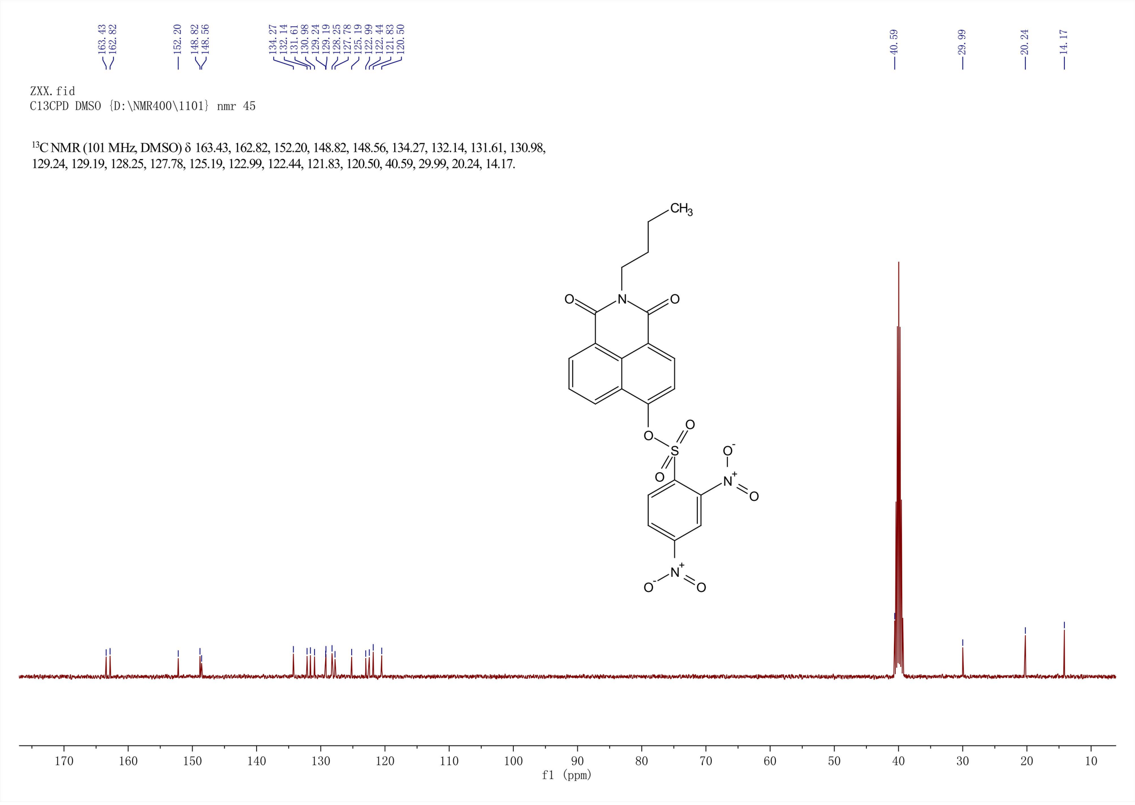

Figure S21. ^{13}C NMR spectrum (101 MHz) of **NI1** in DMSO.

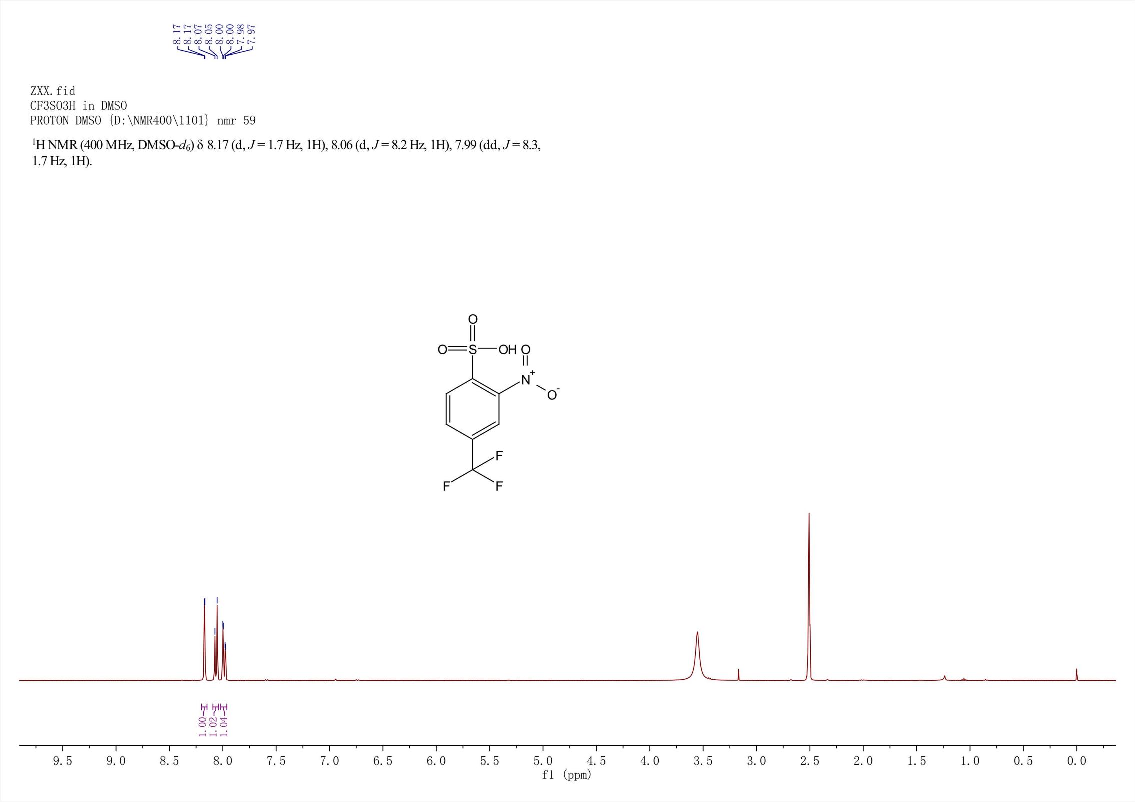

Figure S22. ^{1}H NMR spectrum (400 MHz) of **NI2-SO$_3$H** in DMSO.

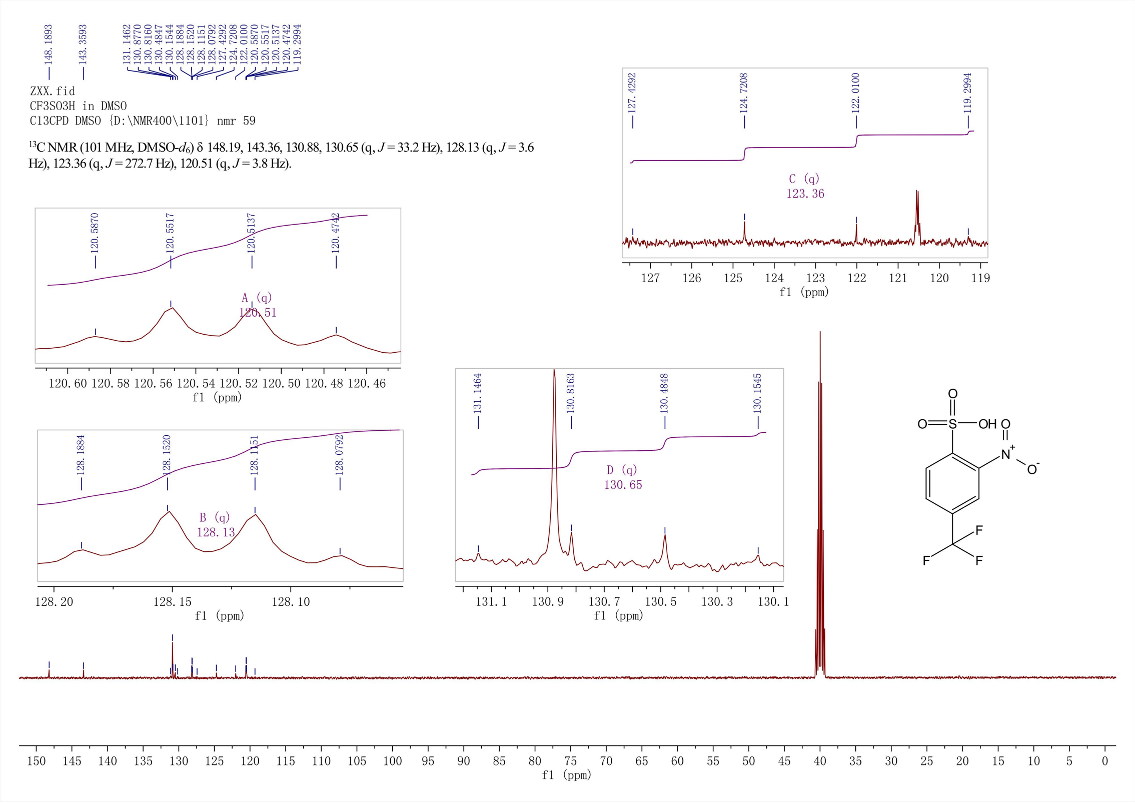

Figure S23. ^{13}C NMR spectrum (101 MHz) of **NI2-SO$_3$H** in DMSO.

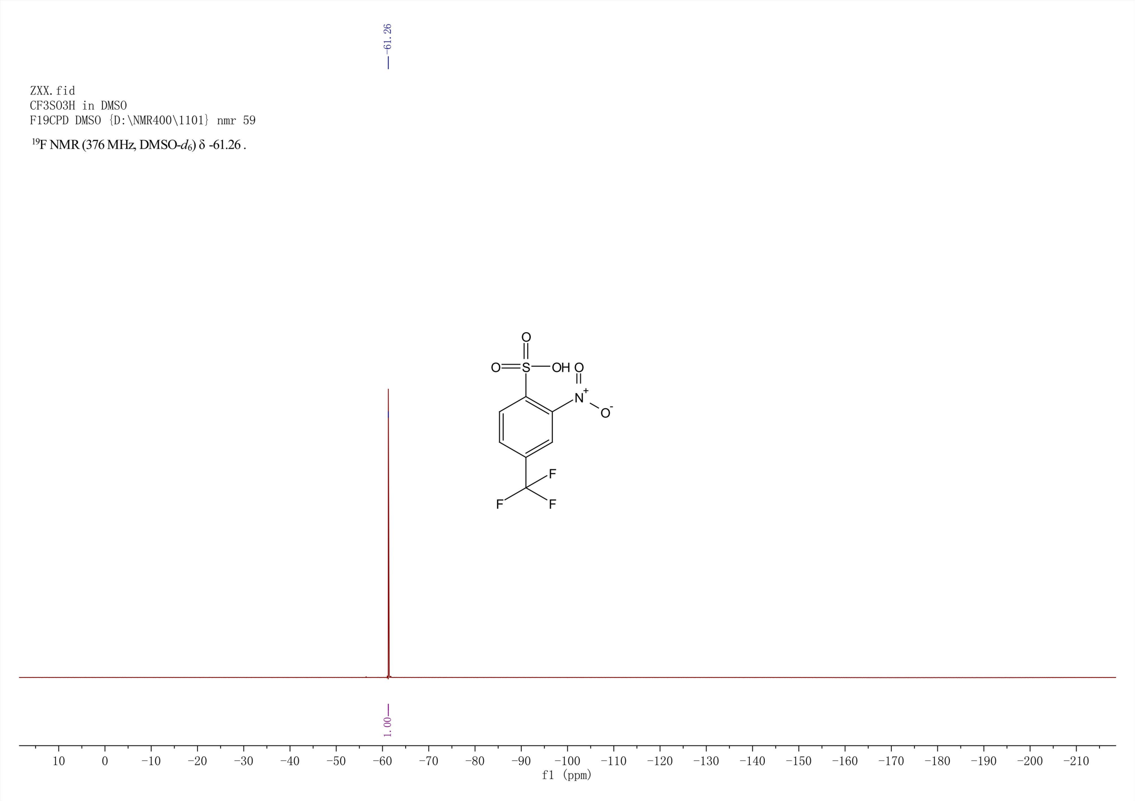

Figure S24. ^{19}F NMR spectrum (376 MHz) of **NI2-SO$_3$H** in DMSO.

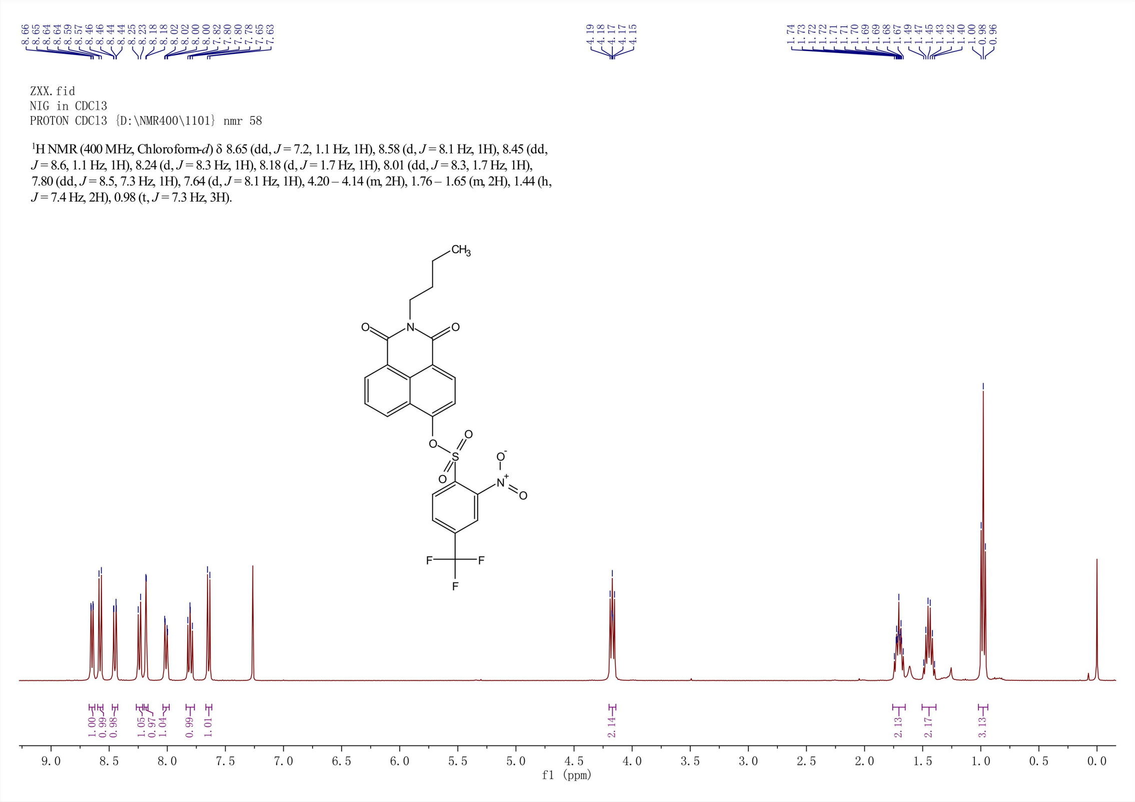

Figure S25. ^{1}H NMR spectrum (400 MHz) of **NI2** in Chloroform-*d*.

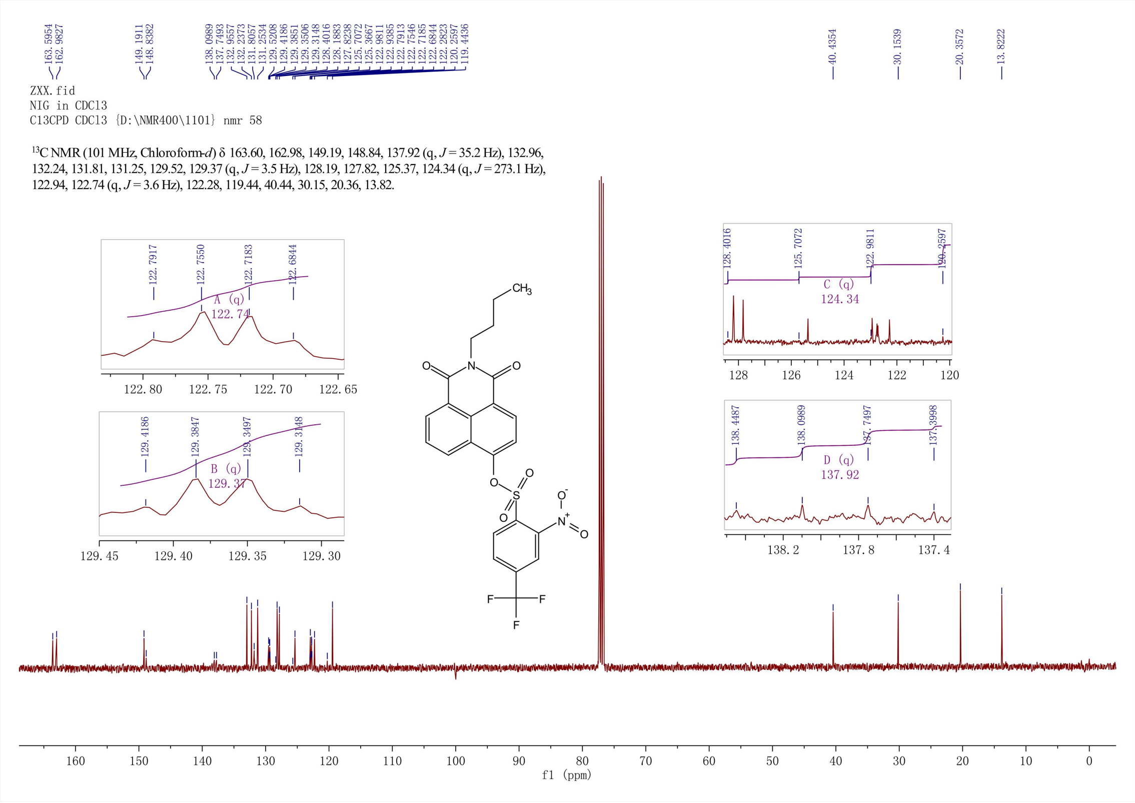

Figure S26. ^{13}C NMR spectrum (101 MHz) of **NI2** in Chloroform-*d*.

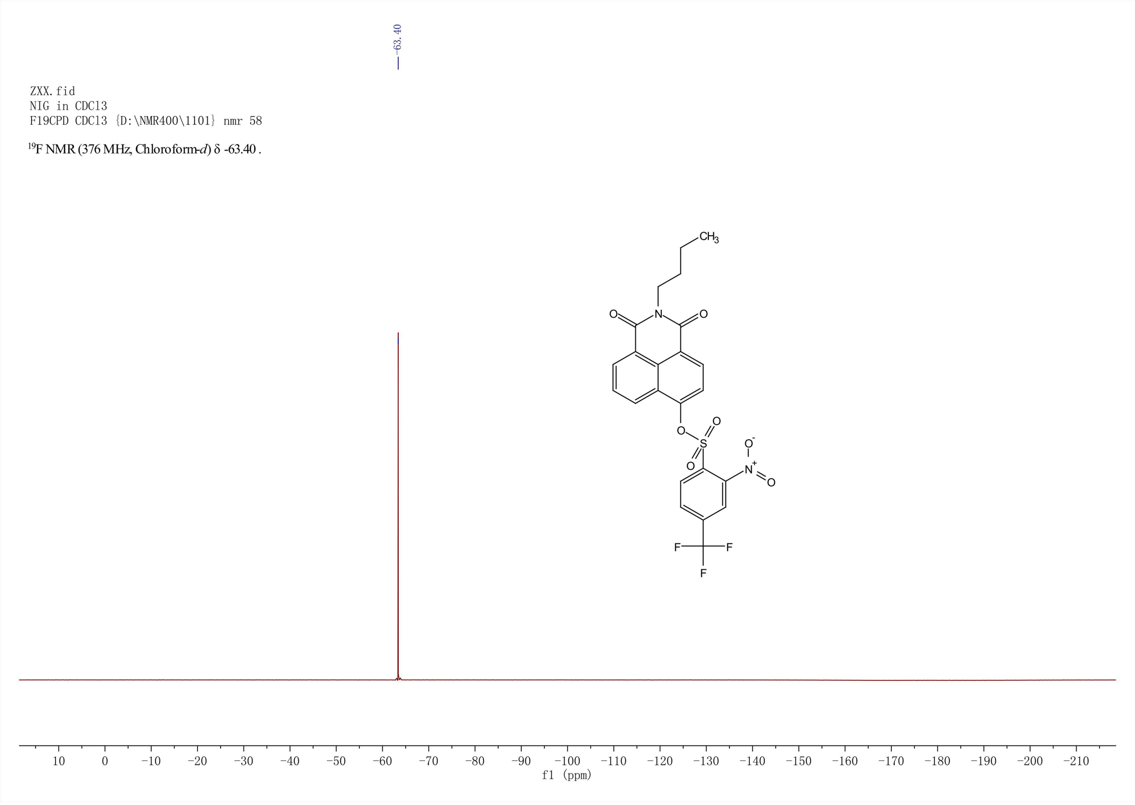

Figure S27. ^{19}F NMR spectrum (376 MHz) of **NI2** in Chloroform-*d*.

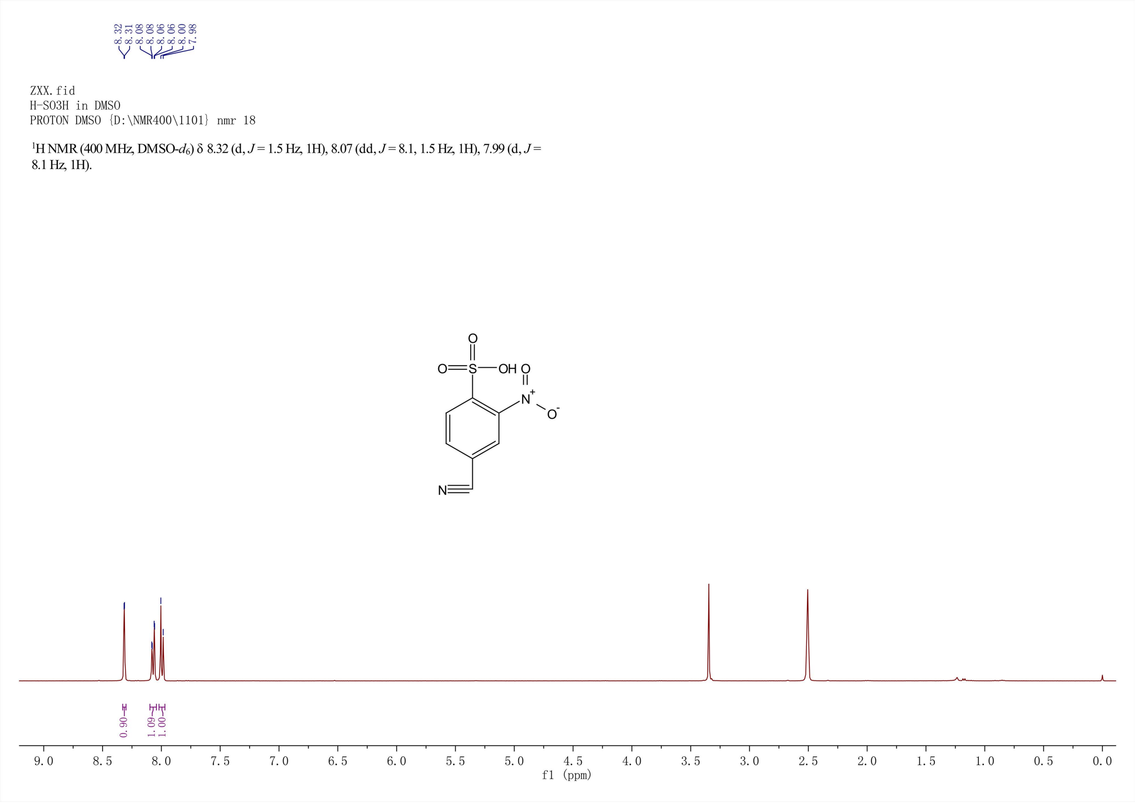

Figure S28. ^{1}H NMR spectrum (400 MHz) of **NI3-SO$_3$H** in DMSO.

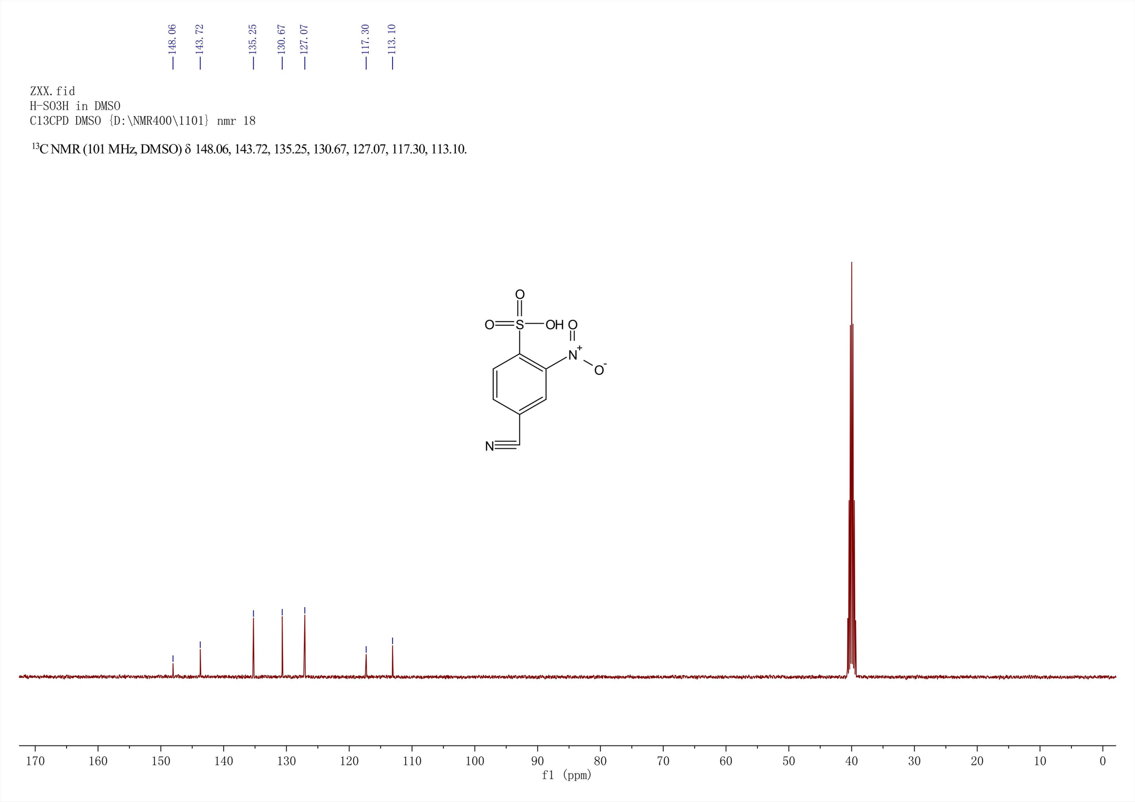

Figure S29. ^{13}C NMR spectrum (101 MHz) of **NI3-SO$_3$H** in DMSO.

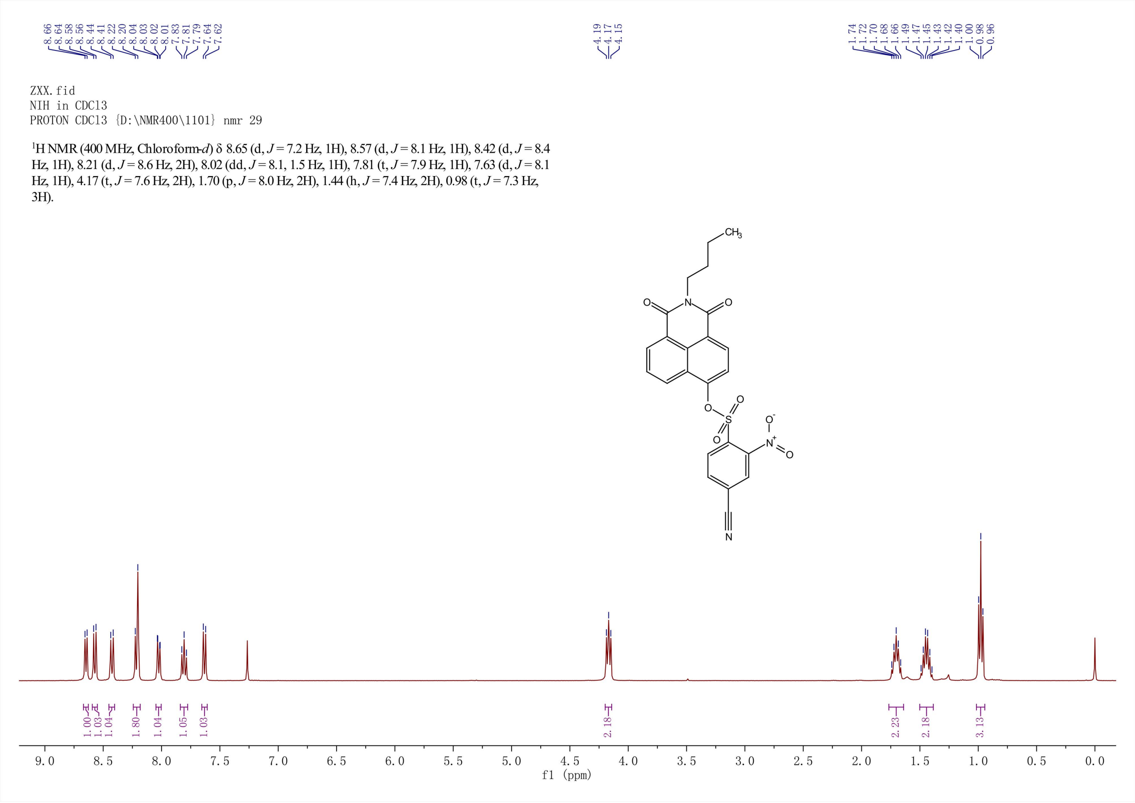

Figure S30. ^{1}H NMR spectrum (400 MHz) of **NI3** in Chloroform-*d*.

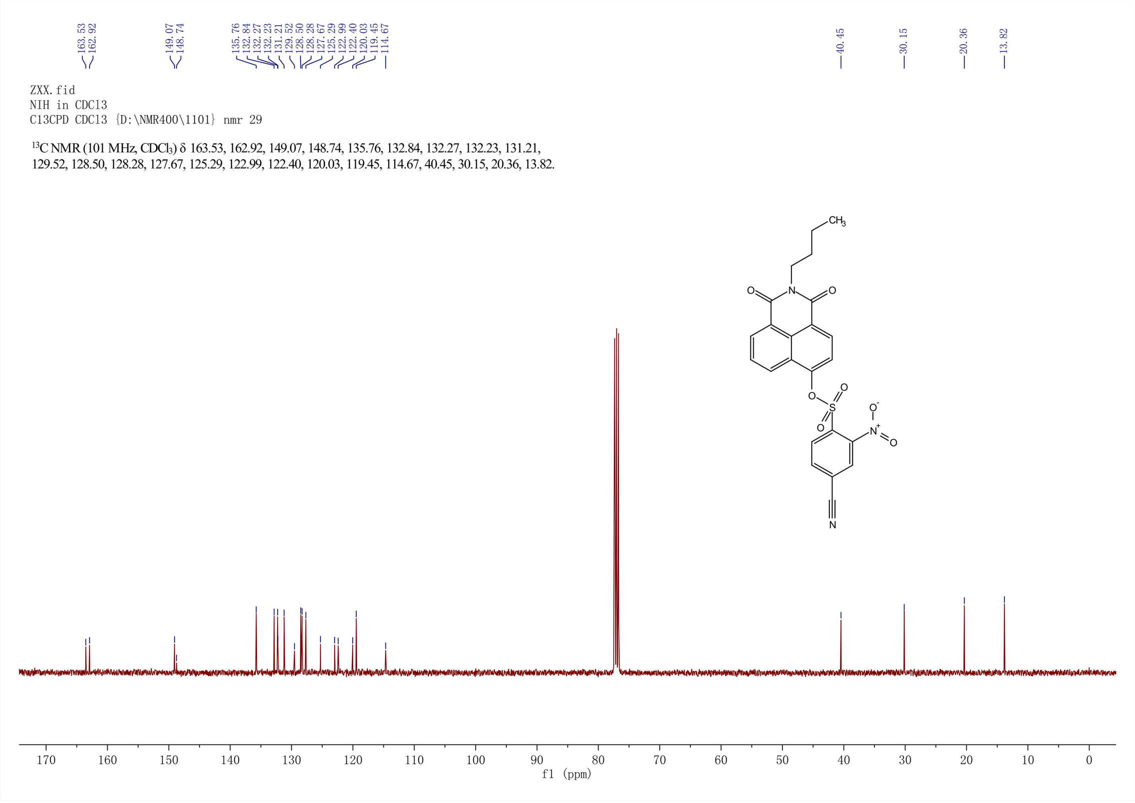

Figure S31. ^{13}C NMR spectrum (101 MHz) of **NI3** in Chloroform-*d*.

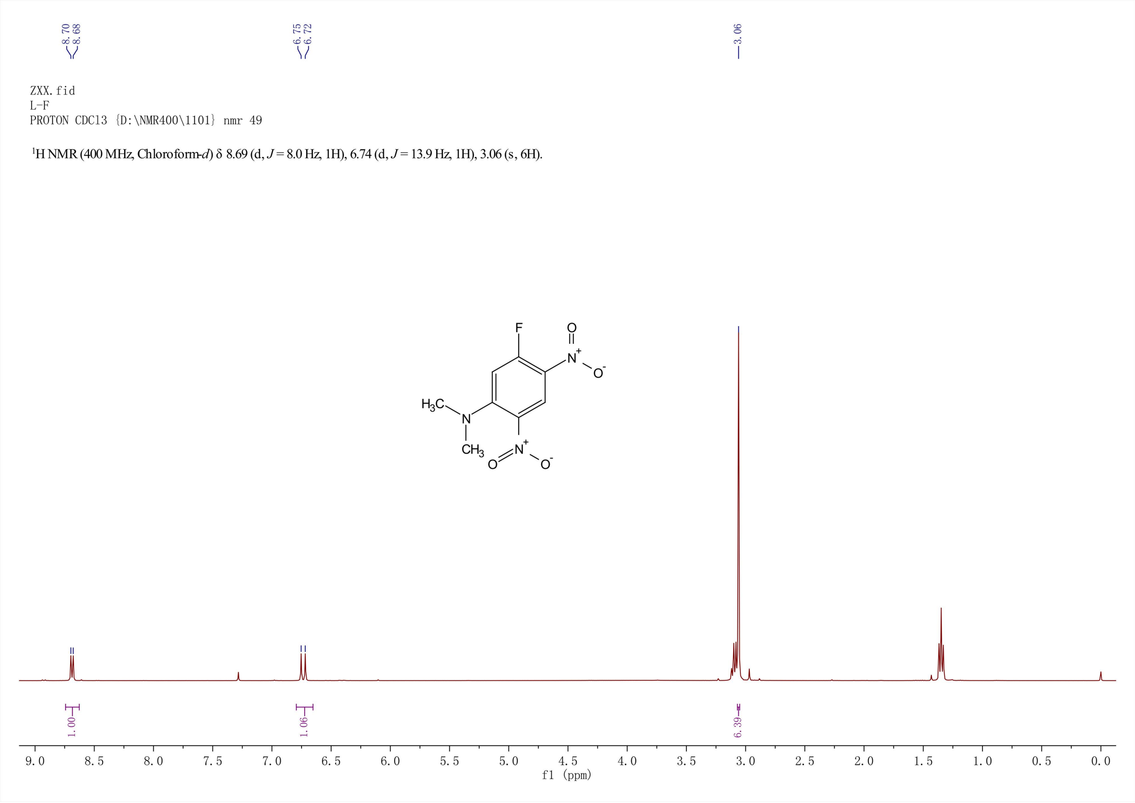

Figure S32. ^{1}H NMR spectrum (400 MHz) of **NI4-F** in Chloroform-*d*.

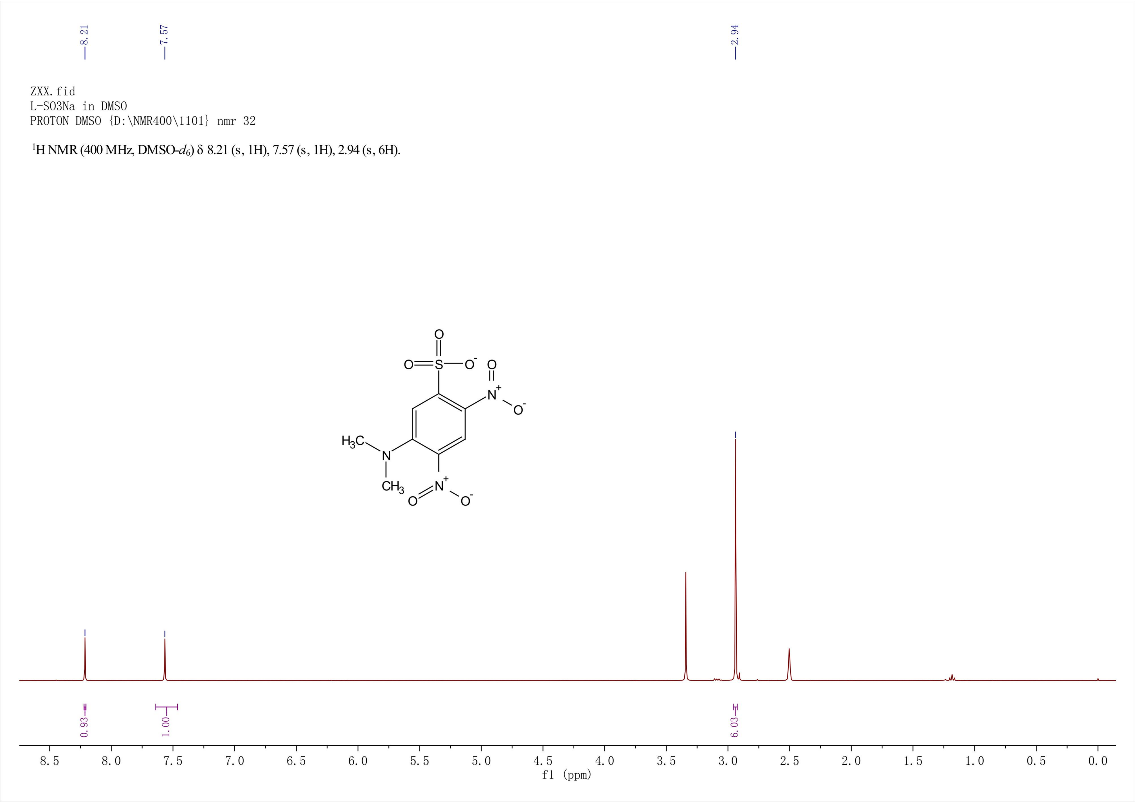

Figure S33. ^{1}H NMR spectrum (400 MHz) of **NI4-SO_3Na** in DMSO.

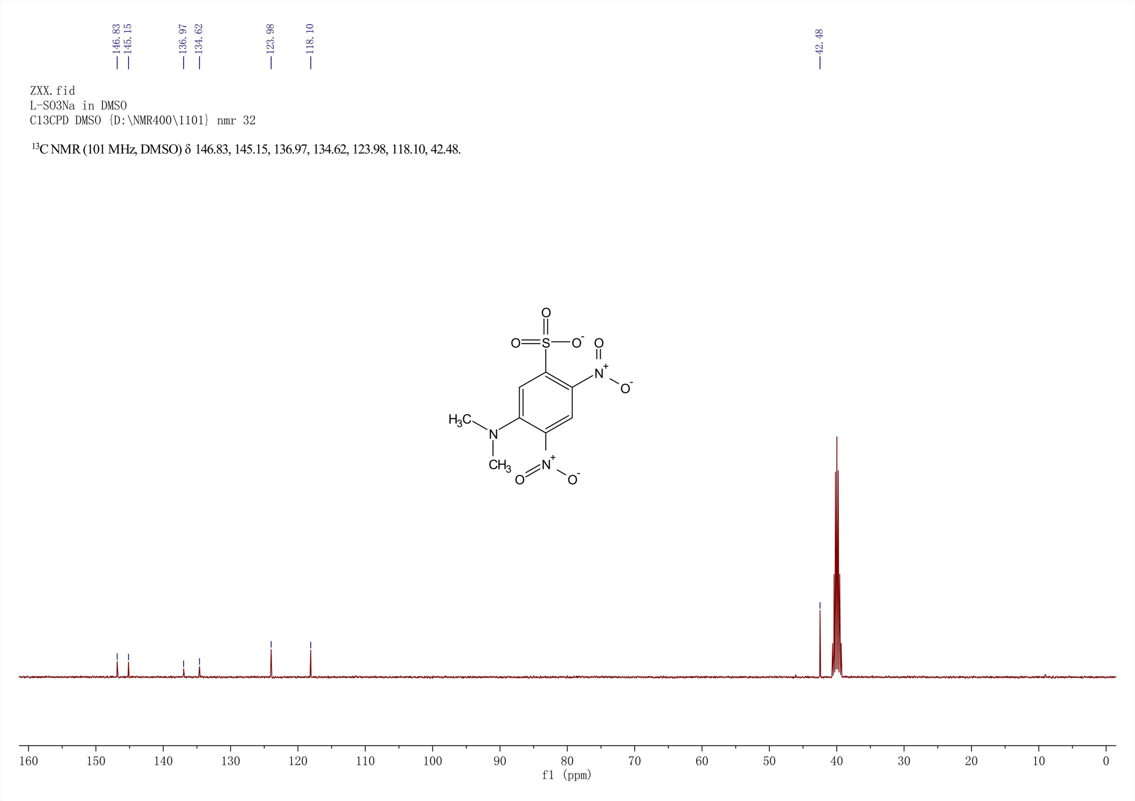

Figure S34. ^{13}C NMR spectrum (101 MHz) of **NI4-SO$_3$Na** in DMSO.

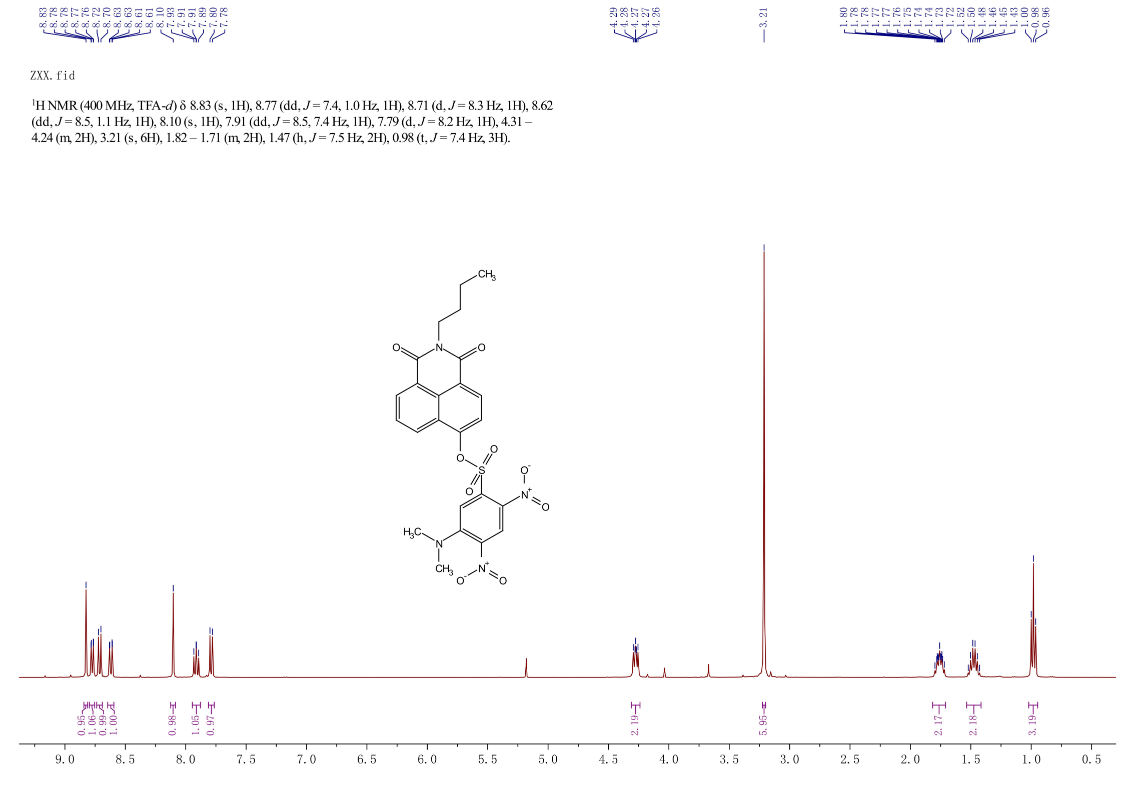

Figure S35. ^{1}H NMR spectrum (400 MHz) of **NI4** in TFA-*d*.

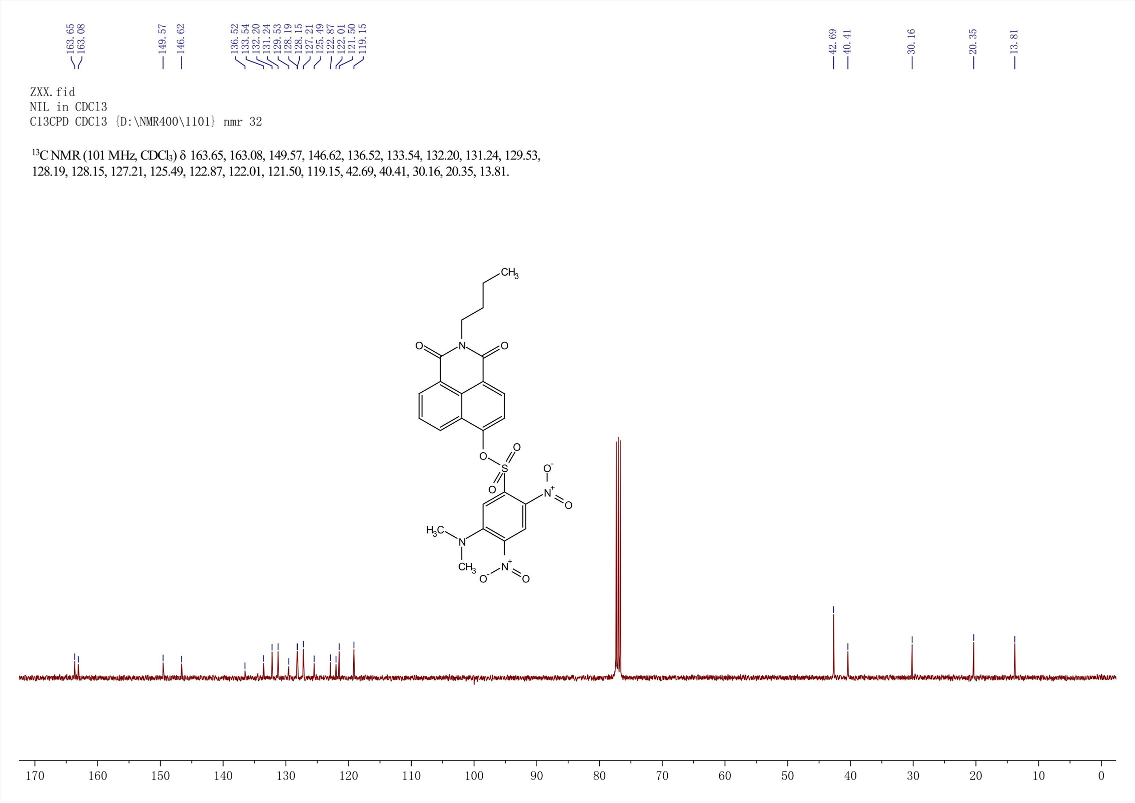

Figure S36. ^{13}C NMR spectrum (101 MHz) of **NI4** in Chloroform-*d*.

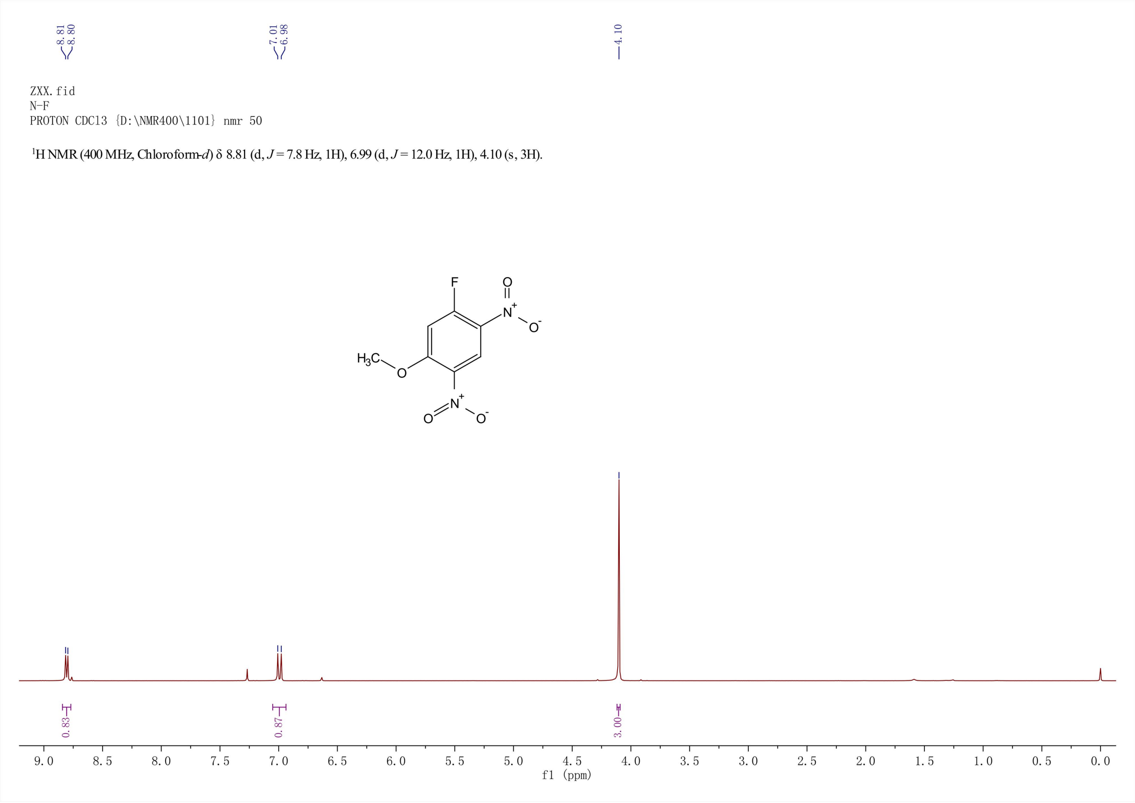

Figure S37. ^{1}H NMR spectrum (400 MHz) of **NI5-F** in Chloroform-*d*.

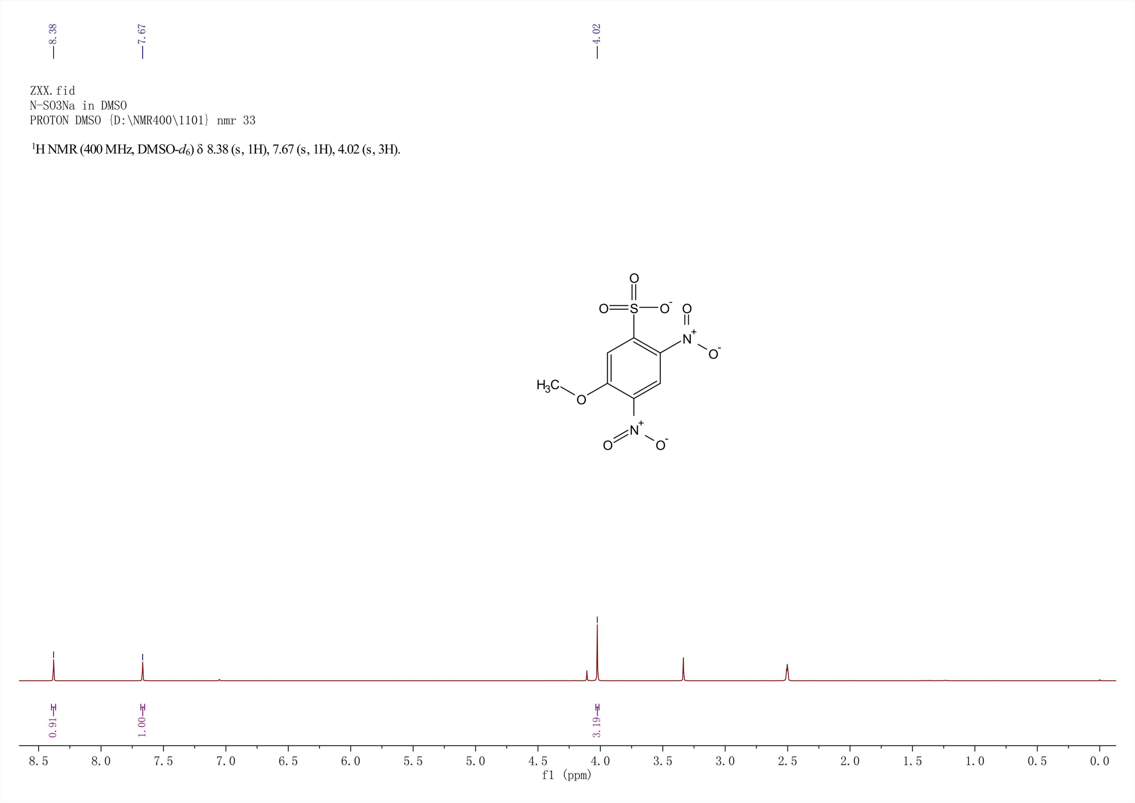

Figure S38. ^{1}H NMR spectrum (400 MHz) of **NI5-SO₃Na** in DMSO.

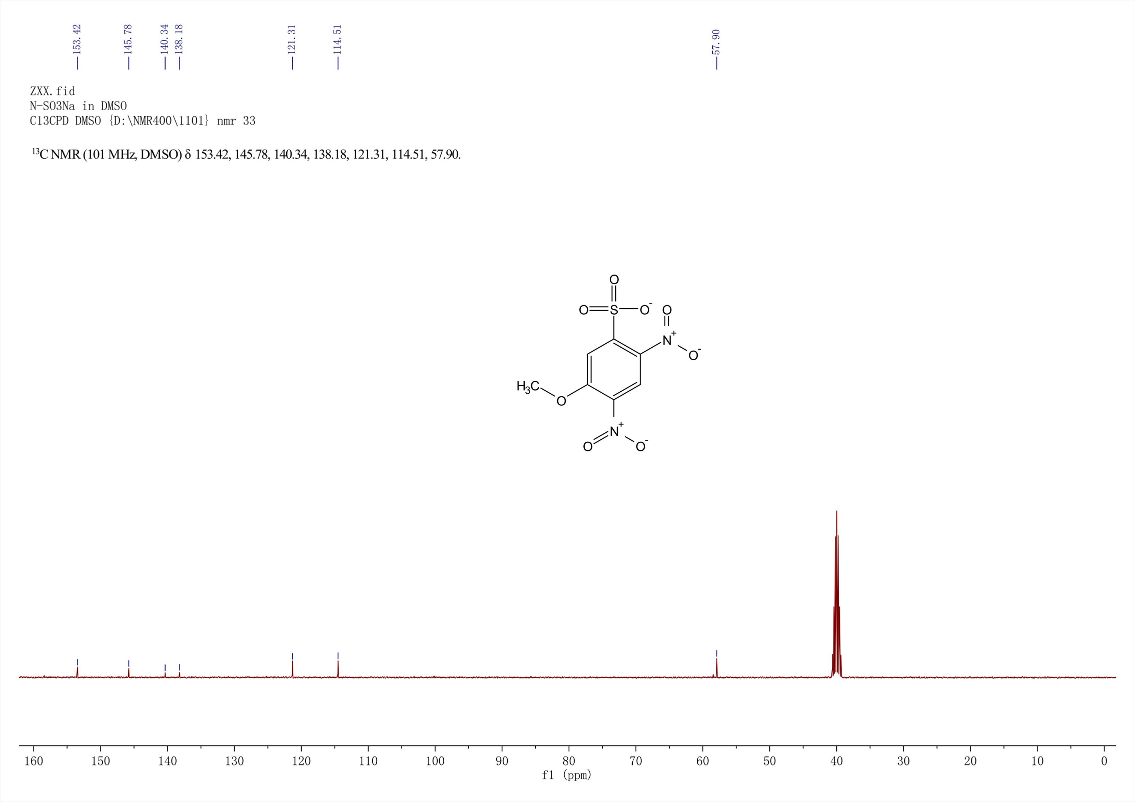

Figure S39. ^{13}C NMR spectrum (101 MHz) of **NI5-SO$_3$Na** in DMSO.

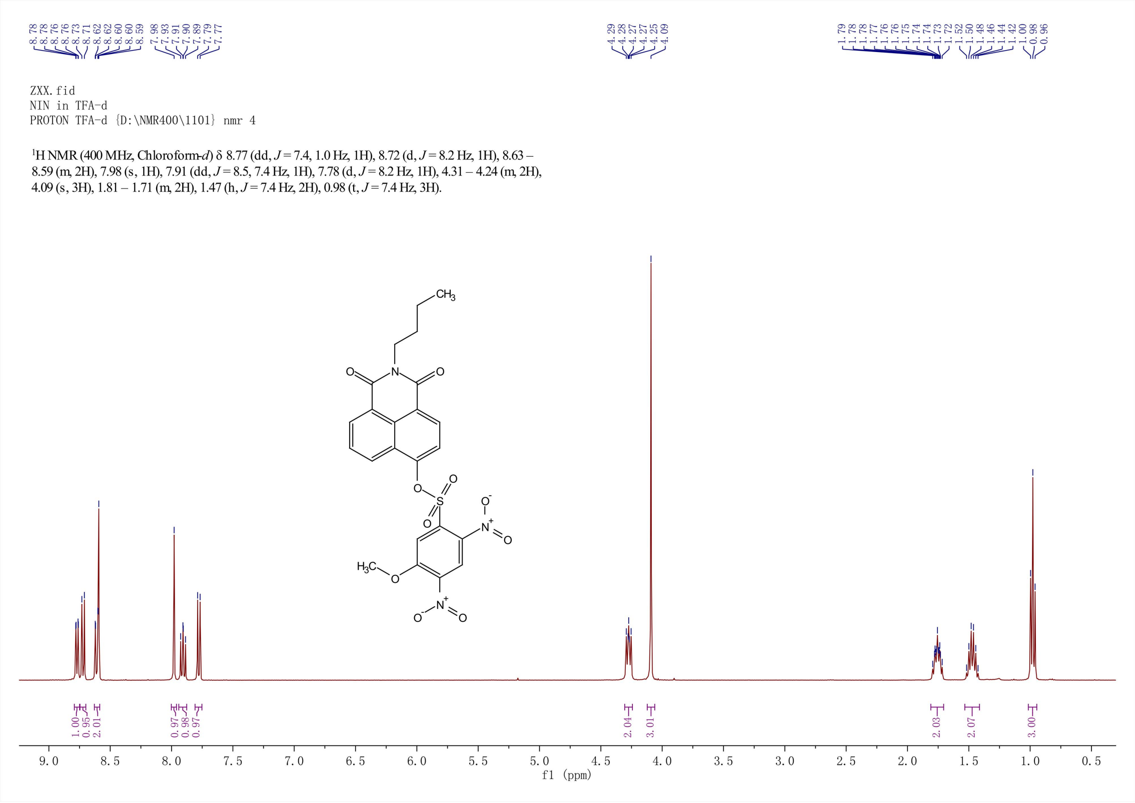

Figure S40. ^{1}H NMR spectrum (400 MHz) of **NI5** in TFA-*d*.

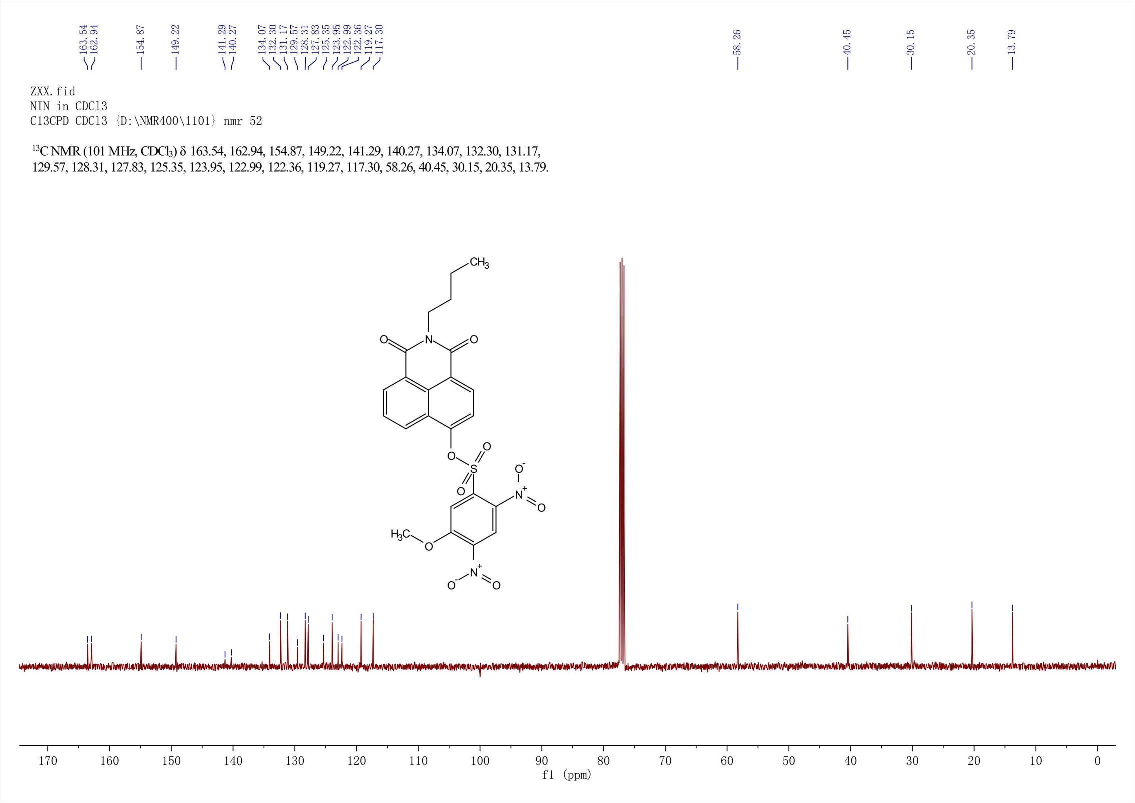

Figure S41. ^{13}C NMR spectrum (101 MHz) of **NI5** in Chloroform-*d*.

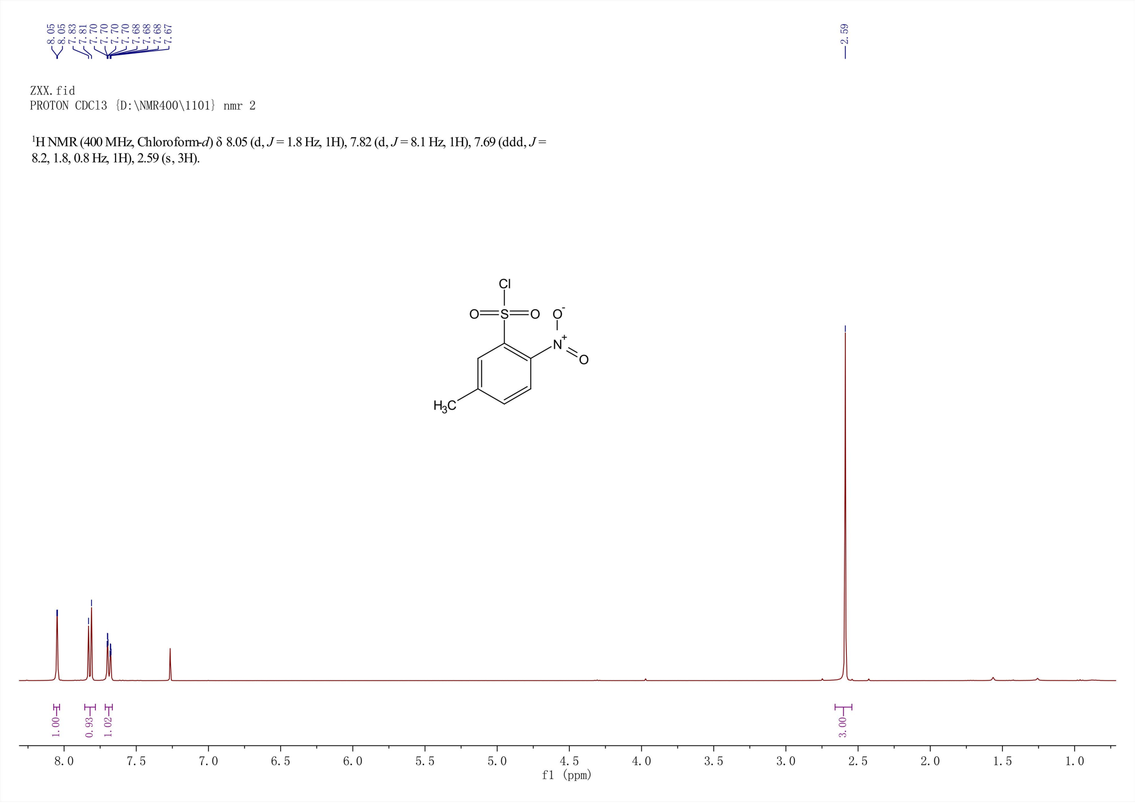

Figure S42. ^{1}H NMR spectrum (400 MHz) of **NI6-SO$_2$Cl** in $CDCl_3$.

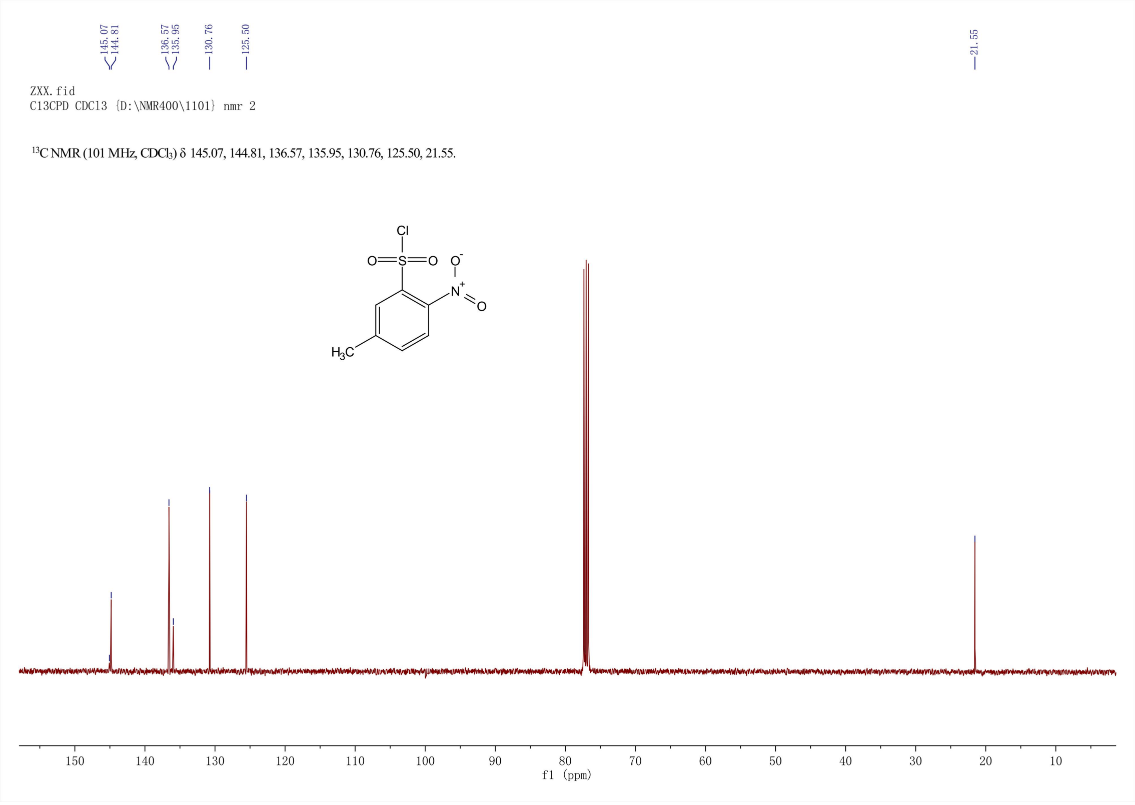

Figure S43. ^{13}C NMR spectrum (101 MHz) of **NI6-SO$_2$Cl** in $CDCl_3$.

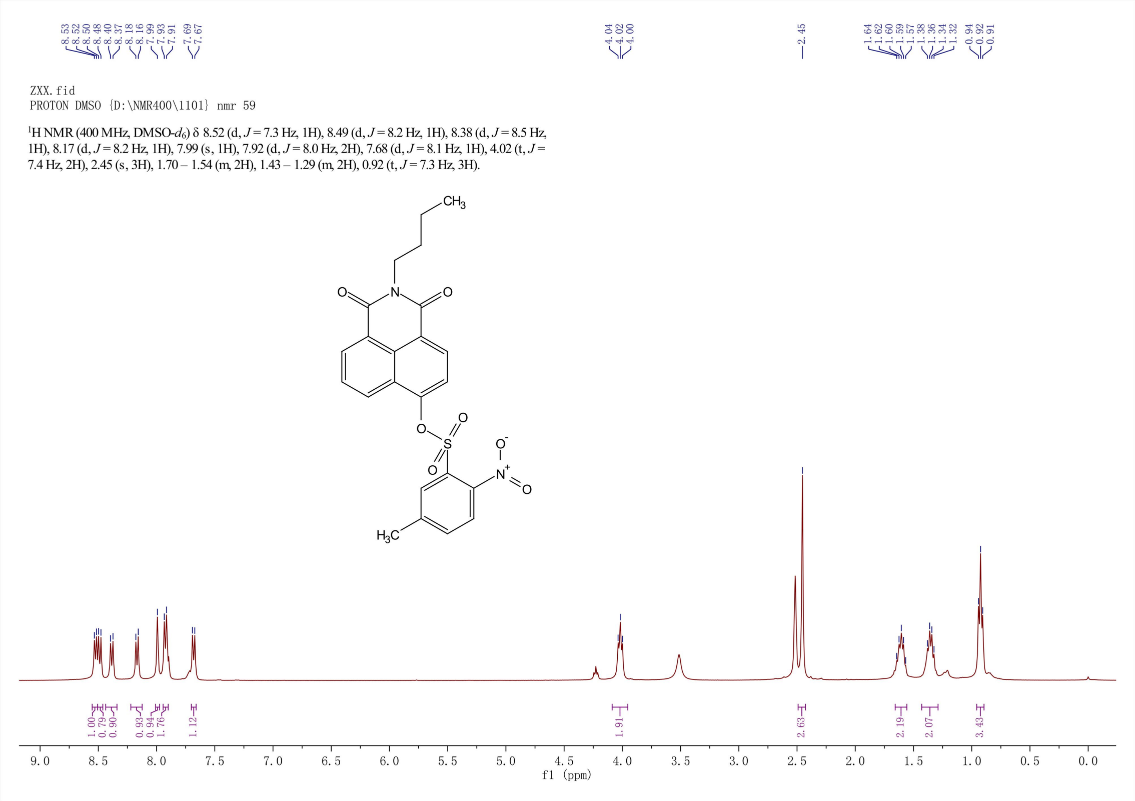

Figure S44. ^{1}H NMR spectrum (400 MHz) of **NI6** in DMSO.

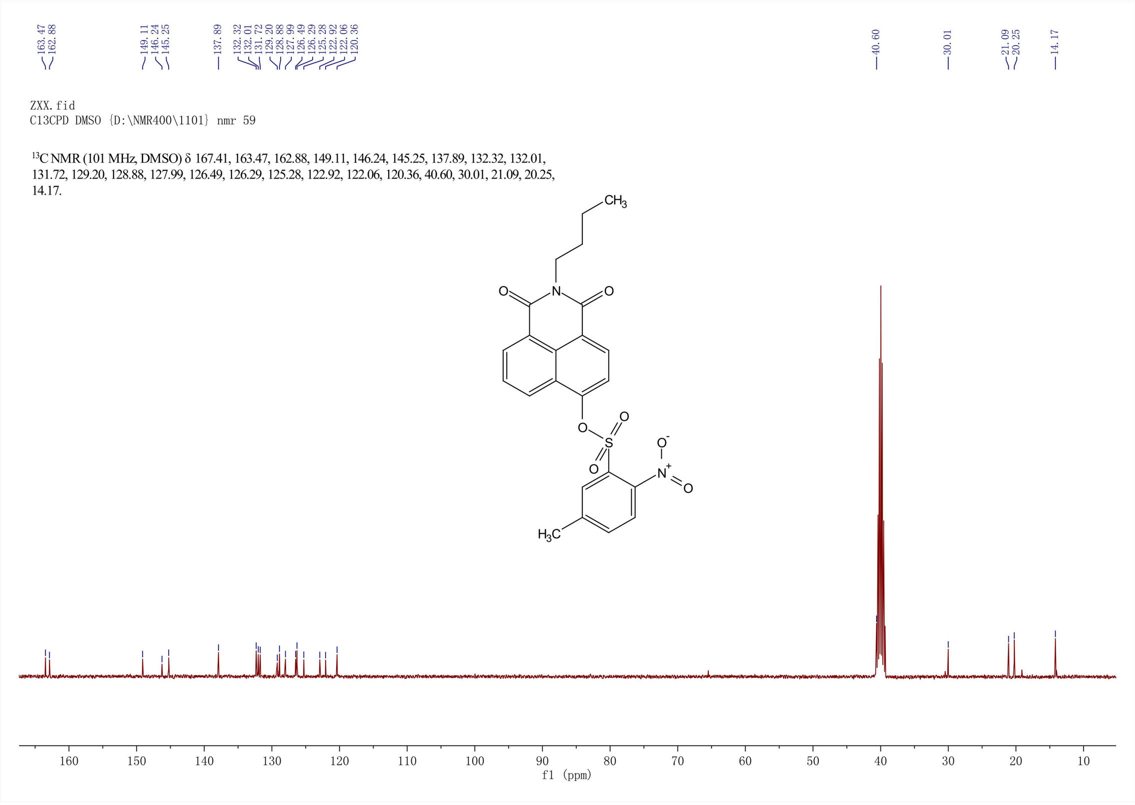

Figure S45. ^{13}C NMR spectrum (101 MHz) of **NI6** in DMSO.

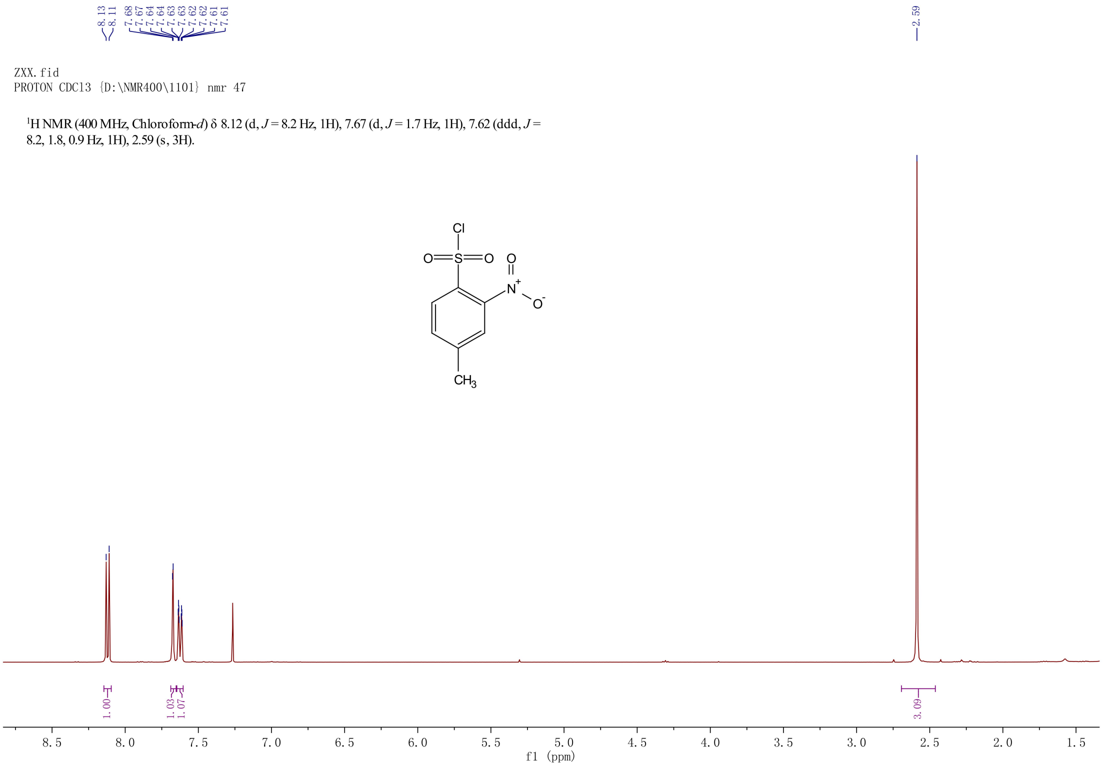

Figure S46. ^{1}H NMR spectrum (400 MHz) of **NI7-SO_2Cl** in $CDCl_3$.

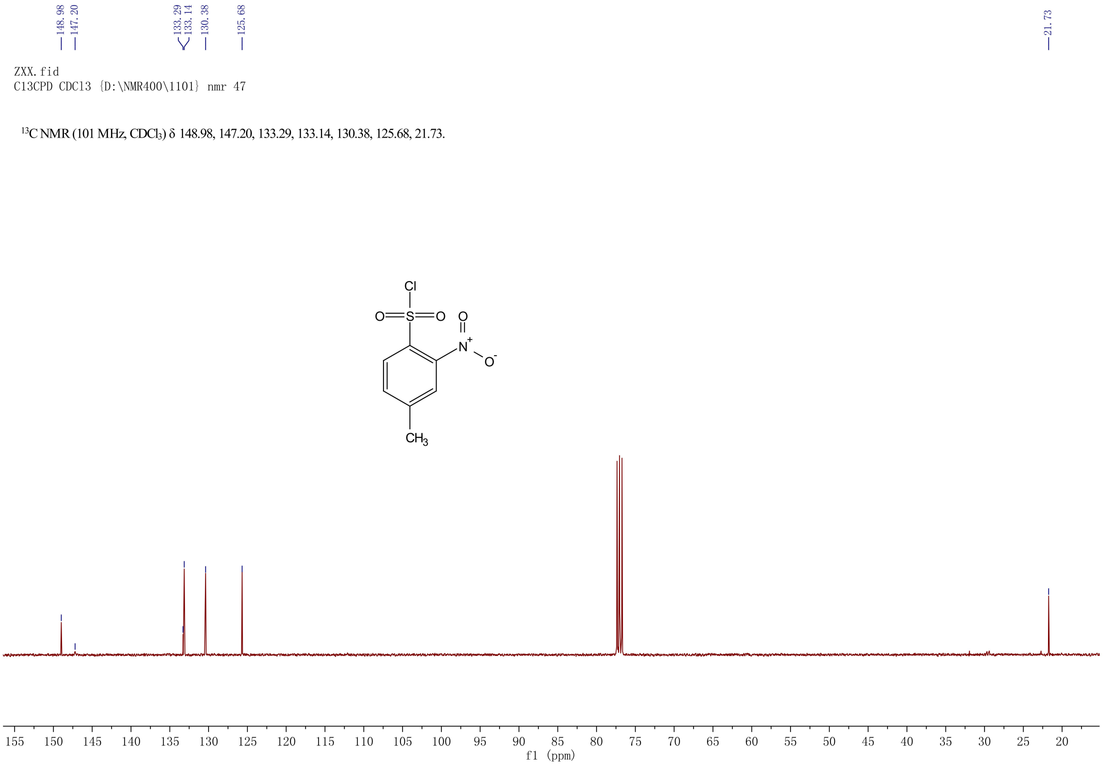

Figure S47. ^{13}C NMR spectrum (101 MHz) of **NI7-SO_2Cl** in $CDCl_3$.

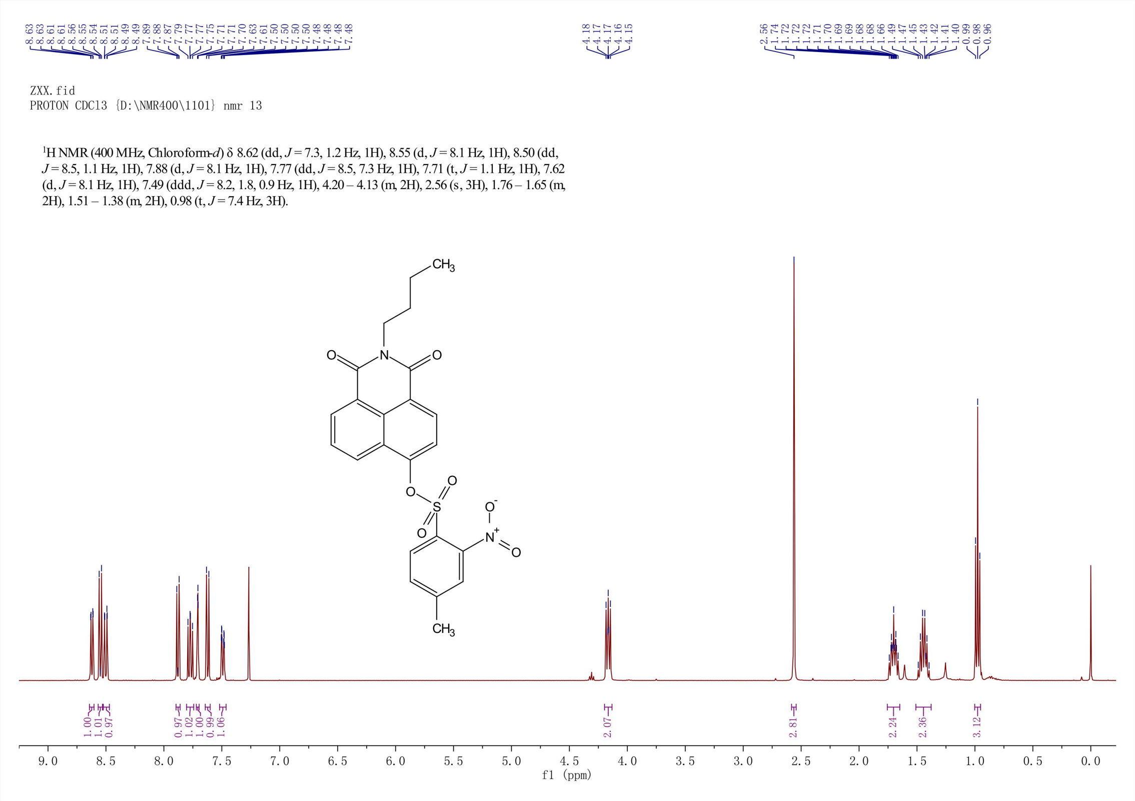

Figure S48. ^{1}H NMR spectrum (400 MHz) of **NI7** in $CDCl_3$.

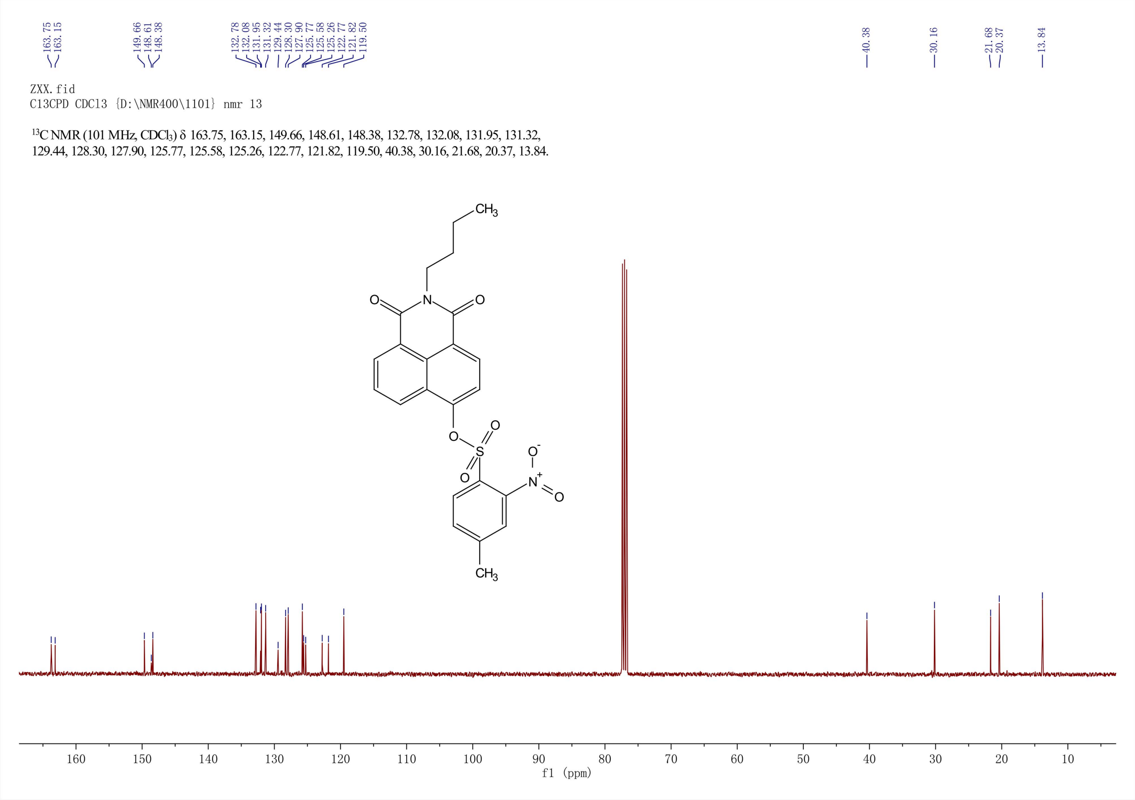

Figure S49. ^{13}C NMR spectrum (101 MHz) of **NI7** in $CDCl_3$.

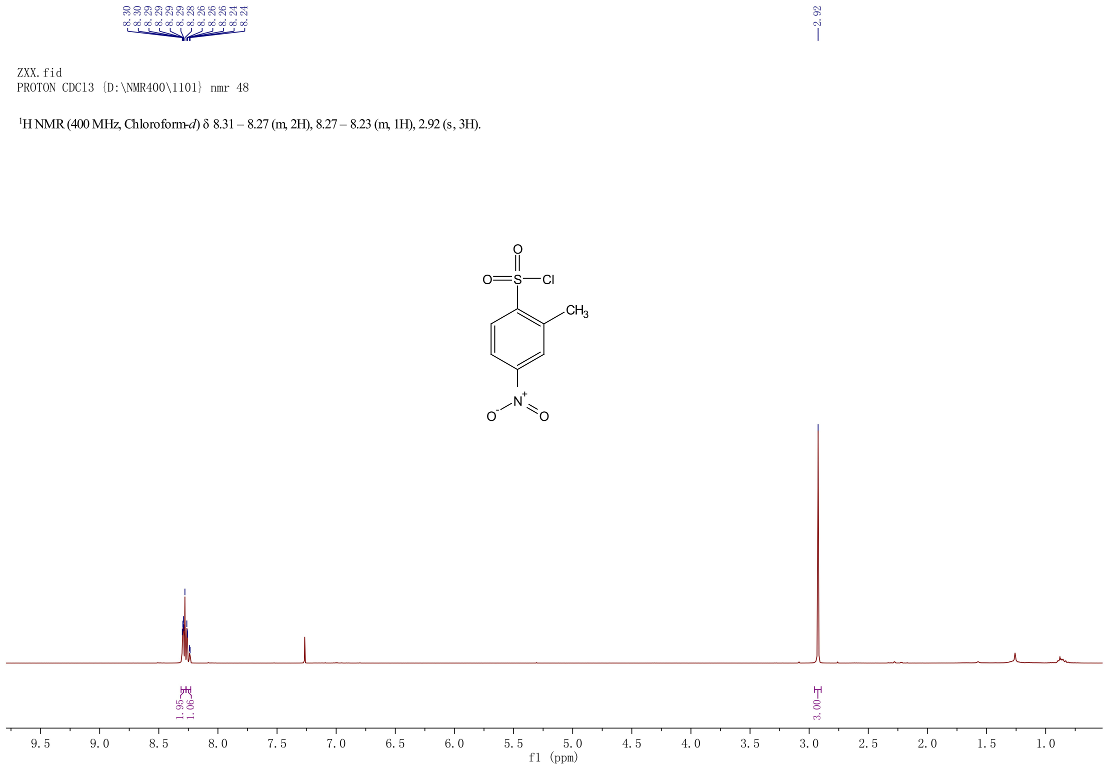

Figure S50. ^{1}H NMR spectrum (400 MHz) of **NI8-SO$_2$Cl** in $CDCl_3$.

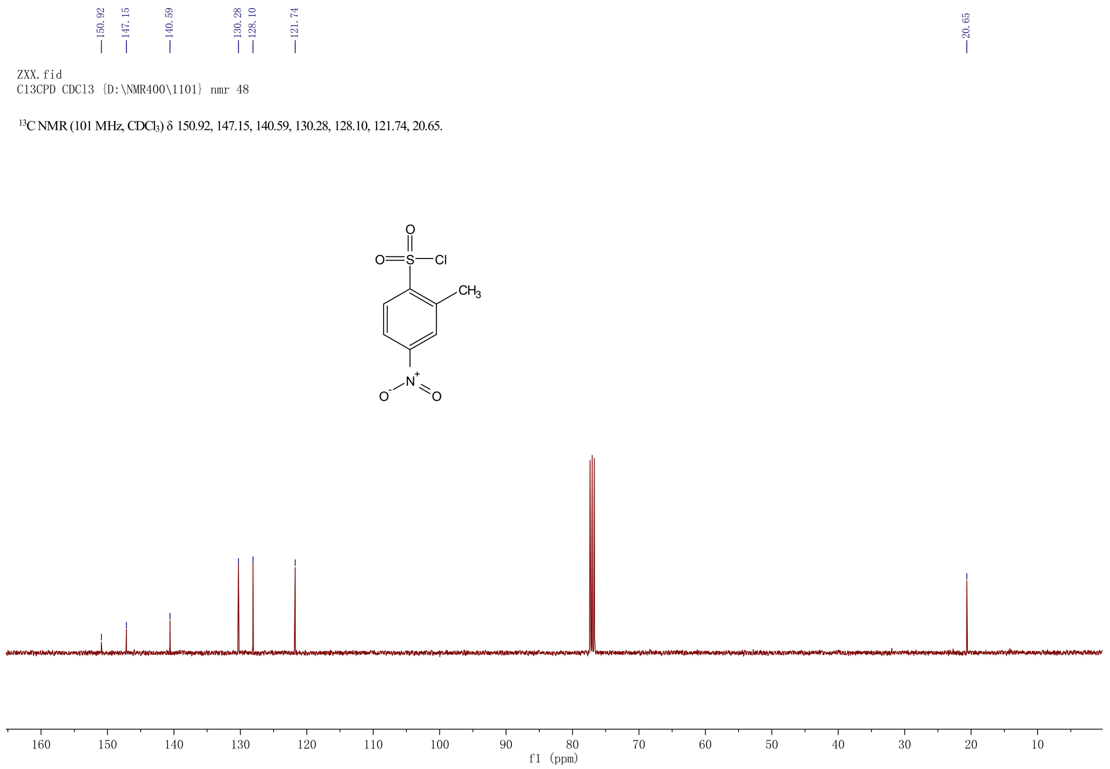

Figure S51. ^{13}C NMR spectrum (101 MHz) of **NI8-SO$_2$Cl** in $CDCl_3$.

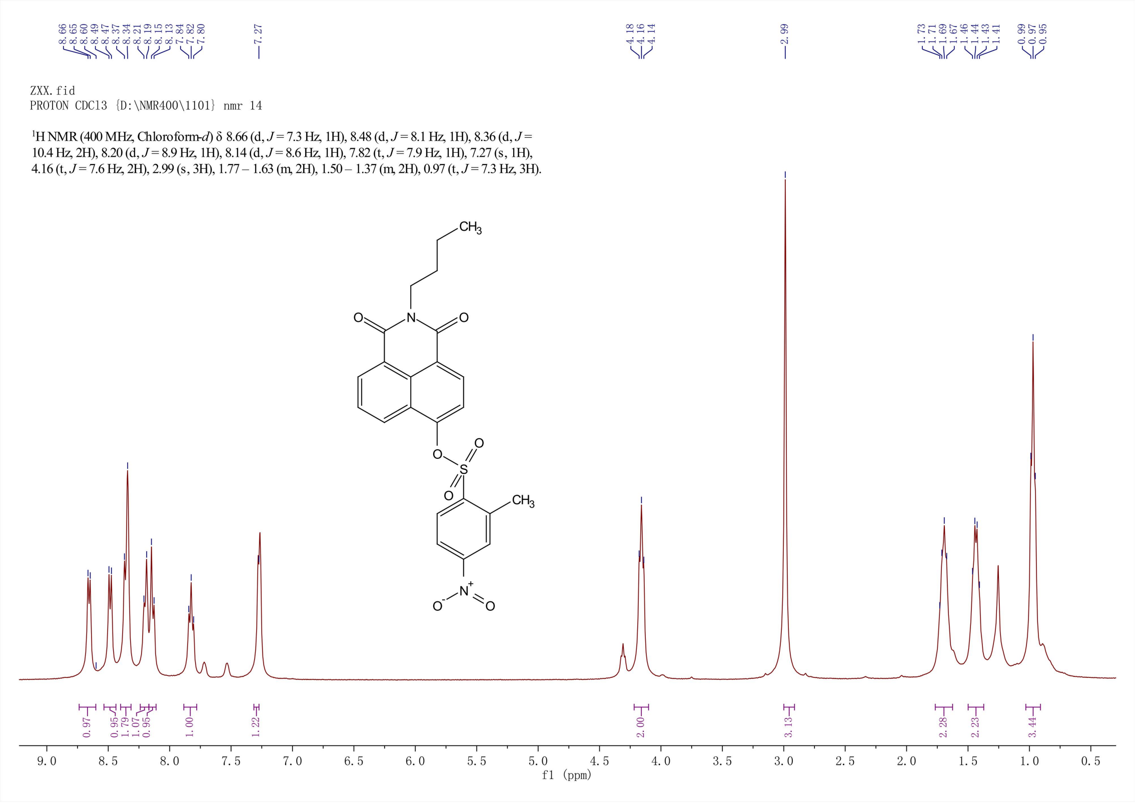

Figure S52. ^{1}H NMR spectrum (400 MHz) of **NI8** in $CDCl_3$.

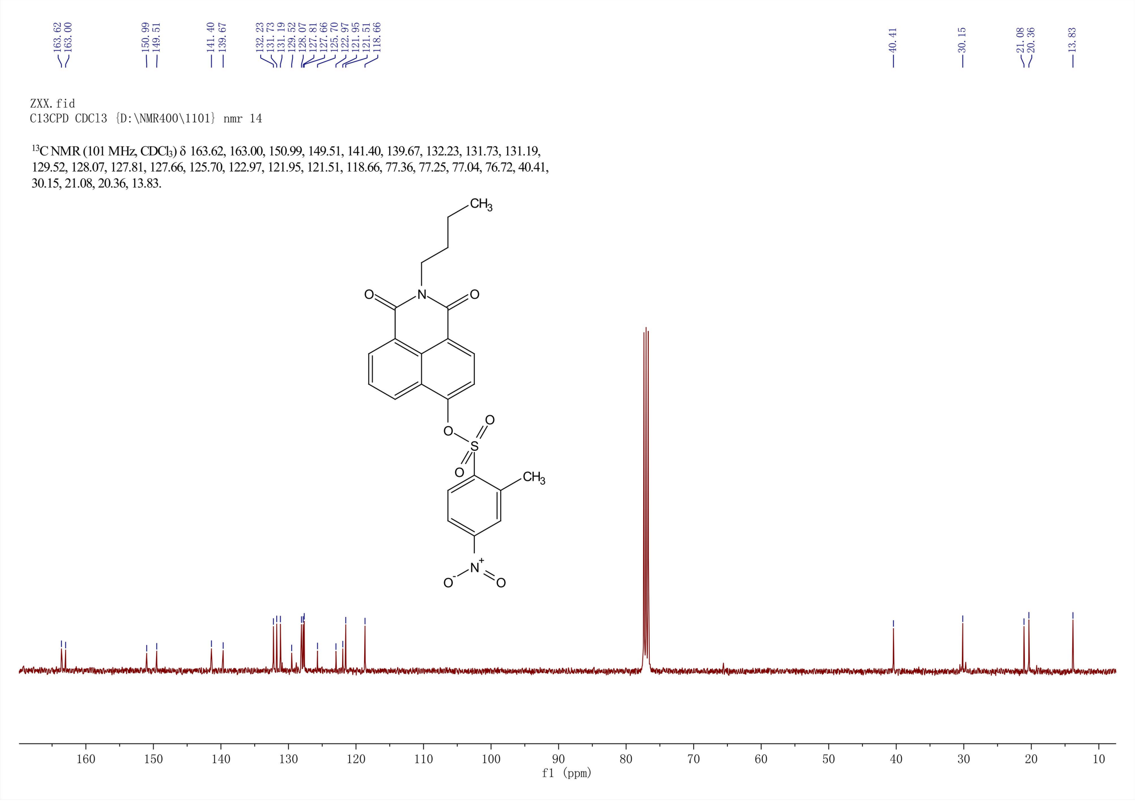

Figure S53. ^{13}C NMR spectrum (101 MHz) of **NI8** in $CDCl_3$.

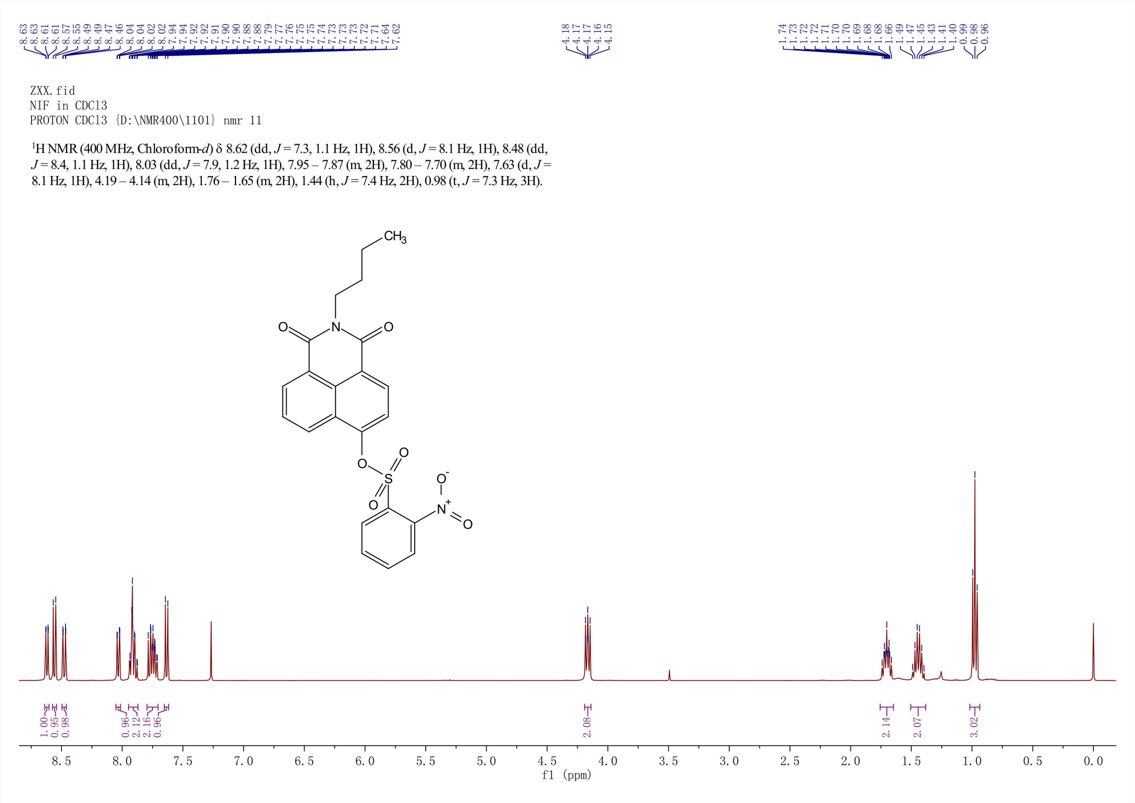

Figure S54. ^{1}H NMR spectrum (400 MHz) of **NI9** in $CDCl_3$.

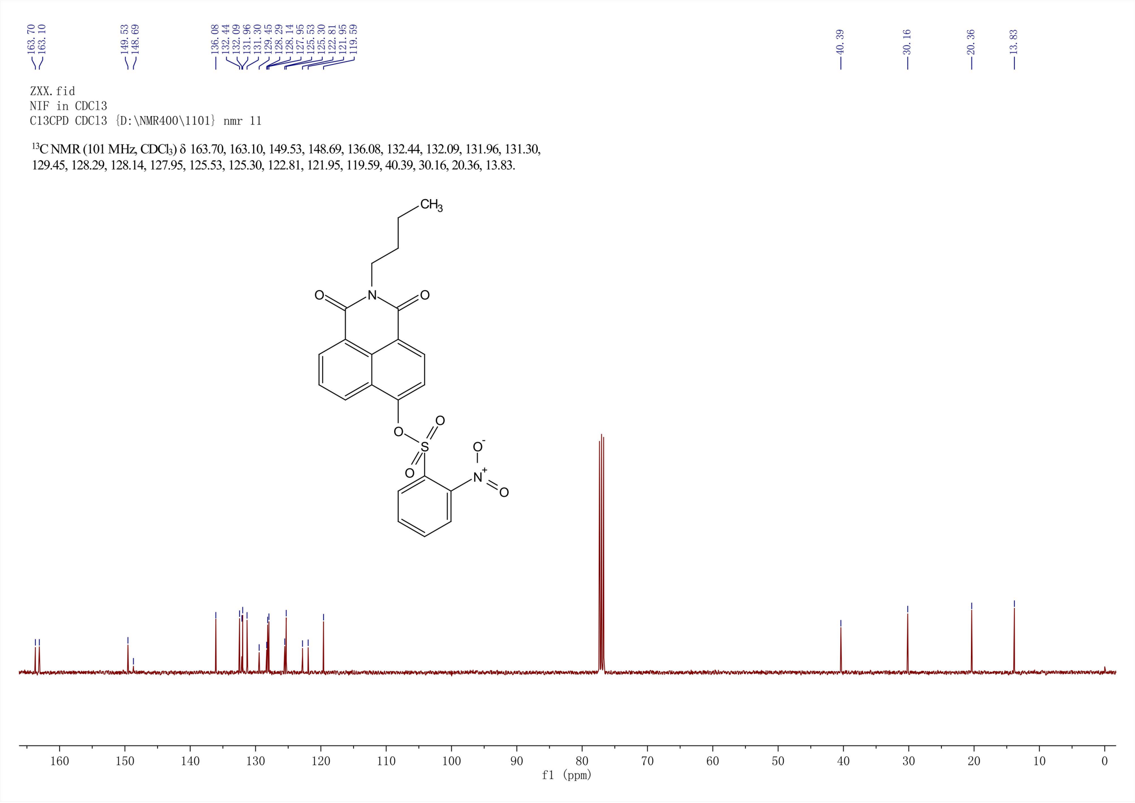

Figure S55. ^{13}C NMR spectrum (101 MHz) of **NI9** in $CDCl_3$.

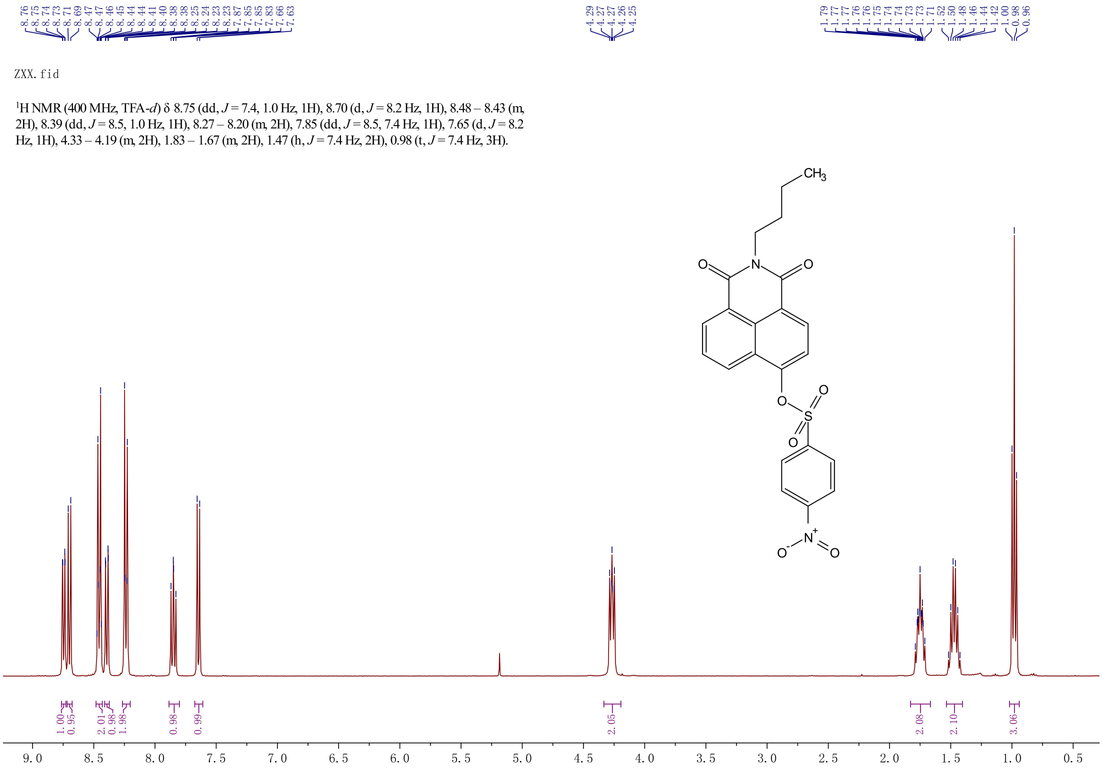

Figure S56. ^{1}H NMR spectrum (400 MHz) of **NI10** in TFA-*d*.

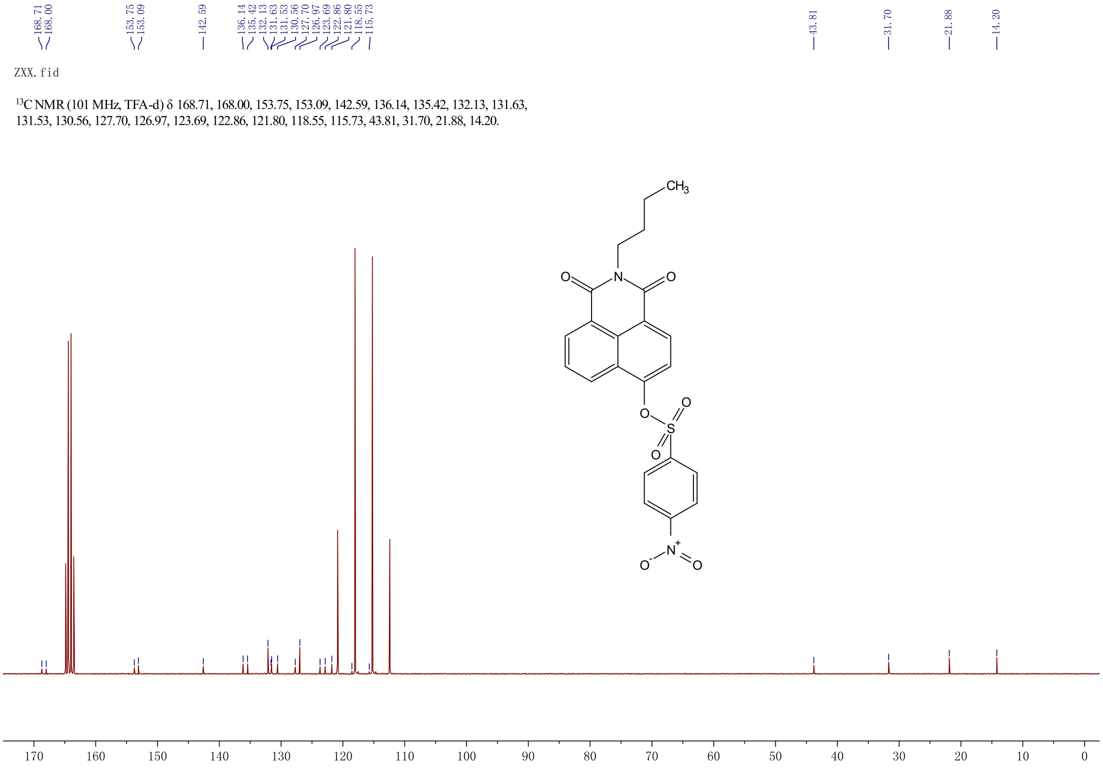

Figure S57. ^{13}C NMR spectrum (101 MHz) of **NI10** in TFA-*d*.

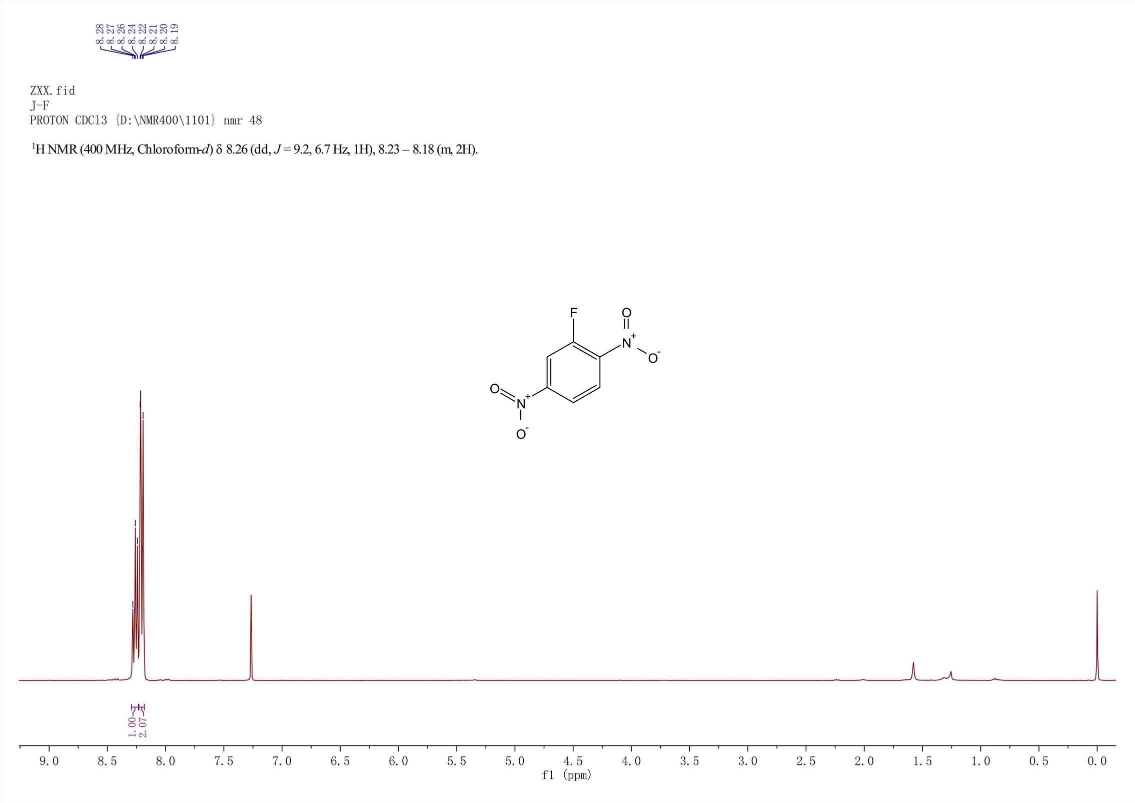

Figure S58. ^{1}H NMR spectrum (400 MHz) of **NI11-F** in $CDCl_3$.

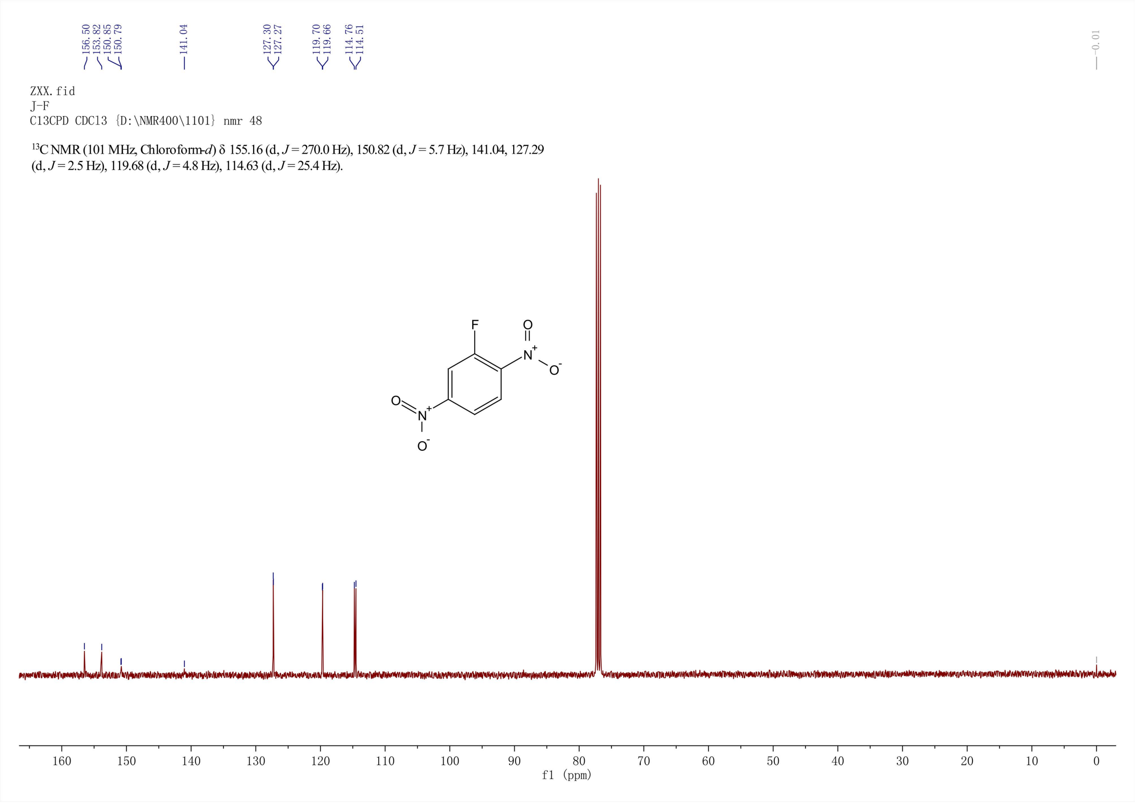

Figure S59. ^{13}C NMR spectrum (101 MHz) of **NI11-F** in $CDCl_3$.

Cartesian coordinates (angstrom) for the optimized structures of probes used for P_k^+ or ω_k calculations.

NI1

C	-1.25022900	1.66682700	2.40157900
C	-0.43067000	1.11668200	1.43754400
C	-0.98278900	0.34289500	0.38514600
C	-2.39619900	0.13969400	0.34206900
C	-3.21360300	0.71761200	1.34570000
C	-2.64602200	1.47054300	2.35737500
H	-0.81856100	2.25486600	3.20584900
H	0.64234500	1.26359000	1.47709400
C	-0.21269600	-0.25408100	-0.64998900
C	-2.97602300	-0.63432800	-0.69442200
H	-3.29688700	1.90056000	3.11139600
C	-2.17754400	-1.19622100	-1.67364400
C	-0.78257700	-1.00344000	-1.65745700
H	-2.64603400	-1.78242100	-2.45674100
H	-0.15285800	-1.43747800	-2.42513500
O	1.16589600	0.00777500	-0.67105700
C	-4.68625600	0.52110500	1.32101600
C	-4.44609600	-0.84692300	-0.74398100
N	-5.20379800	-0.27622800	0.28847100
C	-6.66468700	-0.48633100	0.25833100
C	-7.40142000	0.59740600	-0.53590300
H	-7.00215700	-0.49506000	1.29579700
H	-6.83206000	-1.46795900	-0.18743500
C	-8.91772100	0.36609500	-0.55298600
H	-7.18147600	1.57624900	-0.09109400
H	-7.01696700	0.60997300	-1.56376000
C	-9.67453100	1.44245900	-1.33769400
H	-9.12997200	-0.62097700	-0.98725900
H	-9.29207900	0.33465600	0.47987900
H	-10.75367100	1.25213100	-1.33555600
H	-9.51011200	2.43683200	-0.90504100
H	-9.34361600	1.47642300	-2.38280400
O	-5.42636700	1.02128700	2.15697500
O	-4.98215100	-1.48979800	-1.63643900
S	2.17262600	-1.25067200	-0.19035500
O	2.10321100	-2.29480000	-1.19969100
O	1.90614500	-1.49615500	1.21776700
C	3.70953800	-0.29003800	-0.37192700
C	4.88236400	-0.61922000	0.32710300
C	3.71687700	0.83020100	-1.20854900
C	6.02428300	0.16563600	0.23123200
C	4.86350900	1.61245200	-1.33831800
H	2.81922400	1.10069700	-1.74751100
C	5.99523900	1.27089500	-0.60887700
H	6.91390600	-0.08924200	0.79217700
N	5.00210800	-1.84239400	1.14961500
O	4.33978400	-2.81083100	0.79461700
O	5.78610500	-1.80729500	2.09103300
H	4.88176800	2.47933800	-1.98717400
N	7.21170600	2.10305500	-0.73027900
O	7.15048400	3.07699600	-1.47528000
O	8.19321700	1.76132100	-0.07814400

NI2

C	1.56974400	-1.04664500	2.76024300
C	0.74012200	-0.69020800	1.71687200
C	1.28525200	-0.17940000	0.51141600
C	2.70192500	-0.03518200	0.39733900

C	3.52976400	-0.41168500	1.48486000
C	2.96871700	-0.91104200	2.64619100
H	1.14353700	-1.43308200	3.68112100
H	-0.33530800	-0.78768700	1.80851700
C	0.50279900	0.20471100	-0.61225200
C	3.27421800	0.48112900	-0.79235400
H	3.62768400	-1.19079900	3.46141300
C	2.46471300	0.84656600	-1.85247600
C	1.06644200	0.70433900	-1.76764000
H	2.92749600	1.23836700	-2.75177800
H	0.42824600	0.98442500	-2.59730000
O	-0.87918400	-0.01815200	-0.55127300
C	5.00601600	-0.27549800	1.38780800
C	4.74699500	0.63121000	-0.91595700
N	5.51688600	0.26600900	0.19781200
C	6.98128400	0.41511700	0.08969500
C	7.65925400	-0.82798600	-0.49610900
H	7.35195500	0.61458000	1.09611400
H	7.16297200	1.28395800	-0.54482600
C	9.18037500	-0.65925200	-0.59884400
H	7.42453200	-1.69331600	0.13686500
H	7.24078700	-1.02583800	-1.49107400
C	9.87635700	-1.88938200	-1.19010100
H	9.40762500	0.22144100	-1.21589600
H	9.59126500	-0.45021000	0.39885400
H	10.96064500	-1.74281300	-1.25084700
H	9.69384600	-2.78035900	-0.57703800
H	9.51108700	-2.10227700	-2.20212900
O	5.75498100	-0.61191600	2.29502100
O	5.27722000	1.05384600	-1.93474000
S	-1.85198500	1.33757500	-0.31256500
O	-1.71763000	2.19495700	-1.47952100
O	-1.59713700	1.80626800	1.04051200
C	-3.41670300	0.41045200	-0.36863400
C	-4.59635900	0.89146900	0.22080300
C	-3.44626800	-0.83859100	-0.99319100
C	-5.76109000	0.13348600	0.22359300
C	-4.61943100	-1.59256300	-1.01635700
H	-2.54711600	-1.22832200	-1.45025300
C	-5.77305500	-1.11284300	-0.39940300
H	-6.64613800	0.53799700	0.69882400
N	-4.69748200	2.24468100	0.80507100
O	-3.96726000	3.10800500	0.33071500
O	-5.53224000	2.41333600	1.68787100
H	-4.62935700	-2.55490700	-1.51606900
C	-7.02707700	-1.95479000	-0.36088900
F	-8.13454200	-1.18804600	-0.36780300
F	-7.06502300	-2.71059000	0.75585600
F	-7.09063300	-2.79007500	-1.41694300

NI3

C	-0.97992000	2.36419000	1.73526700
C	-0.15547200	1.56385200	0.97138500
C	-0.69660300	0.49176900	0.21716900
C	-2.10396800	0.25141300	0.26537200
C	-2.92668000	1.09060500	1.05802700
C	-2.37005400	2.13131100	1.77893400
H	-0.55642600	3.18057200	2.31246800
H	0.91344100	1.74059600	0.94382900
C	0.07947200	-0.37452700	-0.60042300
C	-2.67227700	-0.81737700	-0.47241900
H	-3.02510000	2.75693900	2.37607000
C	-1.86832700	-1.63324700	-1.24724100
C	-0.47956700	-1.41069200	-1.31844000
H	-2.32820100	-2.44238500	-1.80418800

H	0.15412300	-2.04328700	-1.92877900
O	1.44835900	-0.09628600	-0.72755100
C	-4.39349500	0.86211500	1.12114300
C	-4.13608500	-1.06954300	-0.42721300
N	-4.89909800	-0.22561200	0.39260500
C	-6.35430400	-0.46633400	0.45247000
C	-7.12593400	0.31501200	-0.61623900
H	-6.68125900	-0.17228600	1.45106600
H	-6.50058200	-1.53956300	0.32261800
C	-8.63703700	0.06535200	-0.53233100
H	-6.92068300	1.38578000	-0.49156700
H	-6.75626900	0.02299400	-1.60753000
C	-9.42714000	0.84098800	-1.59123100
H	-8.83512600	-1.01008500	-0.64243000
H	-8.99588600	0.34344100	0.46858200
H	-10.50229100	0.64628300	-1.50794600
H	-9.27444600	1.92199000	-1.48568900
H	-9.11430900	0.55901200	-2.60396000
O	-5.13913700	1.57822500	1.77544800
O	-4.66310500	-1.96862000	-1.06836600
S	2.50045200	-1.13090200	0.08174200
O	2.45964200	-2.42323000	-0.58354300
O	2.24571100	-0.96941900	1.50423800
C	4.00131200	-0.21562300	-0.39020400
C	5.19109000	-0.29291400	0.35043400
C	3.96574100	0.62005000	-1.51022800
C	6.30254200	0.46857000	0.01474400
C	5.08211000	1.36843000	-1.87468300
H	3.05569600	0.69748900	-2.08935100
C	6.25405000	1.30407400	-1.10854700
H	7.19460000	0.39478400	0.62438100
N	5.36206900	-1.22713000	1.48404000
O	4.72057800	-2.27052500	1.43954400
O	6.16354200	-0.90847900	2.35559800
H	5.04221200	2.00914500	-2.74890700
C	7.40159900	2.08359800	-1.47275300
N	8.32735000	2.71892000	-1.77342200

NI4

C	-1.68140000	0.36545600	2.92849100
C	-0.85503900	0.18882600	1.83755000
C	-1.40521800	-0.07075600	0.55660600
C	-2.82428300	-0.15287900	0.41566300
C	-3.64884600	0.03368800	1.55377000
C	-3.08260300	0.29094400	2.78914600
H	-1.25097100	0.55881300	3.90646100
H	0.22215200	0.23603900	1.94730900
C	-0.62360600	-0.25788200	-0.61725500
C	-3.40209900	-0.42074900	-0.85076500
H	-3.73925600	0.42860500	3.64176100
C	-2.59466900	-0.60264900	-1.95896900
C	-1.19382000	-0.51725300	-1.84657500
H	-3.06152400	-0.80653200	-2.91656100
H	-0.55717800	-0.65611200	-2.71226700
O	0.76067000	-0.09075400	-0.51212800
C	-5.12700900	-0.04721900	1.43142800
C	-4.87709700	-0.50674600	-1.00253000
N	-5.64385300	-0.34385300	0.16066700
C	-7.11053700	-0.44209900	0.02942400
C	-7.76952200	0.90635500	-0.27912900
H	-7.48407100	-0.84228500	0.97327000
H	-7.30683400	-1.15583000	-0.77204900
C	-9.29299500	0.78445700	-0.40904300
H	-7.52176900	1.61524300	0.52109000
H	-7.34901400	1.30379700	-1.21178100

C	-9.97294800	2.12204200	-0.71818500
H	-9.53344700	0.06014100	-1.19993800
H	-9.70516100	0.37103900	0.52218800
H	-11.05912000	2.00601600	-0.80553700
H	-9.77832400	2.85713000	0.07226200
H	-9.60551200	2.54412900	-1.66152500
O	-5.87354100	0.12969000	2.38481100
O	-5.41219600	-0.71215400	-2.08384900
S	1.68862900	-1.50935600	-0.54316200
O	1.57442900	-2.06641100	-1.88165300
O	1.33351100	-2.24651600	0.65871100
C	3.29142000	-0.66192400	-0.32092200
C	4.46312500	-1.35151000	0.06060700
C	3.34487500	0.70418700	-0.52952100
C	5.62047700	-0.63181500	0.31135600
C	4.53657100	1.46673400	-0.35813500
H	2.45131700	1.20487100	-0.86547000
C	5.65321700	0.74699800	0.15561400
H	6.50162000	-1.15311100	0.66292400
N	4.54098900	-2.80508400	0.18032700
O	3.63200800	-3.45674300	-0.33154900
O	5.51828200	-3.28259700	0.75327200
N	6.85002700	1.40815900	0.69000700
O	6.68856200	2.48885900	1.25991500
O	7.92356600	0.81635400	0.59276700
N	4.55837800	2.78034600	-0.71808900
C	3.31582500	3.51712500	-0.93519400
H	2.89323500	3.34074000	-1.93470100
H	2.57229200	3.25391400	-0.17942000
H	3.52873600	4.58369600	-0.83391200
C	5.77241200	3.46468900	-1.15974200
H	5.52557600	4.06304900	-2.04288500
H	6.18625400	4.11391300	-0.38211500
H	6.53443700	2.73951900	-1.45340200

NI5

C	-1.52874300	0.41781100	2.90778900
C	-0.70979500	0.26904600	1.80728000
C	-1.26709700	0.00572500	0.53023600
C	-2.68541400	-0.10726700	0.40283800
C	-3.50222600	0.05178400	1.55049700
C	-2.92930900	0.31181900	2.78208700
H	-1.09298100	0.61593700	3.88238000
H	0.36673700	0.34669400	1.90649100
C	-0.49659000	-0.15350600	-0.65392200
C	-3.27028600	-0.37696200	-0.85993900
H	-3.58003300	0.42888600	3.64226100
C	-2.47164700	-0.52945600	-1.97854100
C	-1.07169000	-0.41262200	-1.88032000
H	-2.94411500	-0.73362000	-2.93325900
H	-0.44201200	-0.52464500	-2.75500400
O	0.88833700	0.04257300	-0.55835500
C	-4.97995700	-0.05836300	1.44194300
C	-4.74517500	-0.49402900	-0.99741100
N	-5.50355600	-0.35392800	0.17372900
C	-6.96976000	-0.47576000	0.05463800
C	-7.65189600	0.86344000	-0.24416000
H	-7.32871300	-0.88475600	1.00026800
H	-7.16142400	-1.19003400	-0.74746600
C	-9.17432000	0.71893700	-0.36257900
H	-7.40853900	1.57357500	0.55634600
H	-7.24489800	1.27022500	-1.17871400
C	-9.87584900	2.04683300	-0.66549900
H	-9.40998100	-0.00802600	-1.15248400
H	-9.57334800	0.29865500	0.57126500

H	-10.96079100	1.91508700	-0.74515500
H	-9.68622900	2.78404900	0.12416100
H	-9.52124400	2.47481100	-1.61104700
O	-5.71954400	0.09504300	2.40445700
O	-5.28540700	-0.70455000	-2.07498300
S	1.84772000	-1.33716300	-0.67211200
O	1.80974900	-1.79154400	-2.05294900
O	1.49352700	-2.18934400	0.45101900
C	3.41438300	-0.46419300	-0.33552500
C	4.56036500	-1.12991900	0.13657000
C	3.46268200	0.91642500	-0.50234200
C	5.70961000	-0.42080800	0.45676300
C	4.62867600	1.64867100	-0.22521100
H	2.57985200	1.45903300	-0.81145100
C	5.75060800	0.95131600	0.24725100
H	6.57372800	-0.94373000	0.84698400
N	4.64098700	-2.59631800	0.25653500
O	3.90141200	-3.25180900	-0.47050200
O	5.46693600	-3.05226900	1.04053900
N	7.02365300	1.64230400	0.51453200
O	7.32701900	2.57306000	-0.22515600
O	7.70330600	1.21014400	1.44140700
O	4.62133800	2.99663000	-0.29520600
C	4.59212000	3.58664000	-1.60815100
H	3.75735400	3.19437400	-2.20009500
H	4.45807800	4.65609900	-1.44575300
H	5.54192600	3.40074800	-2.11743100

NI6

C	-1.10998800	-2.46799000	2.05823500
C	-0.16978600	-1.62296200	1.50421100
C	-0.56655800	-0.61816800	0.58462900
C	-1.94744000	-0.49990700	0.23785400
C	-2.89060200	-1.38006400	0.82583200
C	-2.47431300	-2.34644400	1.72389900
H	-0.79741300	-3.23791600	2.75718700
H	0.88062500	-1.72597800	1.75066800
C	0.33729500	0.29865800	-0.02167000
C	-2.36980900	0.48787900	-0.68690100
H	-3.21894400	-3.00566400	2.15765800
C	-1.44677100	1.34507600	-1.25863300
C	-0.08304100	1.25378300	-0.92348400
H	-1.79544600	2.09066800	-1.96504900
H	0.64264800	1.92303100	-1.37104500
O	1.67116300	0.27281200	0.38981100
C	-4.33194200	-1.27292400	0.48476000
C	-3.80392000	0.61603200	-1.04914300
N	-4.69040400	-0.29544700	-0.45742600
C	-6.11749600	-0.18476400	-0.81568100
C	-6.87985300	0.79002100	0.08806300
H	-6.53950100	-1.18786800	-0.73847500
H	-6.15637900	0.14816300	-1.85409000
C	-8.35995300	0.89659500	-0.29987500
H	-6.79308900	0.45331600	1.12903800
H	-6.40791200	1.77862900	0.02246200
C	-9.13910400	1.87165100	0.58857900
H	-8.43879700	1.21469700	-1.34897800
H	-8.82304600	-0.09867800	-0.24484200
H	-10.19229900	1.92748300	0.29092200
H	-9.10546000	1.56224500	1.64032100
H	-8.72068800	2.88367900	0.52700600
O	-5.18054100	-1.99669500	0.98849600
O	-4.20871600	1.47023200	-1.82711100
S	2.79259500	-0.53552200	-0.58734000
O	2.46204200	-0.22626100	-1.96942600

O	2.86520400	-1.89768700	-0.07935300
C	4.24166100	0.40241900	-0.01331300
C	5.55325700	-0.08363300	-0.15876300
C	4.05028600	1.61963400	0.63715700
C	6.62973600	0.62550600	0.36117500
C	5.12907700	2.36008300	1.14806300
H	3.04278200	1.99247200	0.76727800
C	6.41787400	1.84075400	1.00686300
H	7.62469500	0.21535400	0.23746100
H	7.26894900	2.39097700	1.39762300
N	5.87130300	-1.30581600	-0.91534000
O	5.07504900	-1.64805900	-1.78484100
O	6.92746300	-1.87514500	-0.65290000
C	4.87690900	3.67181200	1.84936100
H	4.35505100	3.51279400	2.80107000
H	5.81227300	4.19603200	2.06395400
H	4.24772300	4.33190600	1.24191800

NI7

C	-0.87385800	2.82719500	0.87227200
C	-0.04158600	1.83810000	0.38917000
C	-0.56929000	0.57398000	0.02271000
C	-1.97089600	0.33991600	0.16606900
C	-2.80225200	1.37500300	0.66346100
C	-2.25856500	2.59925100	1.00844400
H	-0.46043700	3.79081700	1.15449600
H	1.02380200	2.01111900	0.29115900
C	0.22004000	-0.49364700	-0.48823800
C	-2.52463000	-0.91706400	-0.18478800
H	-2.91996500	3.37198200	1.38602100
C	-1.70994200	-1.92419900	-0.67006700
C	-0.32691000	-1.71391600	-0.82851900
H	-2.15819200	-2.87654900	-0.93205900
H	0.31458000	-2.49845200	-1.21196400
O	1.57596700	-0.24660900	-0.71834100
C	-4.26289600	1.15590200	0.81846200
C	-3.98143500	-1.16450600	-0.03991600
N	-4.75344300	-0.11530400	0.48130700
C	-6.20158200	-0.34982200	0.63940600
C	-7.00057800	0.01805000	-0.61539500
H	-6.52667000	0.25229300	1.48917200
H	-6.32511100	-1.40817200	0.87382900
C	-8.50303900	-0.23154600	-0.43365600
H	-6.82650300	1.07573600	-0.85084100
H	-6.62674200	-0.57260000	-1.46144900
C	-9.32110100	0.13221200	-1.67691800
H	-8.66831600	-1.28892300	-0.18327000
H	-8.86650600	0.34856000	0.42621500
H	-10.38912800	-0.05731700	-1.52090100
H	-9.20387600	1.19264200	-1.93125600
H	-9.00143700	-0.45474200	-2.54658800
O	-5.01733300	2.03067700	1.22298200
O	-4.49922600	-2.22829900	-0.35368200
S	2.66438800	-0.94398500	0.37383900
O	2.60403500	-2.38695000	0.19423700
O	2.42392200	-0.30798100	1.66043900
C	4.13933400	-0.24611700	-0.41490800
C	5.34610800	-0.05043700	0.27668900
C	4.07946200	0.15129400	-1.75110400
C	6.43651000	0.55491100	-0.33395600
C	5.18651900	0.73624500	-2.36841000
H	3.16035500	0.02032800	-2.30612500
C	6.37804100	0.95873500	-1.67361600
H	7.33715200	0.69276300	0.25397100
N	5.55410600	-0.53792200	1.65400200

O	4.91686700	-1.53010700	1.99373400
O	6.37923500	0.05292100	2.34488700
H	5.10928800	1.03057900	-3.41133000
C	7.57244100	1.60556000	-2.32953900
H	7.83825000	2.54196900	-1.82492400
H	8.45115200	0.95201600	-2.28163400
H	7.37637100	1.83246100	-3.38107100

NI8

C	1.03932900	2.64390700	-1.34165600
C	0.20128900	1.69635000	-0.79048100
C	0.73384200	0.50083300	-0.24447600
C	2.14647700	0.29191300	-0.27810700
C	2.98313200	1.28328600	-0.84987900
C	2.43448700	2.44094000	-1.37107900
H	0.62281100	3.55512600	-1.76023700
H	-0.87133900	1.85094500	-0.77425800
C	-0.05718500	-0.52243700	0.34533500
C	2.70579500	-0.89716400	0.25367700
H	3.10002600	3.18163600	-1.80177600
C	1.88733500	-1.86245400	0.81153900
C	0.49244100	-1.67583500	0.86354100
H	2.34051500	-2.76233300	1.21311500
H	-0.15387300	-2.42556300	1.30446600
O	-1.43586400	-0.29525400	0.46317600
C	4.45538700	1.08950500	-0.89424800
C	4.17463400	-1.11787700	0.22182500
N	4.95304800	-0.11655900	-0.37670700
C	6.41341400	-0.32638000	-0.42166600
C	7.12525400	0.19827900	0.82975000
H	6.77693200	0.18829800	-1.31225400
H	6.57379400	-1.40021900	-0.53021900
C	8.64067600	-0.03071500	0.76683300
H	6.91682400	1.27058000	0.93529800
H	6.71110300	-0.30507800	1.71255500
C	9.37245000	0.48421300	2.01041600
H	8.84093900	-1.10419700	0.64258500
H	9.04616700	0.46331000	-0.12731500
H	10.45167800	0.30818500	1.93890100
H	9.21808400	1.56204500	2.14264200
H	9.01202800	-0.01625500	2.91738700
O	5.21186100	1.93057700	-1.36061900
O	4.69438500	-2.12233200	0.68922700
S	-2.42969800	-1.12972200	-0.59532600
O	-2.38617300	-2.55025200	-0.25938100
O	-2.11626900	-0.68965500	-1.95121900
C	-3.99722600	-0.42242900	-0.06072300
C	-5.14091100	-0.73862500	-0.82539700
C	-4.05862000	0.37204300	1.08761100
C	-6.35692500	-0.20388200	-0.38910800
C	-5.28085200	0.89415300	1.50120500
H	-3.16073400	0.58285800	1.65279800
C	-6.40957500	0.59459500	0.74848200
H	-7.27166000	-0.40741700	-0.93263800
H	-5.36397000	1.51528900	2.38388000
N	-7.71500300	1.14097200	1.17287700
O	-7.73186600	1.84779000	2.17815900
O	-8.69871200	0.85467600	0.49414600
C	-5.10448800	-1.60420000	-2.06265700
H	-4.53839400	-1.12114000	-2.86543300
H	-4.62283300	-2.56665700	-1.86075000
H	-6.11910800	-1.79729000	-2.41906200

NI9

C	-0.62367200	2.96940200	0.23708400
C	0.20583500	1.91453000	-0.08465400
C	-0.31363300	0.59888700	-0.18288600
C	-1.70394300	0.38250300	0.06201400
C	-2.53310300	1.48525700	0.38781400
C	-1.99798800	2.75800000	0.47118600
H	-0.21652700	3.97309000	0.31424200
H	1.26300200	2.07620100	-0.26079100
C	0.47255000	-0.53809500	-0.51798200
C	-2.24837600	-0.92391900	-0.01854600
H	-2.65767700	3.58195500	0.72226500
C	-1.43649400	-1.99635500	-0.34072700
C	-0.06547800	-1.80607600	-0.59908700
H	-1.87757300	-2.98562900	-0.39808200
H	0.57220700	-2.64244900	-0.85959000
O	1.81400000	-0.32084100	-0.84714700
C	-3.98336900	1.28523800	0.64023700
C	-3.69296200	-1.15428600	0.23561600
N	-4.46282300	-0.03219400	0.57663900
C	-5.89930500	-0.24807600	0.83720200
C	-6.75025000	-0.15750800	-0.43394400
H	-6.20561100	0.51175600	1.55759600
H	-5.99194900	-1.23705600	1.28876900
C	-8.23945800	-0.38894500	-0.14811900
H	-6.61003800	0.83217900	-0.88718300
H	-6.39213600	-0.90272700	-1.15548800
C	-9.10594800	-0.30949300	-1.40907600
H	-8.37010100	-1.37261700	0.32443200
H	-8.58908100	0.35271900	0.58377700
H	-10.16385400	-0.47628700	-1.17689800
H	-9.02002800	0.67402100	-1.88696400
H	-8.80277600	-1.06368900	-2.14543400
O	-4.73749300	2.21424300	0.89718600
O	-4.20291400	-2.26407100	0.15630300
S	2.95827000	-0.85347600	0.27697900
O	2.97302800	-2.30787400	0.23442100
O	2.71433200	-0.10902800	1.50283300
C	4.38216500	-0.16195800	-0.61381000
C	5.58901900	0.15276900	0.03076400
C	4.26704100	0.10121500	-1.98106000
C	6.63799800	0.75201600	-0.65997300
C	5.32883800	0.67746500	-2.68148800
H	3.34169300	-0.12868900	-2.49160900
C	6.50952500	1.01237300	-2.02243600
H	7.54367200	0.99138500	-0.11586800
N	5.84507400	-0.19270200	1.44223600
O	5.26614400	-1.17913900	1.88631700
O	6.65060800	0.50148500	2.05530200
H	5.22028200	0.86949800	-3.74442200
H	7.33285000	1.46835300	-2.56303700

NI10

C	0.74624800	2.84968300	-0.49153200
C	-0.05527200	1.74439700	-0.29080100
C	0.52116800	0.45599100	-0.15583300
C	1.94105900	0.32114300	-0.22876200
C	2.73994000	1.47388300	-0.43502800
C	2.14826600	2.71743400	-0.56355300
H	0.29622700	3.83249900	-0.59499300
H	-1.13293800	1.84634700	-0.23123200
C	-0.23419100	-0.72950400	0.05819500
C	2.54504300	-0.95448300	-0.09719200
H	2.78597200	3.58118700	-0.71958100

C	1.76178800	-2.07571900	0.10762700
C	0.36026300	-1.96709800	0.19227000
H	2.24799200	-3.03993100	0.20972500
H	-0.25472000	-2.84304900	0.36129700
O	-1.62059700	-0.58664800	0.20585700
C	4.21932600	1.35824500	-0.51131300
C	4.02124400	-1.09957500	-0.16760800
N	4.76233500	0.06937100	-0.39693400
C	6.23115100	-0.05932100	-0.46760000
C	6.90062500	0.08392500	0.90334000
H	6.58312300	0.71735700	-1.14817600
H	6.44064000	-1.04103800	-0.89476500
C	8.42559800	-0.05803200	0.81776100
H	6.64274600	1.06404600	1.32431300
H	6.49543800	-0.68024500	1.57885900
C	9.11258000	0.08128500	2.18001500
H	8.67578500	-1.03467700	0.38002100
H	8.82341600	0.69941900	0.12780700
H	10.19958200	-0.02515100	2.09073800
H	8.90877700	1.06151300	2.62779700
H	8.75877800	-0.68399500	2.88154500
O	4.94387500	2.33170700	-0.66893200
O	4.57897700	-2.18077100	-0.03540400
S	-2.60193200	-1.25122100	-0.98144700
O	-2.59720900	-2.70227800	-0.83067100
O	-2.27997100	-0.62216900	-2.25668200
C	-4.14171000	-0.59265800	-0.34771500
C	-4.65074600	0.58096000	-0.90584400
C	-4.80247200	-1.28208500	0.67096700
C	-5.85280100	1.09021000	-0.41986500
C	-6.00238200	-0.77185900	1.15901900
H	-4.38930200	-2.20367700	1.06536100
C	-6.50106600	0.40564000	0.60531600
H	-6.29050100	1.99582400	-0.82082300
H	-6.55208300	-1.26847000	1.94873400
N	-7.77570600	0.94795700	1.12367000
O	-8.33183500	0.32271500	2.02303400
O	-8.19286600	1.98868200	0.62148200
H	-4.11936800	1.07561000	-1.71127300

NI11

C	-1.07105600	0.14064800	2.96911200
C	-0.23228000	0.13615100	1.87358100
C	-0.76200500	-0.01308000	0.56674900
C	-2.17325500	-0.16267500	0.40384900
C	-3.01090700	-0.15264600	1.54736000
C	-2.46484700	-0.00150900	2.80893700
H	-0.65641100	0.25310800	3.96631500
H	0.83896300	0.24315900	1.99895600
C	0.02880600	-0.02044800	-0.61433100
C	-2.73043300	-0.32034800	-0.89000700
H	-3.13127600	0.00346000	3.66501200
C	-1.91219500	-0.32414500	-2.00471800
C	-0.51896600	-0.16734700	-1.87108300
H	-2.36395500	-0.44305300	-2.98355500
H	0.12554400	-0.16147600	-2.74209100
O	1.40654000	0.20864100	-0.47306800
C	-4.48234500	-0.30109000	1.40259700
C	-4.19779000	-0.47726800	-1.06384500
N	-4.97734800	-0.48189600	0.10173100
C	-6.43660100	-0.63701400	-0.05771100
C	-7.15249400	0.70173100	-0.26704300
H	-6.80062400	-1.12868300	0.84542900
H	-6.59198100	-1.29138300	-0.91694100
C	-8.66723900	0.52436500	-0.43024600

H	-6.94614400	1.35436200	0.59087900
H	-6.73882900	1.19046000	-1.15813900
C	-9.40001600	1.85130500	-0.65352100
H	-8.86503800	-0.14946500	-1.27570200
H	-9.07360800	0.02670900	0.46143100
H	-10.47885500	1.69651100	-0.76744200
H	-9.24817400	2.53461900	0.19089700
H	-9.03759400	2.35541900	-1.55759100
O	-5.23949000	-0.27280700	2.36320700
O	-4.71493900	-0.59902800	-2.16610300
S	2.41252700	-1.11362000	-0.71712700
O	2.39791600	-1.44180400	-2.13356700
O	2.10567900	-2.08933300	0.31652900
C	3.93704600	-0.21867600	-0.28661400
C	5.08287200	-0.87320200	0.19151300
C	3.96181700	1.17006700	-0.40871700
C	6.21639400	-0.16316900	0.57896800
C	5.11291000	1.86017100	-0.04098600
H	3.10193800	1.71907800	-0.76534200
C	6.24175500	1.22251500	0.45923100
H	7.06837100	-0.71150100	0.96227100
N	5.18289300	-2.34743600	0.25361500
O	4.60400700	-2.97549700	-0.62499600
O	5.87565500	-2.82078700	1.14760200
H	7.11179700	1.80249000	0.74038300
N	5.12410700	3.33352500	-0.18026400
O	4.10953400	3.86615700	-0.61957300
O	6.15045700	3.91804900	0.15437400

NI12

C	1.03329800	2.77313700	-0.48236800
C	0.22597200	1.67152000	-0.28426500
C	0.80184700	0.38069400	-0.15633400
C	2.22285100	0.24184100	-0.23341000
C	3.02457000	1.39338700	-0.43652000
C	2.43452300	2.63846100	-0.55864800
H	0.58393800	3.75661800	-0.58027100
H	-0.84991800	1.78482500	-0.22516000
C	0.05264800	-0.80928000	0.05157900
C	2.82626900	-1.03466300	-0.10576000
H	3.07344000	3.50169700	-0.71285600
C	2.04559900	-2.15812300	0.09564600
C	0.64492700	-2.04718700	0.17848300
H	2.53270800	-3.12224700	0.19282900
H	0.02677100	-2.92263300	0.33846800
O	-1.34187500	-0.69953700	0.22014500
C	4.50261500	1.27581400	-0.51643800
C	4.30327100	-1.18182800	-0.17848000
N	5.04502200	-0.01517900	-0.40631000
C	6.51339600	-0.14502900	-0.48056000
C	7.18641000	-0.00337600	0.88881700
H	6.86424300	0.63220200	-1.16106800
H	6.72125400	-1.12645100	-0.90928600
C	8.71122400	-0.14413200	0.79891200
H	6.92902500	0.97602900	1.31164500
H	6.78346400	-0.76864500	1.56448400
C	9.40190300	-0.00660800	2.15948300
H	8.96106500	-1.11981400	0.35874300
H	9.10654000	0.61485000	0.10922500
H	10.48877200	-0.11187700	2.06703100
H	9.19843300	0.97263600	2.60953900
H	9.05071900	-0.77341200	2.86069500
O	5.23015300	2.24702000	-0.67287800
O	4.85768200	-2.26548300	-0.04912600
S	-2.31090400	-1.06756100	-1.07872700

O	-2.03205700	-2.43269100	-1.50830700
O	-2.30259700	0.03526100	-2.02824300
C	-3.87915300	-1.08130000	-0.17984900
C	-4.59341600	0.07951800	0.13340600
C	-4.40169300	-2.33841000	0.13200000
C	-5.83187000	-0.04522500	0.76833600
C	-5.63845800	-2.44565900	0.76495200
H	-3.83361600	-3.22039900	-0.14115100
C	-6.35605500	-1.29755600	1.08163200
N	-4.06508000	1.41403100	-0.25176500
O	-4.60348700	1.95061200	-1.20384000
O	-3.12940900	1.83462200	0.41439400
H	-6.04244000	-3.42274300	1.00750200
H	-7.32241700	-1.34282100	1.56894000
N	-6.61818400	1.14045100	1.17174100
O	-6.01836800	2.20417700	1.27899400
O	-7.81178000	0.96377800	1.39398100

NI13

C	1.03207700	2.70445700	1.64362900
C	0.13939200	1.76077100	1.17796200
C	0.59782300	0.66253200	0.40596100
C	1.99269600	0.55232700	0.11637000
C	2.88556900	1.53742500	0.60840200
C	2.40920500	2.59469800	1.36212500
H	0.67164500	3.54347500	2.23110300
H	-0.91980100	1.85416500	1.38639600
C	-0.24777700	-0.35962000	-0.10579900
C	2.47866200	-0.53317000	-0.65526300
H	3.11697900	3.33229900	1.72550600
C	1.60730700	-1.49753300	-1.12796400
C	0.22876200	-1.41569100	-0.85123400
H	2.00563000	-2.32012800	-1.71187900
H	-0.45863300	-2.17443900	-1.20518700
O	-1.61003800	-0.32048200	0.24318400
C	4.33987000	1.44393400	0.32108700
C	3.92838900	-0.65055700	-0.95979300
N	4.76238400	0.36666900	-0.47317400
C	6.20364500	0.26834900	-0.77516700
C	6.97751100	-0.52167500	0.28552900
H	6.58143900	1.28979600	-0.84125100
H	6.28826400	-0.21547500	-1.74930700
C	8.47454400	-0.60436800	-0.03819800
H	6.83601100	-0.04016300	1.26143500
H	6.55685600	-1.53297900	0.35436200
C	9.26710700	-1.39123600	1.01051800
H	8.60846400	-1.07105500	-1.02427400
H	8.88484300	0.41194500	-0.12075500
H	10.33225900	-1.43310100	0.75632200
H	9.17823200	-0.93004400	2.00167600
H	8.90216700	-2.42242800	1.09054400
O	5.14502800	2.26201800	0.74534200
O	4.38664300	-1.58315300	-1.60607000
S	-2.68542000	0.30041900	-0.87633000
O	-2.48427500	-0.32829000	-2.16731200
O	-2.67484300	1.74654400	-0.71074200
C	-4.16753100	-0.33158100	-0.00180600
C	-5.31624600	0.46867000	0.07389500
C	-4.17680700	-1.50931900	0.75670100
C	-6.38561000	0.15726400	0.90835000
C	-5.23290200	-1.83626300	1.60515700
C	-6.33977100	-0.99773300	1.68326100
H	-7.23752000	0.82648500	0.93235100
N	-5.50805400	1.64985100	-0.80036000
O	-5.15475700	1.52766300	-1.96702000

O	-6.07111200	2.62023900	-0.30762000
H	-5.16872500	-2.74747600	2.18844300
H	-7.16823100	-1.24870000	2.33718100
N	-3.10080800	-2.52174400	0.66982400
O	-2.70223700	-3.00092900	1.72416500
O	-2.74607300	-2.84890900	-0.45739600

NI14

C	0.83853600	2.88127800	1.12721500
C	-0.03161700	1.84601600	0.85284800
C	0.45341100	0.62876000	0.30931000
C	1.85223400	0.49395600	0.04988400
C	2.72178100	1.57483700	0.34233600
C	2.21919200	2.74838600	0.87441600
H	0.45730400	3.81051600	1.53969500
H	-1.09340900	1.95587600	1.03891600
C	-0.36821000	-0.49067500	0.00257500
C	2.36521700	-0.71046400	-0.49439900
H	2.90918100	3.55785300	1.08831600
C	1.51659200	-1.76607800	-0.77349600
C	0.13461200	-1.66110700	-0.52232000
H	1.93477800	-2.67849500	-1.18446800
H	-0.53139900	-2.49169500	-0.72185500
O	-1.73456100	-0.41538800	0.33382000
C	4.17966200	1.45790200	0.08242900
C	3.81911900	-0.85498600	-0.76452600
N	4.62935000	0.25455400	-0.48303700
C	6.07385100	0.12824000	-0.75755700
C	6.85348500	-0.44540200	0.43033200
H	6.43471200	1.12758100	-1.00515100
H	6.17257200	-0.52140800	-1.62866400
C	8.35133100	-0.57491300	0.12639900
H	6.70716900	0.20672800	1.30086700
H	6.44095700	-1.43001800	0.68446800
C	9.14949300	-1.14897100	1.30136600
H	8.48882700	-1.21467400	-0.75665600
H	8.75471500	0.41151400	-0.14246800
H	10.21484600	-1.22978300	1.05761200
H	9.05776400	-0.51318500	2.19040200
H	8.79205100	-2.15016500	1.57130700
O	4.96497900	2.36045900	0.33974200
O	4.30106500	-1.88571600	-1.21479900
S	-2.81132800	-0.03518900	-0.88229200
O	-2.65038500	-0.96215100	-1.99090500
O	-2.77393900	1.40611800	-1.07169600
C	-4.28234900	-0.48145900	0.08346300
C	-5.35218200	0.40361100	0.23028100
C	-4.35754100	-1.73182200	0.73499000
C	-6.43499200	0.11416400	1.05817000
C	-5.46554200	-2.03701900	1.54210000
C	-6.48996300	-1.11158500	1.71387600
H	-7.22573400	0.84859600	1.16320400
N	-5.46786400	1.66717100	-0.53655200
O	-5.46975700	1.56434600	-1.75551400
O	-5.64007600	2.68952300	0.11642100
H	-5.50295600	-3.00376200	2.03206800
H	-7.33625600	-1.34885800	2.35010000
C	-3.34984100	-2.74484400	0.60332600
N	-2.59721900	-3.62716400	0.52877900

NI15

C	-1.16270400	-2.68920600	1.71297500

C	-0.26758000	-1.77375300	1.19840300
C	-0.72556800	-0.70290700	0.38880800
C	-2.12273700	-0.59137700	0.11152300
C	-3.01871300	-1.54630300	0.65494500
C	-2.54239300	-2.57682300	1.44493000
H	-0.80260600	-3.50801600	2.32848300
H	0.79342300	-1.86856000	1.39630600
C	0.12528400	0.28782000	-0.17364400
C	-2.60827100	0.46521200	-0.69922300
H	-3.25222300	-3.29206400	1.84700800
C	-1.73300100	1.39894300	-1.22349600
C	-0.35289700	1.31559700	-0.95757600
H	-2.13044800	2.19981600	-1.83752700
H	0.33612300	2.05172900	-1.35148500
O	1.48763700	0.25040100	0.16232400
C	-4.47570400	-1.44909200	0.38346800
C	-4.06029900	0.58597800	-0.98885600
N	-4.89814400	-0.40042900	-0.44871000
C	-6.34254300	-0.29625000	-0.73286900
C	-7.08706600	0.55724700	0.29925400
H	-6.73552600	-1.31398600	-0.74194700
H	-6.43679900	0.13976700	-1.72854300
C	-8.58712500	0.65145800	-0.00661500
H	-6.93895000	0.12069200	1.29520400
H	-6.64792700	1.56292800	0.31303300
C	-9.34987100	1.50176300	1.01433900
H	-8.72765900	1.07309400	-1.01187600
H	-9.01643100	-0.35996500	-0.03464000
H	-10.41768400	1.55168800	0.77310200
H	-9.25495200	1.08637900	2.02499800
H	-8.96506200	2.52852000	1.04021700
O	-5.28349700	-2.23987000	0.85216900
O	-4.51800700	1.49646900	-1.66667000
S	2.54852000	-0.39222400	-0.95319000
O	2.43183500	0.30749300	-2.22079300
O	2.41889800	-1.83991400	-0.86719000
C	4.05174600	0.09954800	-0.02920800
C	5.07902300	-0.85008900	0.08019100
C	4.21237800	1.32583600	0.64797400
C	6.17711400	-0.66532000	0.91269700
C	5.33607000	1.51646500	1.46287700
C	6.29836900	0.52516200	1.61832800
H	6.92239200	-1.44955900	0.97809400
N	5.13536500	-2.07067500	-0.76029300
O	4.97759500	-1.90860200	-1.96428800
O	5.42417300	-3.11928000	-0.19679500
H	5.45016500	2.46293000	1.97664400
H	7.15168000	0.69041800	2.26788800
C	3.25493100	2.51264800	0.57871700
F	2.66176600	2.63046700	-0.62173800
F	2.30286400	2.45402100	1.52481600
F	3.93935700	3.66091000	0.78596500

NI16

C	-1.68918700	-2.40088500	2.22533100
C	-0.74003400	-1.69802200	1.51132200
C	-1.13675800	-0.78612400	0.49969000
C	-2.52925000	-0.61111100	0.23098900
C	-3.48108500	-1.34656200	0.98139100
C	-3.06344100	-2.22568100	1.96431500
H	-1.37606600	-3.09968000	2.99519900
H	0.31599600	-1.84347900	1.70538100
C	-0.22776600	-0.01551400	-0.27693800
C	-2.95460000	0.29071800	-0.77689300
H	-3.81494400	-2.77431800	2.52233400

C	-2.02437100	1.01267600	-1.50242300
C	-0.64695800	0.86319600	-1.25226800
H	-2.37599300	1.69889600	-2.26520200
H	0.08648600	1.43770600	-1.80463100
O	1.13891200	-0.10258200	0.03161500
C	-4.93349000	-1.18069300	0.72083600
C	-4.40054400	0.47420300	-1.06140300
N	-5.29718800	-0.29897300	-0.30917200
C	-6.73657600	-0.13645500	-0.58927800
C	-7.38058500	0.97544200	0.24555700
H	-7.20862600	-1.09646500	-0.37503000
H	-6.82682600	0.08479200	-1.65388900
C	-8.87534100	1.13168100	-0.06107700
H	-7.24283000	0.74678100	1.31003100
H	-6.86028100	1.92015600	0.04267800
C	-9.54042700	2.23681400	0.76561800
H	-9.00618100	1.34607200	-1.13111300
H	-9.38670200	0.17686700	0.12587300
H	-10.60572700	2.32810900	0.52514900
H	-9.45711400	2.03198400	1.83991100
H	-9.07087600	3.20974600	0.57606700
O	-5.78740900	-1.77980300	1.36122500
O	-4.80679100	1.25625800	-1.91090300
S	2.09691200	-1.10039100	-0.91480600
O	1.86710100	-0.81394200	-2.31883200
O	1.95194800	-2.43717400	-0.35964300
C	3.69096000	-0.40204300	-0.31652300
C	4.73560000	-1.29373000	-0.03403500
C	3.86155700	0.94333800	0.02433100
C	5.85683900	-0.86377000	0.67231900
C	5.04714300	1.43704800	0.65255200
C	6.00855100	0.46424700	1.01585700
H	6.63069400	-1.58812500	0.89762300
N	4.78798800	-2.66065500	-0.56565500
O	4.30873800	-2.84201600	-1.68203900
O	5.37596600	-3.50411200	0.10777200
H	6.91953700	0.77448700	1.51126100
N	2.76397800	1.90748300	-0.20811300
O	2.31008000	2.48483300	0.77085500
O	2.41560200	2.08409300	-1.37383000
N	5.26433900	2.76378500	0.92255000
C	6.24592200	3.15139000	1.92850000
H	7.27117500	3.18640200	1.52954400
H	5.98729000	4.14883600	2.29400200
H	6.21637300	2.46734500	2.78003400
C	4.80212500	3.85751200	0.06739900
H	3.99153400	4.42687200	0.53588100
H	5.64512400	4.53045600	-0.12536900
H	4.46065900	3.48085300	-0.89690300

NI17

C	1.56027200	-2.52559700	-2.08003800
C	0.60780900	-1.75144300	-1.44903300
C	0.99710600	-0.77279600	-0.49849200
C	2.38587300	-0.60342600	-0.20671800
C	3.34087200	-1.41380100	-0.87090900
C	2.93050500	-2.35844600	-1.79419500
H	1.25244300	-3.27446600	-2.80354000
H	-0.44516400	-1.89118100	-1.66267200
C	0.08689000	0.07351400	0.19267300
C	2.80467900	0.36820600	0.73701300
H	3.68442700	-2.96323000	-2.28709100
C	1.87253500	1.16381400	1.37777600
C	0.49852400	1.01897500	1.10574300
H	2.21922400	1.90350300	2.09117800

H	-0.23540400	1.65044800	1.59205800
O	-1.27707900	-0.00664700	-0.14208000
C	4.78955000	-1.25563300	-0.58498500
C	4.24720200	0.54788000	1.04300800
N	5.14600900	-0.29981300	0.37910100
C	6.58142400	-0.14056700	0.68118600
C	7.26787300	0.88186300	-0.23073200
H	7.03854100	-1.12431000	0.56370300
H	6.65010800	0.17130800	1.72448500
C	8.75952100	1.03162800	0.09339200
H	7.14582700	0.56633200	-1.27469600
H	6.76569600	1.85140800	-0.12022000
C	9.46476400	2.05082700	-0.80717900
H	8.87508200	1.33053100	1.14475000
H	9.25275300	0.05424700	-0.00431200
H	10.52768900	2.13734800	-0.55480000
H	9.39483500	1.76126600	-1.86277700
H	9.01549000	3.04626200	-0.70583700
O	5.64600200	-1.92188600	-1.15116600
O	4.64772900	1.38890800	1.83679100
S	-2.26852300	-0.96874500	0.79503800
O	-2.00974600	-0.70936600	2.19890600
O	-2.21558300	-2.30532900	0.22296600
C	-3.82963200	-0.18420300	0.23404400
C	-4.97900700	-0.97644200	0.06425400
C	-3.91624100	1.15715500	-0.13178700
C	-6.13105300	-0.45880900	-0.51419200
C	-5.09622400	1.71207300	-0.67908900
C	-6.19840200	0.87759600	-0.88718300
H	-6.98265700	-1.11594300	-0.64436500
N	-5.08473000	-2.35006300	0.58895200
O	-4.54199600	-2.57411200	1.66572000
O	-5.76881600	-3.14226600	-0.05241400
H	-7.11247100	1.26808800	-1.31643300
N	-2.80171100	2.11445400	0.06418700
O	-2.28436100	2.58622300	-0.93470800
O	-2.52472600	2.38013500	1.23020600
O	-5.05961700	3.02736900	-0.94469500
C	-6.21139300	3.65602100	-1.51140200
H	-5.93433700	4.70233000	-1.63671600
H	-6.45545600	3.21985300	-2.48646400
H	-7.07238400	3.57952900	-0.83767100

CDNB

C	-1.08805300	-0.92115400	-0.02061200
C	-0.70076000	0.42939800	0.00154600
C	-0.09525300	-1.90922700	-0.02871600
C	0.64500800	0.78536800	0.00275000
C	1.25085600	-1.56712300	0.00010300
H	-0.39537800	-2.95049700	-0.06024300
C	1.60168700	-0.21876900	0.01514200
H	0.93320800	1.82834200	-0.00421400
N	-1.66671100	1.54658700	0.03222300
O	-2.71034100	1.38409000	0.65303300
O	-1.32986500	2.57649300	-0.54776400
H	2.02528000	-2.32416600	0.00364800
N	3.02629500	0.15580100	0.03737400
O	3.84779800	-0.75860800	0.03334300
O	3.29673100	1.35376600	0.05885800
Cl	-2.74092300	-1.44160500	-0.10749400

NNBA

C	-6.45095500	-0.06508900	0.15923300
C	-5.80531600	-1.23497300	0.56406000
C	-5.68924500	1.01478600	-0.28804600
C	-4.41699300	-1.31907700	0.51974900
C	-4.29652600	0.94810400	-0.33833000
H	-6.18042600	1.93075400	-0.60480800
C	-3.65450800	-0.23016000	0.06983900
H	-3.91945700	-2.23433200	0.83600900
H	-3.70923100	1.78849400	-0.67939000
N	-2.24928200	-0.39981100	0.04500600
C	-1.27909800	0.51884800	-0.26574700
O	-1.49166000	1.68771000	-0.56266500
C	0.13727600	-0.00576300	-0.20848100
C	0.49077000	-1.34717800	-0.39564400
C	1.14530200	0.94297700	0.00787800
C	1.82773000	-1.74109800	-0.35372500
H	-0.26320700	-2.09571200	-0.62032000
C	2.47197300	0.54677300	0.07146800
H	0.88227200	1.98665000	0.13406800
C	2.81818700	-0.79539000	-0.11907200
H	2.11821300	-2.77196100	-0.51919700
N	3.46776700	1.56858300	0.45257100
N	4.21911900	-1.24767200	-0.19983500
O	3.33850800	2.68011300	-0.04768600
O	4.30690700	1.23729600	1.28239100
O	4.46639200	-2.35989800	0.25779600
O	5.01254300	-0.50450400	-0.76475100
H	-1.92942200	-1.29005000	0.40079700
H	-6.38077700	-2.08666800	0.91579400
H	-7.53429800	0.00284200	0.19309100

DNAF1

C	-6.00867500	-3.29376300	-1.13955400
C	-6.60503700	-2.04291200	-0.98550300
C	-4.62057500	-3.44725900	-0.96711900
C	-5.80825300	-0.94870800	-0.65452700
H	-7.67348000	-1.89613200	-1.11597200
C	-3.84891400	-2.34872700	-0.63852800
H	-4.18224400	-4.43076800	-1.09619600
C	-4.41489400	-1.06618100	-0.46946600
H	-2.77881300	-2.47063700	-0.50785800
C	-5.75730800	1.39247600	-0.21493200
C	-3.66653700	0.12001200	-0.12225800
C	-6.46978900	2.54323700	-0.11564300
C	-4.31908900	1.32778000	-0.01310800
C	-5.81718500	3.80691500	0.21199300
H	-7.54228500	2.54532300	-0.27537700
C	-3.66562400	2.57773200	0.31674000
C	-4.35611600	3.73566900	0.42224500
H	-2.59221700	2.56045300	0.47834600
H	-3.86252500	4.67092900	0.66940600
C	-2.17943700	0.03001800	0.02015100
C	-1.51574200	-0.32860400	1.21436300
C	-1.39246400	0.31389900	-1.10326400
C	-0.11863000	-0.38790600	1.24529800
C	-0.00125600	0.25306300	-1.08013500
H	-1.88681300	0.59484500	-2.02835700
C	0.64798300	-0.10357600	0.11071200
H	0.36803000	-0.65985500	2.17641100
H	0.57794800	0.48364300	-1.96276200
O	-6.44681800	0.24244400	-0.52493200
C	-2.29027300	-0.64156800	2.45089800
O	-3.49869200	-0.68750100	2.53314900

O	-1.48916700	-0.88471800	3.52215000
H	-2.09408300	-1.07427700	4.26333000
O	-6.71836900	-4.40762700	-1.46128700
O	-6.43207000	4.87397900	0.31043300
N	2.04971400	-0.20610300	0.23756000
C	3.01344500	0.08160200	-0.70142800
O	2.77836300	0.51272900	-1.82191200
C	4.43485500	-0.16179300	-0.25593700
C	4.80505500	-1.08511000	0.72894100
C	5.42971600	0.56890700	-0.91857000
C	6.14727900	-1.26971700	1.05844400
H	4.06180200	-1.70503400	1.22138600
C	6.76172500	0.39993600	-0.57422400
H	5.15509300	1.27239800	-1.69577200
C	7.12504700	-0.52733200	0.40791000
H	6.45089900	-1.99622300	1.80307500
N	7.74557900	1.30454000	-1.20348100
N	8.53253600	-0.84521600	0.71482200
O	7.59388600	1.54074200	-2.39648200
O	8.59719400	1.78832300	-0.46698400
O	8.79652100	-1.09576800	1.88700100
O	9.31056400	-0.88870000	-0.23007500
H	2.37393500	-0.42827400	1.16887900
H	-7.65717700	-4.17868800	-1.55032300

DNAF2

C	4.65812400	-1.38150500	3.58570700
C	5.00501700	-0.12964700	3.07929100
C	3.76896200	-2.21387600	2.88101600
C	4.46177100	0.28416200	1.86465100
H	5.68784800	0.53290800	3.60359700
C	3.24112600	-1.78383300	1.67827400
H	3.51546400	-3.18222100	3.29829400
C	3.56879200	-0.52436300	1.13082200
H	2.55595700	-2.42786000	1.13719000
C	4.35371900	2.02674400	0.24354200
C	3.05726000	-0.01763000	-0.12065400
C	4.77385800	3.26489500	-0.12025200
C	3.42688100	1.23688900	-0.55000100
C	4.31130600	3.88319200	-1.35852000
H	5.46579000	3.81957200	0.50370500
C	2.96392800	1.84424700	-1.78044300
C	3.37105400	3.07562200	-2.16327800
H	2.27183300	1.27771800	-2.39576000
H	3.02212200	3.52781200	-3.08699700
C	2.06036100	-0.83306800	-0.88719400
C	2.41976000	-1.78833800	-1.86348100
C	0.71012500	-0.60661600	-0.59892600
C	1.40413900	-2.48507100	-2.53764600
C	-0.29302200	-1.31798500	-1.27401600
H	0.43440100	0.12861500	0.14342900
C	0.06808500	-2.25958000	-2.25051700
H	1.67628100	-3.21211100	-3.29336200
O	4.83638800	1.51296300	1.42551500
C	3.84779600	-2.06267400	-2.17823500
O	4.80450800	-1.59608700	-1.59576700
O	3.99413000	-2.92700500	-3.21757900
H	4.95629800	-3.03728200	-3.33165700
O	5.14693000	-1.85516000	4.76255200
O	4.67238000	5.00711800	-1.72342800
H	5.74686100	-1.19871500	5.15095300
N	-1.66699900	-1.14741500	-1.02239400
C	-2.28090500	-0.27475000	-0.14859700
O	-1.68786400	0.54293500	0.53897200
C	-3.78547400	-0.37987800	-0.09197900

C	-4.50739400	-1.53718800	-0.40704400
C	-4.47347900	0.75819400	0.34895100
C	-5.89711400	-1.55743700	-0.29704800
H	-3.99923100	-2.45176700	-0.69783200
C	-5.85673600	0.74254900	0.43789200
H	-3.92251400	1.65309900	0.61359400
C	-6.57140700	-0.41824900	0.12476200
H	-6.46792300	-2.45198900	-0.51708500
N	-6.53078700	2.01480500	0.76909200
N	-8.02767600	-0.53226100	0.33202300
O	-6.00926500	2.70530200	1.63679400
O	-7.52332500	2.29915700	0.10900500
O	-8.64922000	-1.19803300	-0.49064100
O	-8.48828600	-0.00296800	1.33571000
H	-0.69997700	-2.81546100	-2.78386000
H	-2.27517400	-1.68286200	-1.62676700

ANs-AcRh

S	-3.38346600	-1.33432100	1.41870700
O	-3.00289900	-0.25272000	2.31666600
O	-3.80128100	-2.66322600	1.85815000
C	-4.64565600	-0.71927700	0.24996900
C	-6.02115500	-0.68876100	0.54566600
C	-4.23844700	-0.30008200	-1.01691100
C	-6.94896400	-0.26262100	-0.39265800
C	-5.16674600	0.15772000	-1.95374600
H	-3.18929400	-0.34545400	-1.28010700
C	-6.53117000	0.17828500	-1.65370100
H	-8.00425700	-0.26340400	-0.14765200
N	-6.55740700	-1.06717200	1.87087800
O	-5.81766000	-0.91744800	2.83714300
O	-7.71589400	-1.46981600	1.91188500
H	-4.80781000	0.48666700	-2.92338500
N	-2.07592000	-1.64874100	0.36782400
H	-1.95800200	-2.65631000	0.31181200
C	-0.87416200	-0.89862100	0.31590400
C	0.32912700	-1.58982900	0.18525100
C	-0.86910500	0.50718600	0.31876200
C	1.52528800	-0.88326500	0.04101300
H	0.36522000	-2.67497100	0.19013500
C	0.33548900	1.18259000	0.19669000
H	-1.79015800	1.06267900	0.44117300
C	1.55906300	0.51334300	0.04961300
H	0.32947100	2.26820800	0.20444200
C	3.87498300	-1.05309400	-0.15467700
C	2.85290900	1.27164300	-0.15678800
C	4.95988900	-1.93390700	-0.19141500
C	4.04006000	0.33330200	-0.15138300
C	6.25872300	-1.43029900	-0.26924500
H	4.75603900	-2.99448200	-0.13180300
C	5.35662300	0.81834000	-0.20079600
C	6.44627800	-0.03260400	-0.26314400
H	5.52201700	1.89139400	-0.20529700
H	7.45206300	0.37324300	-0.32457700
C	3.01378500	2.45802500	0.78434600
C	3.03583900	3.63126600	0.04135700
C	3.12722300	2.49374300	2.17150200
C	3.17135800	4.88329600	0.63970300
C	3.26314800	3.74294600	2.78325700
H	3.10866400	1.58335400	2.76370500
C	3.28600200	4.92673300	2.02844800
H	3.18485700	5.78341800	0.03254000
H	3.35170600	3.79955100	3.86468900
H	3.39278200	5.88177900	2.53465700
O	2.64613000	-1.65867500	-0.08765700

C	2.89253100	3.28966300	-1.39301500
O	2.86034000	4.01509900	-2.35601600
O	2.79073100	1.92418500	-1.48689000
N	7.40252000	-2.24648300	-0.29712700
H	8.25429300	-1.82490700	0.05707800
C	7.66732600	-3.49457600	-0.85242900
C	6.56665400	-4.22020900	-1.60006400
H	5.93527200	-4.78292200	-0.90254800
H	5.92502800	-3.54538100	-2.17295600
H	7.04844500	-4.93565400	-2.26842900
O	8.78959200	-3.96113000	-0.73638200
C	-7.58813900	0.65111700	-2.62089900
C	-7.15962000	1.12627900	-3.99520000
H	-6.47098100	1.97667400	-3.92092800
H	-6.64087600	0.33068900	-4.54374400
H	-8.04698500	1.42889600	-4.55299700
O	-8.75921100	0.64533700	-2.28547300

BNs-AcRh

S	-2.11314300	-3.02786300	0.19145300
O	-1.64031700	-2.98994600	1.56855700
O	-2.34330600	-4.25425200	-0.56825000
C	-3.61697800	-1.99967200	0.04890800
C	-4.90686500	-2.47473400	0.34673100
C	-3.49065900	-0.69146800	-0.42248500
C	-6.02678300	-1.67276600	0.17180600
C	-4.60839600	0.12955900	-0.57285600
H	-2.51107800	-0.31619400	-0.68946300
C	-5.88323600	-0.35880400	-0.27958200
H	-7.01115400	-2.06663100	0.39297200
N	-5.15143800	-3.82555600	0.89392700
O	-4.24441600	-4.34246300	1.53686600
O	-6.25765000	-4.31916800	0.69493000
H	-4.48825200	1.14604700	-0.92898400
N	-1.01394600	-2.15394900	-0.77925500
H	-0.83994100	-2.69888800	-1.61880000
C	0.09990000	-1.42503500	-0.29285300
C	1.30292400	-1.51533100	-0.99099300
C	0.00583500	-0.56700600	0.81689600
C	2.39680500	-0.74216300	-0.59565700
H	1.41621000	-2.17508800	-1.84582400
C	1.11446300	0.17160700	1.20082000
H	-0.91313500	-0.49663800	1.38481200
C	2.33254900	0.11125200	0.50842700
H	1.03290200	0.82814200	2.06166200
C	4.67057900	-0.23659400	-0.99884900
C	3.50725100	0.98070100	0.90297300
C	5.78674200	-0.53162800	-1.78730300
C	4.73453500	0.64164200	0.08461600
C	7.00542500	0.09494000	-1.52542100
H	5.67386600	-1.26691500	-2.57254100
C	5.97774300	1.23816800	0.34856200
C	7.09094200	0.98267100	-0.43312100
H	6.06319700	1.93058900	1.18052100
H	8.03327100	1.47857700	-0.21814300
C	3.76732800	0.99922400	2.40339900
C	3.56916400	2.28628100	2.88605100
C	4.14232100	-0.03096000	3.26148700
C	3.73540000	2.60493000	4.23318300
C	4.31184200	0.27564800	4.61449300
H	4.29681200	-1.04233000	2.89660300
C	4.11189000	1.57857400	5.09829300
H	3.57309300	3.62061100	4.58118300
H	4.60269500	-0.51013300	5.30622700
H	4.25131600	1.78372700	6.15576000

O	3.52539100	-0.90198100	-1.35423200
C	3.17490200	3.16238700	1.75842200
O	2.91145300	4.33937700	1.75587100
O	3.14512600	2.39107800	0.62353100
N	8.17556500	-0.15489100	-2.26324700
H	9.04902800	-0.00162100	-1.77104800
C	8.41095600	-0.43160700	-3.60636200
C	7.23617300	-0.51745100	-4.55999100
H	6.79634600	-1.52134100	-4.53733100
H	6.44860700	0.20521800	-4.33037600
H	7.62292500	-0.34280200	-5.56530500
O	9.56351600	-0.57956600	-3.98113200
C	-7.12520900	0.46643700	-0.42673600
O	-8.23757400	0.05572300	-0.16827900
O	-6.85755100	1.70562400	-0.87361700
C	-8.00114200	2.58287500	-1.04094900
H	-8.69561000	2.11704700	-1.74782000
H	-8.51519600	2.67014900	-0.07810100
C	-7.48831200	3.92354800	-1.54026000
H	-6.94970900	3.77247700	-2.48498800
H	-6.75957300	4.31922300	-0.82062700
C	-8.62439200	4.93599900	-1.74266300
H	-9.35528400	4.52765200	-2.45438700
H	-9.16377000	5.07354700	-0.79530100
C	-8.12323000	6.29307400	-2.24676900
H	-7.60790700	6.19260900	-3.20950700
H	-8.95284200	6.99501600	-2.38372200
H	-7.41799500	6.74334000	-1.53798100

CNs-AcRh

S	-3.68151000	-1.20124000	1.21027300
O	-3.31547500	-0.07714100	2.05978700
O	-4.12363900	-2.50289700	1.70326600
C	-4.92204300	-0.63578300	-0.01122700
C	-6.29899700	-0.57084700	0.26129800
C	-4.48976300	-0.29576600	-1.29569300
C	-7.21390400	-0.19162200	-0.71269600
C	-5.39065600	0.11462300	-2.27525300
H	-3.43670000	-0.36854200	-1.53605400
C	-6.76146900	0.16444300	-1.98949300
H	-8.26774400	-0.17506800	-0.46465900
N	-6.86586300	-0.86491500	1.59675500
O	-6.13409000	-0.69656000	2.56398600
O	-8.03958700	-1.22227100	1.63771400
H	-5.03302600	0.38427500	-3.26316100
N	-2.36115600	-1.56845400	0.19705700
H	-2.25323200	-2.57886700	0.18180100
C	-1.14492900	-0.83549100	0.16065600
C	0.05303300	-1.54593800	0.13701000
C	-1.12361000	0.56685500	0.07459400
C	1.26452000	-0.86126800	0.01143000
H	0.07461700	-2.62893800	0.21094200
C	0.09423100	1.22221700	-0.02401900
H	-2.04334900	1.13723800	0.10950700
C	1.31472800	0.53282900	-0.06334200
H	0.10234000	2.30609500	-0.08471300
C	3.61784300	-1.06653700	-0.07671200
C	2.62748500	1.26769600	-0.23833500
C	4.69160600	-1.96023600	-0.02845600
C	3.80044600	0.31540800	-0.14998500
C	5.99863200	-1.47690100	-0.09718700
H	4.47216500	-3.01305500	0.08698500
C	5.12408100	0.78194500	-0.18858400
C	6.20385100	-0.08338000	-0.16844500
H	5.30335400	1.85093600	-0.25148700

H	7.21611000	0.30686400	-0.22413300
C	2.76382200	2.48026500	0.67365500
C	2.83245300	3.62994600	-0.10251400
C	2.82098900	2.55629400	2.06254000
C	2.96014000	4.89784400	0.46340400
C	2.94860600	3.82172500	2.64188500
H	2.76722100	1.66452500	2.68039800
C	3.01807000	4.98177500	1.85377100
H	3.01112300	5.77893300	-0.16912400
H	2.99407600	3.90999400	3.72385700
H	3.11700500	5.95029700	2.33533000
O	2.37942700	-1.65400100	-0.01218200
C	2.74650100	3.24624400	-1.53063900
O	2.76567700	3.94080400	-2.51602800
O	2.63076500	1.87865700	-1.58711400
N	7.13159700	-2.30648400	-0.04355100
H	7.97849900	-1.87491700	0.31035600
C	7.39546700	-3.58760500	-0.51905000
C	6.30710800	-4.34119400	-1.25696900
H	5.65120000	-4.85976500	-0.54787800
H	5.68872100	-3.69159400	-1.88205200
H	6.79834100	-5.09608900	-1.87303700
O	8.50730400	-4.06005800	-0.34323700
C	-7.69874400	0.57320500	-2.99530500
N	-8.45266400	0.90567600	-3.81523600

DNs-AcRh

S	-3.37422000	-1.27060900	1.37438000
O	-2.98818000	-0.16099800	2.23359500
O	-3.79186900	-2.58534200	1.85336600
C	-4.66478300	-0.68874800	0.21247300
C	-6.03142900	-0.65023600	0.54053600
C	-4.28192400	-0.30976600	-1.07713500
C	-6.98805500	-0.25710900	-0.38736900
C	-5.22347400	0.11597600	-2.01301400
H	-3.23772500	-0.36340800	-1.35753500
C	-6.56435700	0.13112200	-1.65137900
H	-8.03720700	-0.25310900	-0.12321200
N	-6.54337400	-0.98794200	1.88791100
O	-5.78388000	-0.81591500	2.83304400
O	-7.70366700	-1.38064900	1.95898500
H	-4.92946800	0.42042200	-3.00992200
N	-7.57462800	0.56745900	-2.63764400
O	-7.16730900	0.89106500	-3.75013400
O	-8.74747500	0.57618100	-2.27613500
N	-2.08958800	-1.60408800	0.30680000
H	-1.97636100	-2.61311500	0.26221500
C	-0.87851000	-0.86257000	0.24948700
C	0.32224500	-1.56464500	0.17698800
C	-0.86840300	0.54118300	0.19229000
C	1.52571800	-0.86942200	0.03248900
H	0.35241200	-2.64879000	0.22737600
C	0.34225600	1.20664100	0.07454100
H	-1.79025300	1.10467600	0.26448100
C	1.56529900	0.52630400	-0.01264900
H	0.34206100	2.29158000	0.03700600
C	3.87695500	-1.05705000	-0.12411500
C	2.86860000	1.27373800	-0.20510300
C	4.95698400	-1.94445300	-0.12593300
C	4.04910800	0.32737600	-0.16986000
C	6.25870600	-1.45081500	-0.21822200
H	4.74729600	-3.00120100	-0.02967100
C	5.36835000	0.80358700	-0.23302300
C	6.45348900	-0.05468300	-0.26205900
H	5.53930100	1.87493400	-0.27565200

H	7.46150600	0.34347700	-0.33566600
C	3.02140300	2.46563400	0.73127700
C	3.06307100	3.63336500	-0.01947800
C	3.11298500	2.51010200	2.11972100
C	3.19689700	4.88873900	0.57229600
C	3.24693000	3.76272400	2.72488100
H	3.08013400	1.60406800	2.71799600
C	3.28922000	4.94104500	1.96238500
H	3.22619700	5.78452900	-0.04065800
H	3.31884300	3.82637200	3.80714900
H	3.39394100	5.89888900	2.46366700
O	2.64409600	-1.65401800	-0.03959100
C	2.94235700	3.28226200	-1.45347500
O	2.93226000	3.99928400	-2.42268700
O	2.83257900	1.91553100	-1.53860000
N	7.39765300	-2.27386100	-0.21419400
H	8.25087800	-1.84547900	0.12820300
C	7.65692200	-3.54087400	-0.72856600
C	6.55452100	-4.28291800	-1.45730300
H	5.91846400	-4.82140500	-0.74501000
H	5.91802900	-3.62228400	-2.05198600
H	7.03432900	-5.02052500	-2.10260300
O	8.77572100	-4.01012800	-0.59315000

Cartesian coordinates (angstrom) for the optimized structures of NI9 and NI used for TD-DFT calculations.

NI9

C	-0.53319200	2.89803700	0.21641600
C	0.26804300	1.83286700	-0.14196300
C	-0.27916800	0.52831000	-0.24462400
C	-1.66838000	0.33401400	0.02530500
C	-2.46765500	1.44611700	0.39178700
C	-1.90371700	2.70709700	0.48417400
H	-0.10760100	3.89385600	0.29256600
H	1.32033200	1.98731600	-0.35267400
C	0.47823000	-0.61855000	-0.61135600
C	-2.24093800	-0.95941800	-0.07372400
H	-2.53107600	3.54661700	0.76423700
C	-1.45680300	-2.03953900	-0.43914800
C	-0.08697700	-1.87205500	-0.71587400
H	-1.90824300	-3.02230800	-0.51879200
H	0.51955500	-2.71791300	-1.01803000
O	1.82094000	-0.42457500	-0.94909600
C	-3.91181300	1.26629600	0.67499400
C	-3.68266900	-1.16299400	0.20501400
N	-4.42420200	-0.03542600	0.58129100
C	-5.86046600	-0.22563600	0.86421000
C	-6.73551200	-0.06492300	-0.38297200
H	-6.13494400	0.50939300	1.62172700
H	-5.97170900	-1.22603400	1.28402200
C	-8.22307000	-0.26646300	-0.07066600
H	-6.57837200	0.93587900	-0.80551800
H	-6.41519100	-0.78961600	-1.14262700
C	-9.11519800	-0.10811200	-1.30448100
H	-8.37114800	-1.26570500	0.36266800
H	-8.53314800	0.45301000	0.70031900
H	-10.17213900	-0.25797100	-1.05317200
H	-9.01527700	0.89391500	-1.74084800
H	-8.85107300	-0.83648300	-2.08176600
O	-4.64213100	2.20293400	0.98278300
O	-4.21885200	-2.26326700	0.11741600

S	2.96469600	-0.97721300	0.15129700
O	3.06834500	-2.42491400	-0.00405600
O	2.63140600	-0.38559300	1.44331800
C	4.36858200	-0.13103600	-0.61172000
C	5.51041100	0.23606700	0.12247700
C	4.30277200	0.20522000	-1.96419200
C	6.54213700	0.95752200	-0.46907800
C	5.35222700	0.90550100	-2.56551500
H	3.43228400	-0.05830700	-2.55010900
C	6.46331100	1.29038100	-1.82040200
H	7.40706500	1.22719900	0.12521800
N	5.71203800	-0.18113100	1.51423000
O	5.25564200	-1.27525300	1.84812700
O	6.35713100	0.56223400	2.25098100
H	5.28456600	1.15348800	-3.61981000
H	7.27534200	1.84023000	-2.28457300

NI

C	-2.73589600	2.63868200	0.24315500
C	-3.47668000	1.46391900	0.35002100
C	-2.87754200	0.20444300	0.20461000
C	-1.47619900	0.12826500	-0.06085100
C	-0.73201600	1.33430900	-0.16719200
C	-1.36192200	2.56853800	-0.01446000
H	-3.21867700	3.60526800	0.35942400
H	-4.54416100	1.49340300	0.55053300
C	-3.69863800	-1.02640700	0.32757200
C	-0.83724100	-1.13093500	-0.21229400
H	-0.76441800	3.47100200	-0.10004700
C	-1.62047700	-2.30124400	-0.09138500
C	-2.97459400	-2.26600200	0.16345500
H	-1.11483400	-3.25718700	-0.20821400
H	-3.54608800	-3.18757400	0.24898200
O	-4.92909300	-0.97407200	0.55792300
C	0.71800500	1.28506700	-0.43563100
C	0.58066300	-1.21613100	-0.47988200
N	1.28239500	0.01787800	-0.59422100
C	2.72528500	-0.04989500	-0.87177600
C	3.57673700	-0.10507300	0.40141500
H	2.98224400	0.83322600	-1.45927700
H	2.89195100	-0.94363100	-1.47442700
C	5.07754000	-0.17313400	0.09537900
H	3.36412900	0.78146900	1.01308300
H	3.27910800	-0.98247200	0.99031200
C	5.94218900	-0.23088900	1.35747400
H	5.28183600	-1.05521800	-0.52829100
H	5.36605000	0.70120900	-0.50552600
H	7.00943400	-0.27858400	1.10883600
H	5.78626900	0.65494400	1.98649500
H	5.70175600	-1.11384400	1.96346200
O	1.41671000	2.30063900	-0.52327600
O	1.20905300	-2.27509400	-0.61508000

References

1. Rassolov, V. A.; Ratner, M. A.; Pople, J. A.; Redfern, P. C.; Curtiss, L. A., 6-31G*basis set for third-row atoms. *J. Comput. Chem.* **2001,** *22*, 976−984.
2. Lu, T.; Chen, F., Multiwfn: a multifunctional wavefunction analyzer. *J. Comput. Chem.* **2012,** *33*, 580−592.
3. Gaussian 09, Revision D.01, M. J. Frisch *et al.* Gaussian, Inc., Wallingford CT, **2013**.
4. Weigend, F.; Ahlrichs, R., Balanced basis sets of split valence, triple zeta valence and quadruple zeta valence quality for H to Rn: Design and assessment of accuracy. *Phys. Chem. Chem. Phys.* **2005,** *7*, 3297−3305.
5. Parr, R. G.; Von Szentpaly, L.; Liu, S. B., Electrophilicity index. *J. Am. Chem. Soc.* **1999,** *121*, 1922−1924.
6. Weigend, F., Accurate Coulomb-fitting basis sets for H to Rn. *Phys. Chem. Chem. Phys.* **2006,** *8*, 1057−1065.
7. Hellweg, A.; Hattig, C.; Hofener, S.; Klopper, W., Optimized accurate auxiliary basis sets for RI-MP2 and RI-CC2 calculations for the atoms Rb to Rn. *Theor. Chem. Acc.* **2007,** *117*, 587−597.
8. Grimme, S.; Antony, J.; Ehrlich, S.; Krieg, H., A consistent and accurate ab initio parametrization of density functional dispersion correction (DFT-D) for the 94 elements H-Pu. *J. Chem. Phys.* **2010,** *132*, 154104.
9. Zhang, J.; Shibata, A.; Ito, M.; Shuto, S.; Ito, Y.; Mannervik, B.; Abe, H.; Morgenstern, R., Synthesis and characterization of a series of highly fluorogenic substrates for glutathione transferases, a general strategy. *J. Am. Chem. Soc.* **2011,** *133*, 14109−14119.
10. Marenich, A. V.; Cramer, C. J.; Truhlar, D. G., Universal solvation model based on solute electron density and on a continuum model of the solvent defined by the bulk dielectric constant and atomic surface tensions. *J. Phys. Chem. B* **2009,** *113*, 6378−6396.
11. Kendall, R. A.; Dunning, T. H.; Harrison, R. J., Electron-Affinities of the 1st-Row Atoms Revisited - Systematic Basis-Sets and Wave-Functions. *J. Chem. Phys.* **1992,** *96*, 6796−6806.
12. Woon, D. E.; Dunning, T. H., Gaussian-Basis Sets for Use in Correlated Molecular Calculations .3. The Atoms Aluminum through Argon. *J. Chem. Phys.* **1993,** *98*, 1358−1371.
13. Dunning, T. H., Gaussian-Basis Sets for Use in Correlated Molecular Calculations .1. The Atoms Boron through Neon and Hydrogen. *J. Chem. Phys.* **1989,** *90*, 1007−1023.
14. Gaussian 16, Revision A.03, M. J. Frisch *et al.* Gaussian, Inc., Wallingford CT, **2016**.
15. Buhr, W. *et al.* Novel sulfonaminoquinoline derivatives as hepcidin antagonists and their preparation and use in the treatment of iron metabolic disorders. Patent, *WO 2012110603 A1* **2014**.
16. Fujikawa, Y.; Urano, Y.; Komatsu, T.; Hanaoka, K.; Kojima, H.; Terai, T.; Inoue, H.; Nagano, T., Design and synthesis of highly sensitive fluorogenic substrates for glutathione S-transferase and application for activity imaging in living cells. *J. Am. Chem. Soc.* **2008,** *130*, 14533−14543.

17. Shibata, A.; Nakano, Y.; Ito, M.; Araki, M.; Zhang, J.; Yoshida, Y.; Shuto, S.; Mannervik, B.; Morgenstern, R.; Ito, Y.; Abe, H., Fluorogenic probes using 4-substituted-2-nitrobenzenesulfonyl derivatives as caging groups for the analysis of human glutathione transferase catalyzed reactions. *Analyst* **2013,** *138*, 7326−7330.
18. Zhou, W.; Shultz, J. W.; Murphy, N.; Hawkins, E. M.; Bernad, L.; Good, T.; Moothart, L.; Frackman, S.; Klaubert, D. H.; Bulleit, R. F.; Wood, K. V., Electrophilic aromatic substituted luciferins as bioluminescent probes for glutathione S-transferase assays. *Chem. Commun.* **2006**, 4620−4622.
19. Roy, R. K.; Hirao, K.; Pal, S., On non-negativity of Fukui function indices. II. *J. Chem. Phys.* **2000,** *113*, 1372−1379.
20. Roy, R. K.; Hirao, K.; Krishnamurty, S.; Pal, S., Mulliken population analysis based evaluation of condensed Fukui function indices using fractional molecular charge. *J. Chem. Phys.* **2001,** *115*, 2901−2907.
21. Ji, X.; Armstrong, R. N.; Gilliland, G. L., Snapshots along the reaction coordinate of an SNAr reaction catalyzed by glutathione transferase. *Biochemistry* **1993,** *32*, 12949−12954.
22. Armstrong, R. N., Structure, catalytic mechanism, and evolution of the glutathione transferases. *Chem. Res. Toxicol.* **1997,** *10*, 2−18.
23. Johnson, W. W.; Liu, S. X.; Ji, X. H.; Gilliland, G. L.; Armstrong, R. N., Tyrosine-115 Participates Both in Chemical and Physical Steps of the Catalytic Mechanism of a Glutathione-S-Transferase. *J. Biol. Chem.* **1993,** *268*, 11508−11511.
